# Light regulates tomato fruit metabolome via SlDML2‐mediated global DNA demethylation

**DOI:** 10.1111/jipb.70066

**Published:** 2025-10-23

**Authors:** Zixin Zhang, Jing Zhang, Yi Wang, Yuting Chen, Qian Hu, Xin Zhang, Wen Li, Yiren Xiao, Ke Zhou, Yanling Lai, Dan Su, Evangelos Tatsis, Gaofeng Liu, Mingchun Liu, Zhenhui Zhong, Yang Zhang

**Affiliations:** ^1^ Key Laboratory of Bio‐resource and Eco‐environment of Ministry of Education, Chengdu Botanical Garden-Sichuan University Joint Laboratory for Ex Situ Conservation and Resource Utilization of Mountain Plants, College of Life Sciences Sichuan University Chengdu 610065 China; ^2^ College of Horticulture and Landscape Architecture Southwest University Chongqing 400715 China; ^3^ College of Life Sciences Shandong Agricultural University Taian 271018 China; ^4^ National Key Laboratory of Plant Molecular Genetics Chinese Academy of Sciences Center for Excellence in Molecular Plant Sciences Shanghai 200032 China; ^5^ Chinese Academy of Sciences‐John Innes Center of Excellence for Plant and Microbial Science Shanghai 200032 China; ^6^ Beijing Key Laboratory of Growth and Developmental Regulation for Protected Vegetable Crops College of Horticulture China Agricultural University Beijing 100193 China; ^7^ School of Breeding and Multiplication, Sanya Institute of Breeding and Multiplication Hainan University Sanya 572025 China

**Keywords:** DNA demethylation, light signaling, SlDML2, SlHY5, tomato fruit

## Abstract

Modifying the light formula is a central strategy for improving the yield and quality of fruits and vegetables in agriculture. While light signals have long been acknowledged as primary factors in regulating plant growth and development, their role in reprogramming metabolic networks is not well understood. Using tomato as a model, we demonstrate that supplementation with red or blue light induces metabolic shifts in tomato fruit. Through the creation of the Tomato Light‐induced Expression Database (TomLED), we identified extensive transcriptomic and metabolic changes in tomato fruit under varying light conditions. Notably, the induction of key master regulators and metabolic genes is mediated by increased genome‐wide DNA demethylation, facilitated by SlDML2. Additionally, we show that SlHY5, a central regulator in the light signaling pathway, directly induces the expression of *SlDML2*. This study reveals the molecular mechanisms by which light regulates the plant epigenome and establishes a direct link between light signals and plant metabolism.

## Graphical abstract

In tomato growth and development, red and blue light signals are captured by the photoreceptors phyB2 and CRY1a, which kickstart tomato fruit ripening by activating HY5, which activates DNA demethylation via DML2 to trigger a metabolic and ripening transformation cascade within the tomato.

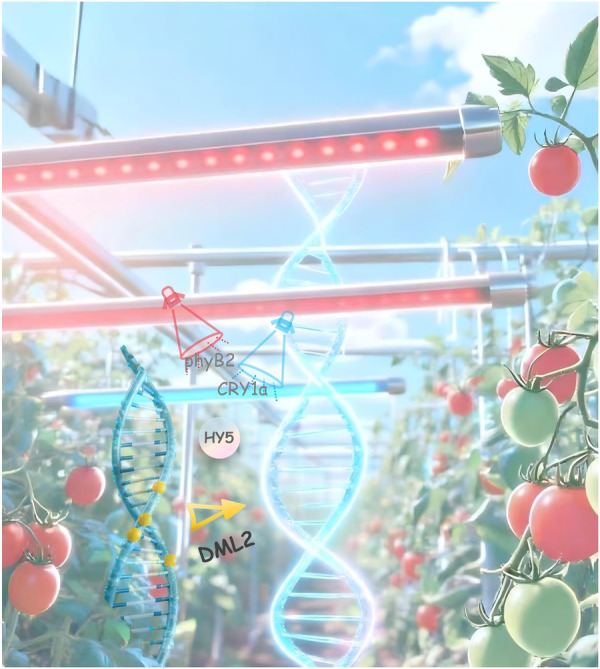

## INTRODUCTION

Light is the primary energy source for plants and serves as a vital environmental factor with extensive biological roles (Zhao et al., 2023). Variations in light duration (photoperiod), photon flux (light intensity), and spectral wavelength (light quality) can profoundly impact plant growth and metabolism (Vitale et al., 2022). In contemporary agricultural systems, adjusting the light formula, including photoperiod, intensity, and quality, is recognized as an essential approach to accelerate growth rates, increase yield, or improve quality (Watson et al., 2018; Vitale et al., 2022; Walter and Kromdijk, 2022). Numerous studies have highlighted that modifying the light formula is crucial for enhancing the yield and quality of fruits and vegetables (Alba et al., 2000a; Gil et al., 2020; He et al., 2022; Wang et al., 2022b). Significant progress has been made in understanding the molecular mechanisms by which light influences plant growth and development, particularly in photomorphogenesis studies with model plants like Arabidopsis (Lian et al., 2011; Pham et al., 2018; Paik and Huq, 2019; Xie et al., 2019; Jing and Lin, 2020; Yang and Liu, 2020), but how epigenetic regulation is involved in light response has not been explored.

Plants contain various photoreceptors, including phytochromes (red/far‐red light), cryptochromes (blue light), phytotropins (blue light), and UV resistance locus 8 (ultraviolet B light), among others, which allow them to sense specific light wavelengths (Sharrock and Quail, 1989; Lian et al., 2011; Christie et al., 2012; Favory et al., 2014; Pham et al., 2018; Paik and Huq, 2019; Gao et al., 2022a). Upon detecting light signals, these photoreceptors trigger a series of signal transduction pathways that activate or inhibit downstream components, thereby modulating the expression of specific genes (Pham et al., 2018; Jing and Lin, 2020; Jia et al., 2023). Key downstream components in plants include COP1 (Constitutively Photomorphogenic 1), HY5 (Elongated Hypocotyl 5), PIFs (Phytochrome Interacting Factors), LAF1 (Long After Far‐Red light 1), HFR1 (Long Hypocotyl in Far‐Red 1), and BBXs (B‐Box Proteins), among others (Arnim and Deng, 1994; Leivar et al., 2008; Xu, 2019; Jing and Lin, 2020; Song et al., 2020, 2021; Bian et al., 2022; Lan et al., 2022; Li et al., 2022; Gao et al., 2022a; Diao et al., 2023; Jia et al., 2023). HY5 and PIFs play a central role in the light‐temperature signaling pathway (Bian et al., 2022; Qi et al., 2022). HY5 is capable of directly or indirectly regulating the expression of approximately one‐third of genes in Arabidopsis, with nearly 3,000 genes under its direct control (Lee et al., 2007; Yogev et al., 2020). Functioning downstream of multiple photoreceptors, HY5 is involved in seed germination, hypocotyl elongation, leaf expansion, flower organ development, metabolic regulation, and stress adaptation (Pedmale et al., 2016; Paik et al., 2017; Xu, 2019). Studies indicate that PIFs are essential in fine‐tuning plant responses to light conditions (Paik et al., 2017; Li et al., 2021a; Lan et al., 2022). In darkness, PIFs remain active, regulating the expression of particular genes that influence plant growth. In contrast, under light conditions, they become activated by photoreceptors, leading to their rapid degradation (Paik et al., 2017; Li et al., 2021a; Lan et al., 2022). While considerable advances have been made in understanding photomorphogenesis, our knowledge of how light modulates overall plant metabolism remains incomplete (Nieto et al., 2015; Paik et al., 2017; Park et al., 2018; Gil et al., 2020).

The tomato (*Solanum lycopersicum*), regarded as the world's most popular vegetable, serves as an essential model for plant metabolic research (Li et al., 2018, 2020c). As a typical climacteric fruit, changes in major metabolites within tomato fruit are closely linked with its growth and ripening processes (Li et al., 2018, 2020c; Chen et al., 2020). During the mature green stage, the biosynthesis of the plant hormone ethylene is significantly increased (Chen et al., 2020), which is followed by the induction of carotenoid and flavonoid biosynthesis, providing the primary pigments for ripe fruit (Li et al., 2020b). Additionally, the suppression of steroidal glycoalkaloid (SGA) biosynthesis eliminates toxic defensive compounds at the ripe stage (Lee et al., 2012; Itkin et al., 2013; Zhang et al., 2015; Li et al., 2018; Kazachkova et al., 2021; Shi et al., 2021; Ren et al., 2022; Yuan et al., 2022). The activation of genes involved in cell wall degradation further promotes the softening of fruit texture (Uluisik et al., 2016).

To date, numerous master regulators have been identified that closely control tomato fruit ripening and related metabolic changes. Transcription factors such as RIPENING‐INHIBITOR (SlRIN), NON‐RIPENING (SlNOR), SlCNR (Colorless Non‐Ripening), APETALA2a (SlAP2a), SlERF6, SlERF.F12, and EILs have been shown to regulate fruit ripening and ripening‐related quality traits in an ethylene‐dependent manner (Manning et al., 2006; Karlova et al., 2011; Lee et al., 2012; Zhang et al., 2015; Ito et al., 2017; Gao et al., 2020; Li et al., 2020b; Deng et al., 2022, 2023; Shi et al., 2022). Among these transcription factors, SlRIN, SlNOR, and SlCNR are well‐documented, with disruptions in these factors leading to impaired ripening. SlRIN encodes a MADS‐box transcription factor, whereas SlNOR and SlCNR encode a NAC transcription factor and a SQUAMOSA promoter binding protein‐like (SBP) transcription factor, respectively (Gao et al., 2019, 2020). SlRIN can directly bind to the promoters of *SlWRKY35* and *SlERF.G3‐Like*, activating their expression (Yuan et al., 2022; You et al., 2024). SlWRKY35 redirects primary metabolism toward the MEP pathway, thereby increasing carotenoid accumulation (Yuan et al., 2022). SlERF.G3‐Like regulates several genes involved in fruit ripening and the phenylpropanoid pathway (You et al., 2024). SlNOR increases fruit ripening by binding to the promoter of SlACS2 and activating its expression, which leads to ethylene production (Gao et al., 2019, 2020). Additionally, SlRIN, SlNOR, and SlCNR function upstream of SlAP2, influencing *β*‐carotene biosynthesis and fruit ripening (Karlova et al., 2011). These findings suggest that these key regulators coordinate with other transcription factors to form a complex regulatory network that controls ripening initiation and quality development (Chen et al., 2020; Li et al., 2020b; Liu et al., 2024). Although there has been extensive study of these ripening‐associated transcription factors, their interactions with epigenetic mechanisms and external environmental factors remain largely unexplored (Zhong et al., 2013; Lang et al., 2017; Lu et al., 2018).

Epigenetic regulation is essential in controlling plant growth, development, and responses to stress (Berr and Shen, 2010; Baulcombe and Dean, 2014; Lu et al., 2018; Crisp et al., 2022). In tomato fruit development and ripening, DNA demethylation significantly influences global gene expression (Berr and Shen, 2010; Zhong et al., 2013; Lang et al., 2017; Lu et al., 2018; Crisp et al., 2022). The fruit of the ripening‐deficient epimutant *cnr*, which exhibits DNA hypermethylation, fails to ripen properly and develops a colorless, mealy pericarp (Manning et al., 2006; Gao et al., 2019). During ripening, DNA methylation levels in promoter regions of genes involved in ethylene biosynthesis, carotenoid biosynthesis, flavonoid biosynthesis, and cell wall degradation significantly decrease (Lang et al., 2017). This DNA demethylation process enables access to binding motifs for key ripening regulators, such as SlRIN, thereby activating these genes during ripening (Zhong et al., 2013). This study highlights the role of SlDML2, a gene encoding a DNA demethylase in tomato, in the precise activation and repression of ripening‐related genes. Silencing or knocking out SlDML2 causes a marked increase in DNA methylation levels at the promoters of ripening‐associated genes, leading to pronounced delays in ripening and metabolic shifts within tomato fruits (Lang et al., 2017).

In contrast to genetic inheritance, epigenetic modifications are inherently flexible and can be significantly influenced by both biotic and abiotic factors (Baulcombe and Dean, 2014). Previous studies have shown that environmental conditions and biotic stressors can impact DNA methylation levels (Dowen et al., 2012; Baulcombe and Dean, 2014; Crisp et al., 2022; Sun et al., 2024). For instance, during the post‐harvest storage of tomatoes, cold treatment significantly reduces the expression of DNA demethylase *SlDML2*, resulting in promoter hypermethylation of genes involved in flavor volatile biosynthesis (Secco et al., 2015; Zhang et al., 2016; Bai et al., 2025). In rice, during inorganic phosphate (Pi) starvation, CHH hypermethylated regions appear, primarily overlapping with transposons near Pi‐starvation‐induced genes (Secco et al., 2015). Additionally, when Arabidopsis plants encounter the bacterial pathogen *Pseudomonas syringae* pv. *tomato* DC3000 (*Pst* DC3000), differential DNA methylation at gene boundaries contributes to varied gene expression in response to the pathogen (Dowen et al., 2012). Collectively, these findings suggest that biotic and environmental factors are key drivers in shaping plant epigenomes during critical biological processes. Nonetheless, the molecular mechanisms by which light signals directly influence the plant epigenome, especially in terms of whole‐genome DNA methylation, remain largely unknown.

In this study, we found that red or blue light supplementation in tomato fruit induced global metabolic changes. The Tomato Light‐induced Expression Database (TomLED) was established through comprehensive analyses that integrated metabolomic and transcriptomic data from tomato fruit samples collected at nine crucial developmental stages under various light‐quality treatments, alongside methylation patterns across four stages. Using TomLED, we observed that global metabolic and transcriptional changes are mediated by accelerated whole‐genome DNA demethylation through SlDML2. The key light signal regulator SlHY5 directly binds to the G‐box of *SlDML2*, activating its expression, thereby increasing DNA demethylation and facilitating metabolic changes during fruit ripening. This study uncovers the molecular mechanism by which light regulates the epigenome and establishes a direct link between light signals and plant metabolism.

## RESULTS

### Red or blue light supplements induce global metabolic and transcriptomic changes in tomato fruit

**Figure 1:**
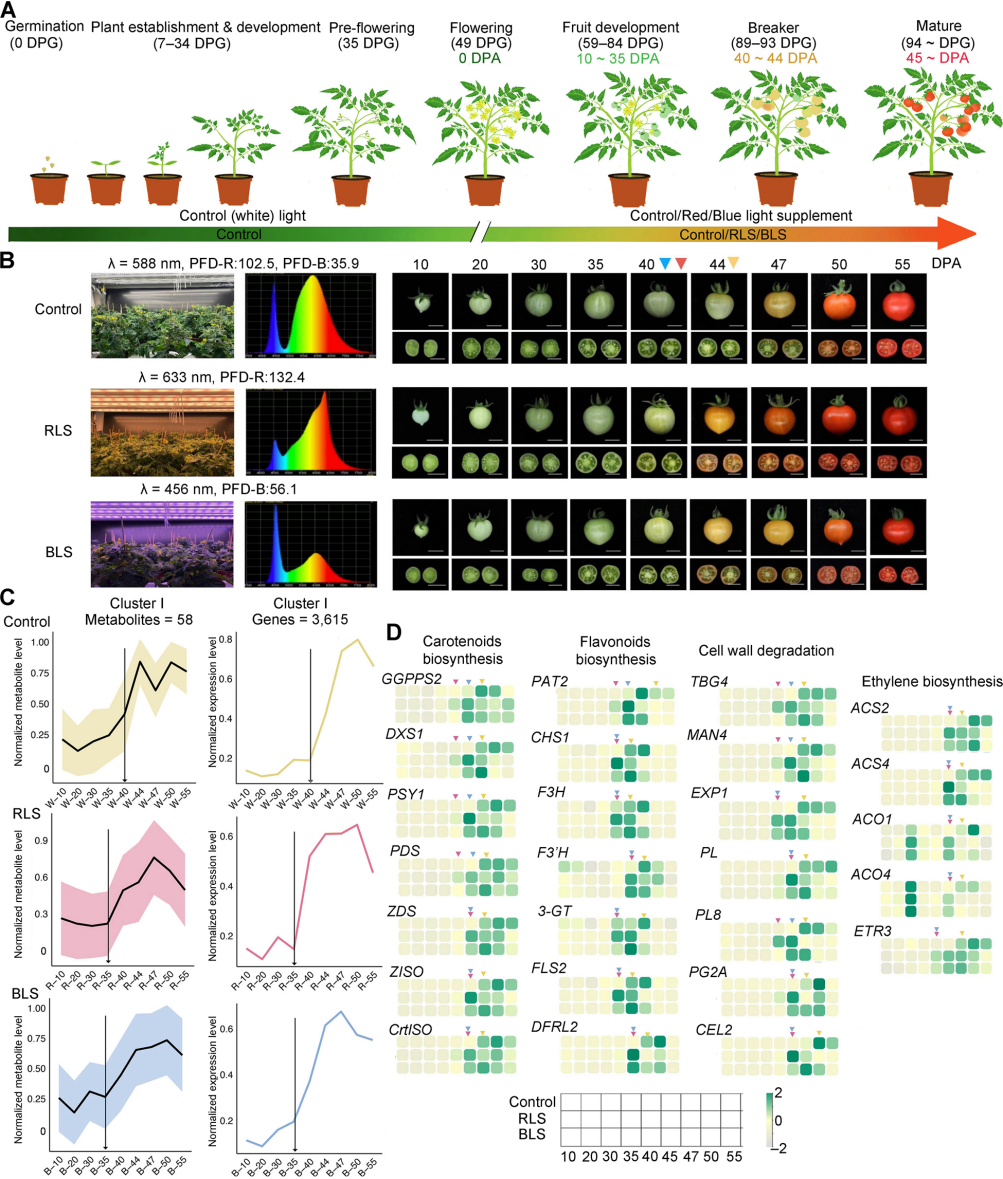
RLS or BLS induces substantial metabolic changes in tomato fruit **(A)** Schematic diagram of the design for the Tomato Light‐induced Expression Database (TomLED). Tomato seedlings were exposed to control (white light) before the flowering stage (0–48 d post‐germination (DPG)), while exposed to control, 30% RLS or 30% BLS at the flowering stage (0 or 49 d post‐anthesis (DPA)). Fruit samples were obtained from 10, 20, 30, 35, 40, 45, 47, 50, and 55 DPA. Bar = 1 cm. **(B)** Phenotypes of tomato fruit under three light conditions. The triangles indicate the breaker stage of tomato fruit. Yellow, red and blue color represent control light, RLS and BLS, respectively. All scale bars represent 1 cm. **(C)** *k*‐means clustering grouped the expression profiles of the tomato metabolome (left) and transcriptome (right). In Cluster I, both metabolites and genes were induced earlier in the red/blue light treatment group compared to the control group. Clusters II–X are shown in Figure S2. **(D)** Heatmap showing RLS or BLS advanced the expression of secondary metabolic and fruit ripening genes in Cluster I. Triangles indicate the time when gene expression was induced. Yellow, red and blue color was used to represent control, RLS and BLS, respectively. *Z*‐scores of data sets were standardized to 2 to −2. The green represents 2, while gray represents −2.

Light quality, defined by wavelength, plays a key role in the metabolic regulation of plants (Secco et al., 2015; Wang and Lin, 2020; Gao et al., 2022b; Xu et al., 2024). In modern agricultural practices, optimizing light quality is essential for maximizing light energy absorption and utilization (Wang and Lin, 2020; Pierik and Ballaré, 2022). To investigate whether light quality significantly affects tomato fruit metabolism, we conducted growth experiments under various lighting conditions (Figure 1A). All seedlings were initially grown under control conditions with a photosynthetic photon flux density (PPFD) of 250 ± 10 μmol/m^2^/s at the top of the seedlings and a peak light wavelength of 588 nm. Upon reaching the flowering stage (48 d post‐germination, 48 DPG), the plants were divided into three groups with distinct light treatments. One group remained under the original control light, while the other two groups were exposed to supplementary 30% red light (peak wavelength at 633 nm) and 30% blue light (peak wavelength at 456 nm), respectively. The PPFD for all three groups was maintained at 250 ± 10 μmol/m^2^/s (Figure 1A, B).

As shown in Figure 1B, both red light supplementation (RLS) and blue light supplementation (BLS) led to substantial phenotypic changes in the tomato fruit (Figure 1B). Red light supplementation slightly increased both the equatorial diameter and weight of the tomato fruit, whereas BLS caused a decrease in these metrics (Figure S1A). The initiation of ripening in fruit treated with RLS or BLS was observed at 40 d post‐anthesis (DPA), with noticeable color change beginning at 44 DPA. By 49 DPA, fruit under RLS displayed a red hue, while fruit under control and BLS conditions showed a yellow hue. Additionally, the color of mature fruit under control light appeared lighter compared to fruit under RLS and BLS (Figure 1B). At 55 DPA, fruit in all light conditions reached full ripeness, although fruit under control light remained a lighter color than those under RLS and BLS.

To assess their influence on fruit color, the primary carotenoids and total flavonoids were analyzed. The total levels of *α*‐carotene, *β*‐carotene, and lycopene were significantly higher in fruit grown under BLS or RLS compared to control light at 40, 44, and 47 DPA (Figure S1B). Interestingly, lycopene accumulation was induced in 40 DPA fruit under RLS, in 44 DPA fruit under BLS, and in 47 DPA fruit under control light. Total flavonoid content was also elevated in fruit treated with BLS at 40, 44, and 47 DPA, and in fruit treated with RLS at 44 and 47 DPA (Figure S1B). Notably, the elevated flavonoid content under blue light conditions induces a more pronounced yellowish hue in the fruit, thereby reducing its redness relative to that observed under red light conditions (Figures 1B, S1B) (He et al., 2022; Liu et al., 2022). These findings indicate that different light qualities substantially impact tomato fruit metabolism.

To further examine the light‐induced global metabolic and transcriptomic changes in tomato fruit under the three light treatments, tomato pericarps were collected at nine developmental stages: 10, 20, 30, 35, 40, 44, 47, 50, and 55 DPA (Figure 1B), and were analyzed for metabolome and transcriptome profiles. For metabolome analysis, samples underwent non‐targeted liquid chromatography‐tandem mass spectrometry (LC‐MS/MS) and gas chromatography (GC)‐MS‐based profiling, identifying a total of 479 metabolites (Table S1). Transcriptome analysis yielded ∼960 Gb of raw data and 928 Gb of clean RNA sequencing (RNA‐seq) data, with sequencing library statistics summarized in Table S2. Uniquely mapped reads were used to calculate expression levels in transcripts per million (TPM) (Table S3). This comprehensive data set was designated as the TomLED.

Analysis of TomLED revealed distinct expression patterns of major fruit‐enriched metabolites under different light treatments. For metabolites prominent at early fruit developmental stages, such as tomatidine, expression patterns remained consistent across the light conditions (Figure S1C). However, compounds primarily produced during ripening stages displayed substantial shifts in expression patterns under BLS and RLS. For instance, the synthesis of carotenoids like *α*‐carotene, *β*‐carotene, and lycopene was initiated earlier with RLS or BLS (Figure S1C). A similar trend was observed for metabolites in the phenylpropanoid pathway, including benzoic acid, ferulic acid, naringenin chalcone, naringenin, and kaempferol‐3‐*O*‐galactoside (Figure S1C).

To further access the light‐induced metabolic changes, we first analyzed the metabolites under control light by dividing all 479 annotated metabolites into 10 clusters based on their accumulation patterns using the *k*‐means clustering algorithm (Figures 1C, S2A, B). For each cluster, we then analyzed the expression of the same metabolites in RLS/BLS conditions. Interestingly, we found the expression patterns of compounds in Cluster I, which were highly expressed in the ripening stages under control light, were substantially advanced under RLS and BLS (Figure 1C).

Next, we conducted a co‐expression analysis on our metabolome and transcriptome data for the control group. To ensure rigor, we applied a stringent multiple test correction (*r* ≥ 0.8) to filter out genes that were significantly correlated with each metabolite. In total, 24,464 genes were grouped into 10 clusters. For each cluster, we then analyzed the expression of these same genes in RLS/BLS conditions (Figures 1C, S2C, D). Similar to the metabolomic data, we found that in Cluster I, the induction of ripening‐related genes was substantially induced earlier in the RLS and BLS (Figure 1C). Gene Ontology (GO) enrichment and Kyoto Encyclopedia of Genes and Genome (KEGG) analysis indicate these genes are involved in ripening‐related genes such as carotenoid biosynthesis, phenylpropanoid pathway and cell wall modification (Figure S3).

We further analyzed the expression of individual genes in Cluster I. The expression of key biosynthetic genes involved in carotenoid synthesis (*SlGGPPS2*, *SlDXS1*, *SlPSY1*, *SlPDS*, *SlZDS*, *SlZISO*, and *SlCrtISO*) and phenylpropanoid synthesis (*SlCHS1*, *SlF3H*, *SlF’3H*, *Sl3‐GT*, *SlFLS2*, and *SlDFRL2*) was induced earlier under RLS and BLS (Figure 1D). This early gene expression aligns closely with the accelerated accumulation of carotenoids and phenylpropanoids observed under these light treatments (Figure 1D). Interestingly, we also noted that genes associated with ethylene production (*SlACS2*, *SlACS4*, *SlACO1*, *SlACO4*, *SlETR3*) and cell wall degradation (*SlTBG4*, *SlMAN4*, *SlEXP*, *SlPL*, *SlPL8*, *SlPG2A*, *SlCEL2*) followed a similar expression pattern (Figure 1D). These genes play crucial roles in the ripening process of tomato fruit, suggesting that the accumulation of key metabolites and the overall ripening process were notably advanced under RLS and BLS.

### Light receptors SlphyB2 and SlCRY1a are involved in RLS or BLS‐enhanced metabolic and ripening changes

Previous studies suggest that light signals are initially detected and transmitted by plant receptors (Jing and Lin, 2020). To explore whether specific red or blue light receptors are involved in RLS or BLS‐driven metabolic and ripening changes, we examined the expression patterns of four primary red/blue light sensors in tomato (Figure S4) (Alba et al., 2000a; 2000b; Franklin et al., 2003; Chaves et al., 2011; Facella et al., 2012; Lopez et al., 2012; Pham et al., 2018). Our findings showed that *SlPHYB1* was highly expressed in the stem and did not respond to RLS in fruit (Figure S4A–C). In contrast, *SlPHYB2* expression increased at the breaker stage and responded to RLS treatment (Figure S4D–F). For blue light, *SlCRY1a* exhibited high expression during fruit ripening and responded to BLS (Figure S4G–I), whereas *SlCRY1b* showed low expression in tomato fruit and did not respond to BLS (Figure S4J–L). These results suggest that SlphyB2 and SlCRY1a may play key roles in regulating fruit metabolism under RLS and BLS, respectively.

**Figure 2:**
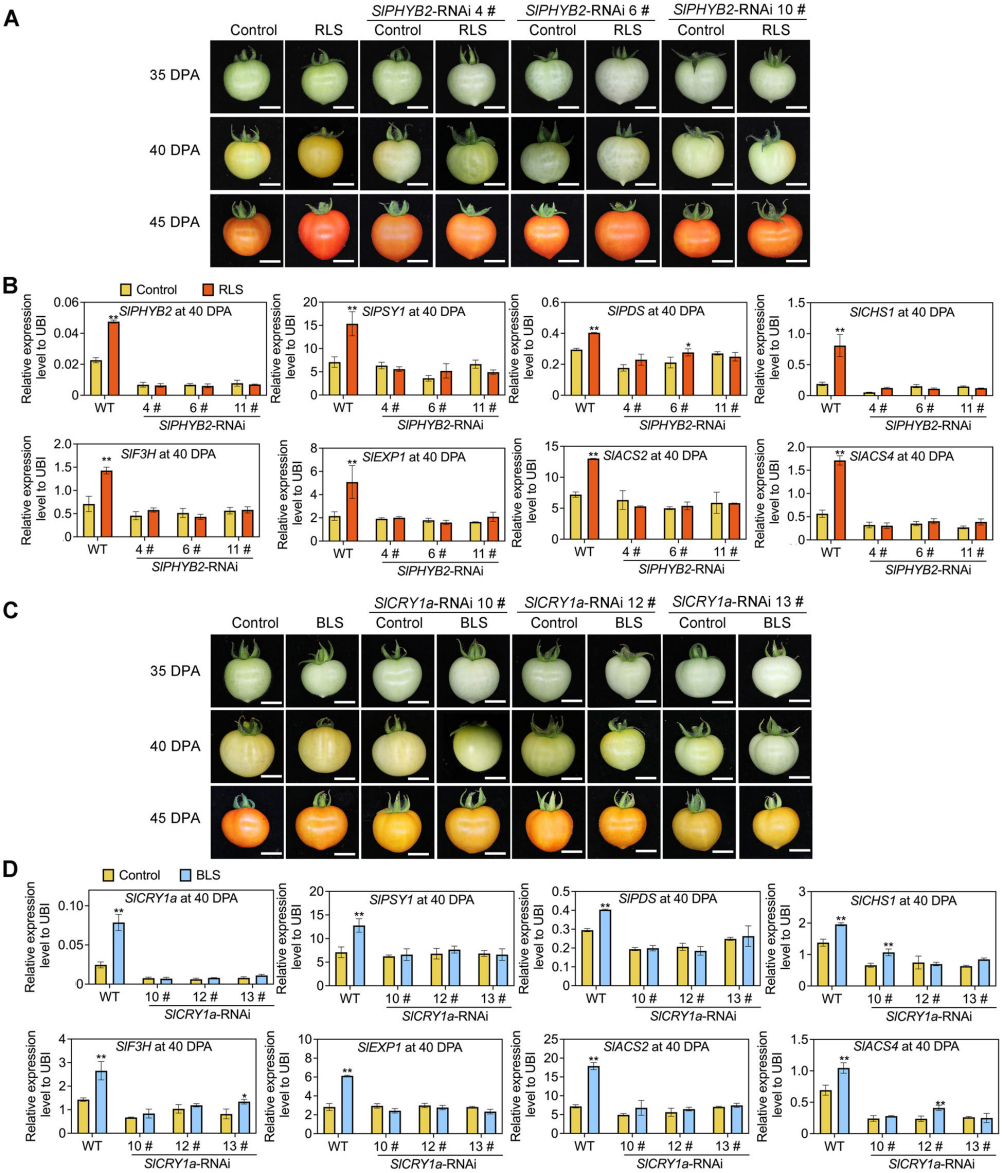
Light receptors SlPHYB2 and SlCRY1a are involved in red and blue light supplement‐accelerated fruit ripening, respectively **(A**, **B)** Phenotypes (at 35, 40, and 45 d post‐anthesis (DPA)) **(A)** and relative expression levels (at 40 DPA) of key metabolic and ripening‐related genes **(B)** of *SlPHYB2*‐RNAi (RNA interference) fruits supplemented with red light. **(C**, **D)** Phenotypes (at 35, 40, and 45 DPA) **(C)** and relative expression levels (at 40 DPA) of key metabolic and ripening‐related genes **(D)** of *SlCRY1a*‐RNAi fruits supplemented with blue light. All scale bars represent 1 cm. *SlUBI* was used as the internal control, **P < 0.01 and *P < 0.05 indicate significant differences in the same genotypes of tomato fruit compared to the control condition (Student's *t*‐test, *n* = 3).

To further assess the roles of these light receptors, we generated *SlPHYB2*‐RNAi (RNA interference) and *SlCRY1a*‐RNAi plants with decreased expression of *SlPHYB2* and *SlCRY1a*, respectively (Figure 2A, C). The RNAi constructs were designed to target unique regions of *SlPHYB2* and *SlCRY1a* to ensure gene‐specific knockdown (Figure S5). In *SlPHYB2*‐RNAi fruit, the response to RLS was significantly reduced compared to wild type (WT), and similarly, the response of *SlCRY1a*‐RNAi fruit to BLS was also reduced (Figure 2A, C). Additionally, the expression of key metabolic and ripening genes was no longer enhanced by RLS or BLS in *SlPHYB2*‐RNAi and *SlCRY1a*‐RNAi fruits, respectively (Figure 2B, D). These findings indicate that SlphyB2 and SlCRY1a specifically mediate the RLS‐ and BLS‐driven metabolic and ripening changes in tomato fruit.

### Major transcription factors are highly co‐expressed with ripening‐associated genes

**Figure 3:**
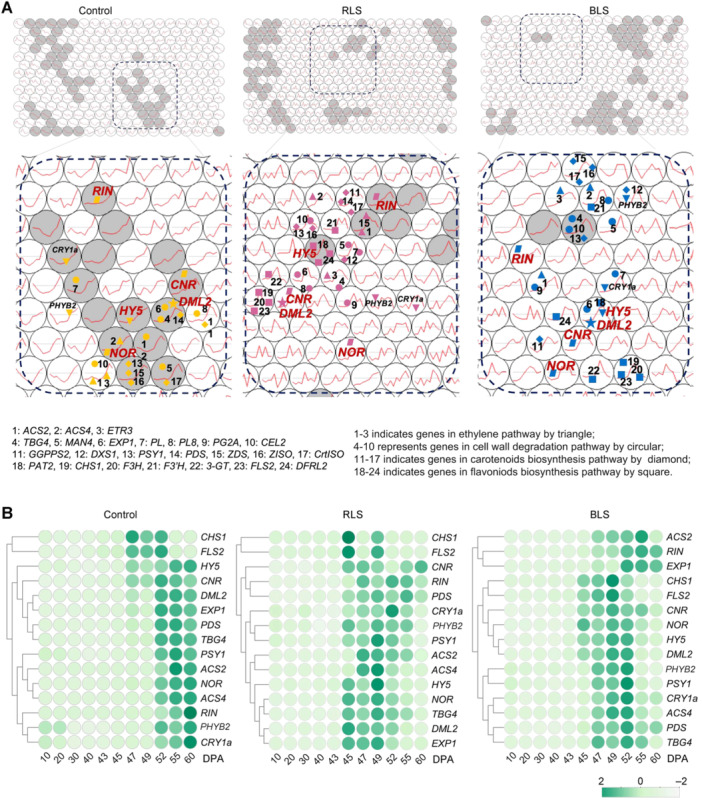
Self‐organizing map of TomLED data **(A)** Self‐organizing map of TomLED data. Each circular node represents unique genes with the most similar expression profile, and the neighboring nodes are decided by the similarity of their expression profile. In addition, there is one mega‐cluster represented by deep blue squares containing nodes that were spatially close and of high quality. Genes involved in carotenoid biosynthesis, phenylpropanoids pathway, cell wall degradation, and ethylene pathway are labeled by numbers. Gray nodes indicate the nodes with high quality. Each circular node represents unique genes with the most similar expression profile, and the neighboring nodes are decided by the similarity of their expression profile. In addition, there is one mega‐cluster represented by deep blue squares containing nodes that were spatially close and of high quality. Genes involved in carotenoid biosynthesis, phenylpropanoids pathway, cell wall degradation, and ethylene pathway are labeled by numbers. Gray nodes indicate the nodes with high quality. **(B)** Heatmap of co‐expression genes in the self‐organizing map. *Z*‐scores of data sets were standardized to 2 to −2. The green represents 2, while gray represents −2.

We initially grouped all 479 metabolites into 10 clusters based on their accumulation patterns using the *k*‐means method in R (Figures 1C, S2B; Table S4). To identify genes significantly correlated with at least one metabolite, a stringent multiple test correction (*r* ≥ 0.8) was applied, and these genes were similarly divided into 10 clusters (Figures 1C, S2D; Table S4). The overall ripening process and accumulation of ripening‐related metabolites appeared to be notably induced under RLS and BLS conditions. To examine this further, we conducted a self‐organizing map (SOM) analysis of all gene expressions under the three light treatments (Figure 3). Self‐organizing map clusters genes with similar expression profiles into nodes using an unsupervised neural network, providing insights into gene expression patterns and regulation (Wang et al., 2022c). We found that genes involved in carotenoid biosynthesis, phenylpropanoid biosynthesis, cell wall degradation, ethylene biosynthesis and signaling, and ripening‐associated transcription factors (TFs) exhibited strong co‐expression across all three light conditions (Figures 3A, S6A; Table S5). Interestingly, the expression of key master regulators, *SlRIN*, *SlNOR*, and *SlCNR*, was closely associated with these metabolic pathways under each light condition (Figures 3B, S6A). Further co‐expression analysis of *SlRIN*, *SlNOR*, and *SlCNR* with metabolite levels revealed that these ripening‐related TFs consistently exhibited high co‐expression with numerous metabolites. For instance, *SlRIN* was co‐expressed with 56, 18, and 27 metabolites under control, RLS, and BLS conditions, respectively. Notably, *β*‐carotene was co‐expressed with *SlRIN* in both RLS and BLS conditions (Figure S6B; Table S6). This suggests that *SlRIN* expression is closely linked to ripening‐associated genes and metabolites across different light treatments.

Additionally, *SlRIN*, *SlNOR*, and *SlCNR* expression were markedly promoted under RLS or BLS (Figure 1D), and this light‐induced expression was reduced when *SlPHYB2* or *SlCRY1a* were silenced (Figure S6C, D).

### SlDML2‐regulated DNA demethylation is involved in light‐induced metabolic and ripening changes in tomato fruit

**Figure 4:**
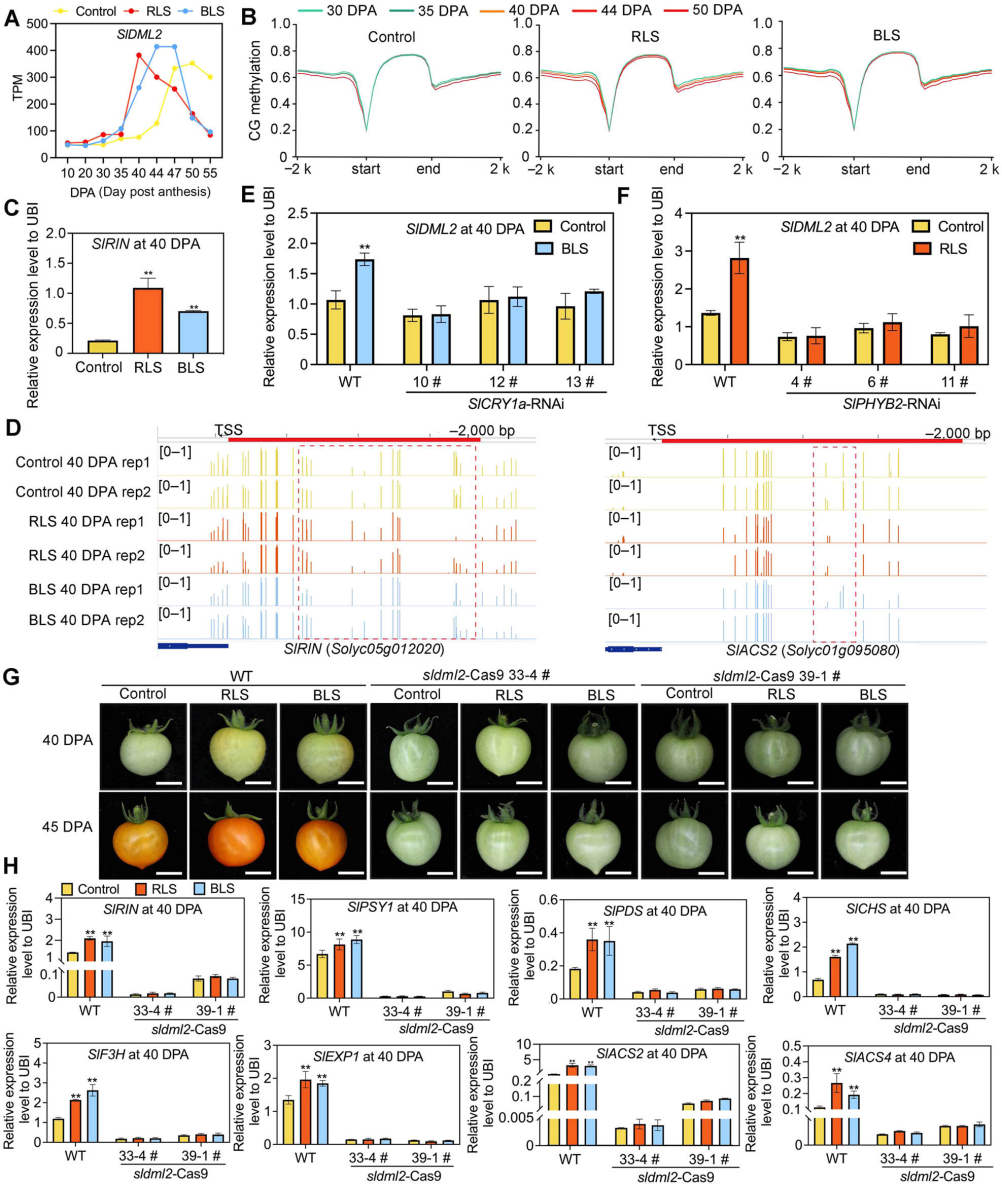
SlDML2‐regulated DNA methylation was involved in light‐induced metabolic and ripening changes of tomato fruit **(A**) The expression level of *SlDML2* in tomato fruits under three light conditions in the Tomato Light‐induced Expression Database (TomLED). **(B)** Metaplots of CG methylation level over genes (*n* = 34,075) and flanking 2,000 bp regions in wild‐type (WT) fruits under three light conditions during fruit development and ripening. **(C)** Relative expression of *SlRIN* at 40 d post‐anthesis (DPA). **(D)** DNA methylation levels (mCG contents) of 2,000 bp upstream promoter regions of *SlRIN* and *SlACS2* are shown with screenshots of Integrative Genomics Viewer (IGV) display of whole‐genome bisulfite sequencing data. **(E**, **F)** The expression level of *SlDML2* in *SlPHYB2*‐RNAi (RNA interference) fruits under red light supplementation (RLS) **(E)** and *SlCRY1a*‐RNAi fruits under blue light supplementation (BLS) **(F)** at 40 DPA. **(G)** Phenotypes of *sldml2*‐Cas9 fruits under three light conditions at 40 and 45 DPA. Bar represents 1 cm. **(H)** Expression levels of ripening‐associated genes and transcription factors (TFs) in tomato fruit at 40 DPA. *SlUBI* was used as the internal control, **P < 0.01 and *P < 0.05 indicate significant differences compared to the control condition (Student's *t*‐test, *n* = 3).

Previous studies have demonstrated that epigenetic regulation, particularly DNA methylation, plays a crucial role in controlling the timing of tomato fruit ripening (Zhong et al., 2013; Lang et al., 2017). During fruit development, promoter regions of key ripening‐related genes, such as *SlRIN*, undergo gradual demethylation, which promotes their expression (Zhong et al., 2013). Analysis of the SOM data (Figure 3A) revealed that the expression of *SlDML2*, a major DNA demethylation gene in tomato, shows a high correlation (*r* > 0.75) with *SlRIN*, *SlNOR*, *SlCNR*, and other key metabolic and ripening‐related genes (Figure 3A, B). Expression of *SlDML2* increases during ripening stages and is upregulated by RLS and BLS treatments (Figure 4A). Whole genome bisulfite sequencing (WGBS) analysis indicated substantial changes in DNA methylation levels across gene regions under RLS and BLS. Compared to control conditions, CG and CHG methylation levels in gene promoter regions were decreased under RLS and BLS (Figures 4B, S7A, B). Comparing CG methylation alterations of RLS/BLS and control at the same developmental stage, we found minimal differences existed between RLS/BLS and control in the early stages (30 DPA). However, during the fruit maturation and ripening process, the decreasing of methylation level in the RLS/BLS treatment was accelerated compared to that in the control group, resulting in lower methylation levels in the RLS/BLS fruit (Figure S7C).

We then checked the promoter methylation levels (mCG and mCHG) for genes involved in carotenoid biosynthesis (*SlMAN4*), phenylpropanoid biosynthesis (*SlF3H*), cell wall degradation (*SlEXP1*), ethylene production (*SlACS2*), and key regulator *SlRIN*. We found that the decreasing CG and CHG methylation levels in their promoter regions were markedly faster under RLS or BLS, corresponding to their increased expression levels (Figures 4C, D, S8–11). On the other hand, no obvious changes were observed in promoters of these genes in the CHH methylation context (Figure S12). Similar to *SlRIN*, the RLS‐ and BLS‐induced expression of *SlDML2* was abolished in *SlPHYB2* or *SlCRY1a* plants (Figure 4E, F), indicating the induction of *SlDML2* requires the activation of photoreceptors.

The ePlant and MMN databases indicate that *SlDML2* is expressed across nearly all plant tissues, with particularly high expression levels during the fruit's color‐breaking stage (Figure S13) (Waese et al., 2017; Li et al., 2020c). To clarify the role of SlDML2 in light‐mediated metabolic and ripening changes, we generated stable *sldml2‐*Cas9 lines. Using two guide RNAs (gRNAs), we obtained two lines with 1‐ and 13 bp deletions, both of which are expected to introduce premature stop codons (Figure S14A, B). Up to the flowering stage, no substantial phenotypic differences were observed between the *sldml2‐*Cas9 lines and WT plants (Figure S14C). However, when WT fruits turned red under all three light conditions, the *sldml2‐*Cas9 fruits retained a green phenotype (Figure 4G). Additionally, the RLS‐ and BLS‐induced expression of *SlRIN* and genes involved in secondary metabolism, the ethylene pathway, and cell wall degradation were abolished in *sldml2‐*Cas9 mutants (Figure 4H). These findings indicate that RLS‐ and BLS‐induced metabolic and ripening changes in tomato fruit are mediated by SlDML2‐regulated whole genome DNA demethylation.

### SlHY5 directly enhances the expression of SlDML2 to connect light signals with plant metabolism global DNA demethylation

**Figure 5:**
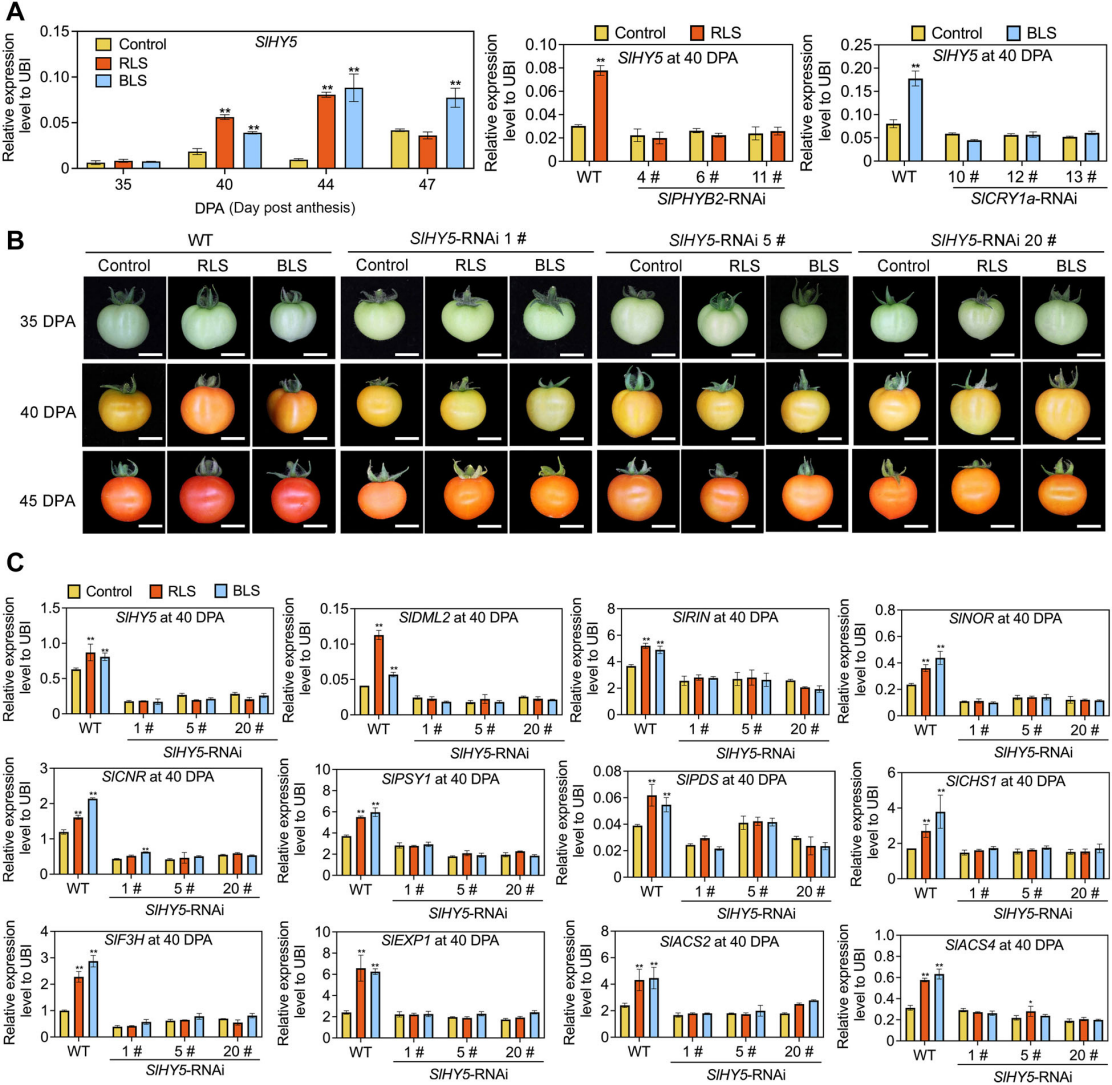
SlHY5 plays important roles in the ripening of tomato fruit **(A)** The expression levels of *SlHY5* at 35, 40, 44, and 47 d post‐anthesis (DPA) verified by quantitative polymerase chain reaction with reverse transcription (RT‐qPCR). **(B)** The expression level of *SlHY5* in *SlPHYB2*‐RNAi (RNA interference) fruits under red light supplementation (RLS) and *SlCRY1a*‐RNAi fruits under blue light supplementation (BLS), respectively. **(C)** Phenotypes of *SlHY5*‐RNAi fruits under Control, RLS and BLS. Bar represents 1 cm. **(D)** The expression level of *SlHY5* and ripening‐associated genes in *SlHY5*‐RNAi fruits at 40 DPA. *SlUBI* was used as the internal control, **P < 0.01 and *P < 0.05 indicate significant differences between RLS/BLS and control in the same genotypes of tomato fruit (Student's *t*‐test, *n* = 3).

To further investigate, we conducted a yeast one‐hybrid screening using *proSlDML2* as bait. In total, we identified a total of 229 TFs (Figure S15A; Table S7). Additional co‐expression analysis with *SlDML2* as the target revealed 12 TFs, and among these, six genes (*Solyc09g075440*, *Solyc07g052700*, *Solyc07g052960*, *Solyc07g055920*, *Solyc12g087830*, and *Solyc08g061130*) were selected out by both means (Figure S15A, B). Among these six genes, we noticed the expression of *Solyc08g061130*, which annotated as SlHY5, a key light signal regulator (Delker et al., 2014; Wang et al., 2018, 2025; Xu, 2019; Lee et al., 2021; Li et al., 2021a; Zhang et al., 2022a), was significantly upregulated by RLS or BLS (Figure 5A). However, in *SlPHYB2*‐RNAi or *SlCRY1a*‐RNAi fruits, *SlHY5* no longer responded to RLS or BLS (Figure 5B). In WT fruits, *SlHY5* expression and fruit ripening were markedly elevated by RLS and BLS, and the induction of ripening was absent in *SlHY5*‐RNAi lines (Figures S16, 5C, D). In *SlHY5*‐RNAi fruits, the expression of *SlDML2*, *SlRIN*, and key metabolic and ripening genes was significantly reduced under control light conditions and showed no responsiveness to RLS or BLS (Figure 5D). Interestingly, while *SlHY5* expression was readily induced by RLS and BLS in WT (Figure 5A), this induction was absent in *sldml2‐*Cas9 fruits (Figure S15C), likely due to the demethylation of the *proSlHY5* promoter during ripening (Figures S15D, S17). Interestingly, analysis of the SOM data showed that the expression of the key light signal gene, *SlHY5*, is strongly correlated with *SlDML2*, *SlRIN*, *SlNOR*, *SlCNR*, and other metabolic and ripening‐related genes across all light conditions (Figure 3). Collectively, these results indicate that although SlHY5 acts upstream of SlDML2 to link light signals with DNA demethylation, its expression can also be further induced by promoter demethylation during ripening.

**Figure 6:**
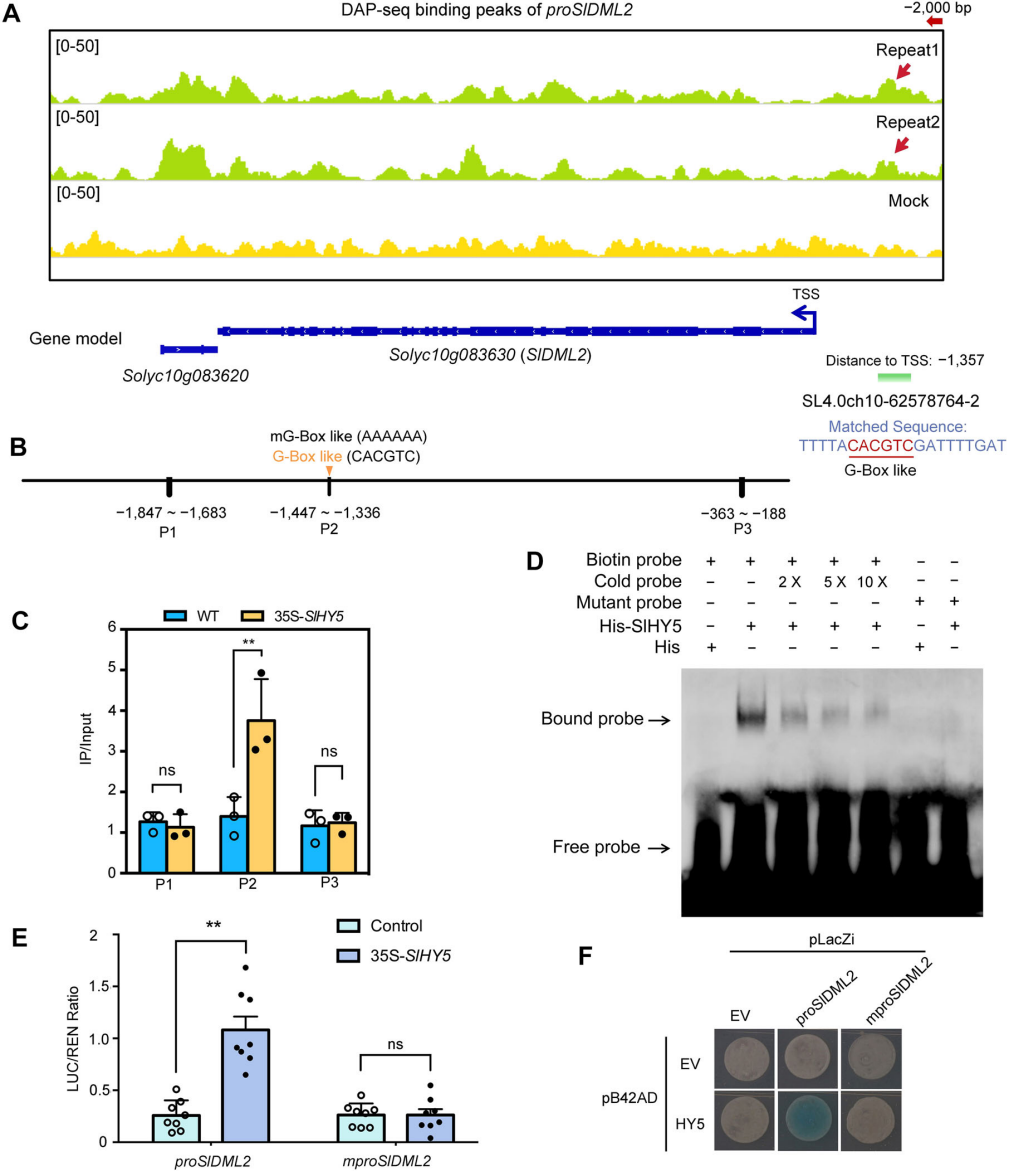
**SlHY5 directly induces the expression of** ***SlDML2*** **to determine the light‐mediated fruit metabolic changes** **(A)** SlHY5 DNA‐affinity purification sequencing (DAP‐seq) binding peaks of *proSlDML2*. Peaks in green represent two replicates, while yellow color represents mock (input). **(B)** Schematic representation of the design for the chromatin immunoprecipitation‐quantitative polymerase chain reaction (ChIP‐qPCR), dual‐luciferase (LUC) and yeast one‐hybrid (Y1H). The p1, p2 and p3 indicates −1,847 ~ −1,683 bp, −144 ~ −1,336 bp, −363 ~ −188 bp sequences from the upstream of TSS (transcription start sites), respectively. The position of G‐Box is indicated by a yellow triangle. **(C)** Chromatin immunoprecipitation analysis of SlHY5 binding to the regions of *SlDML2* in the wild type (WT) and transgenic lines of *35S‐SlHY5*. −1,847 ~ −1,683 bp (P1) or −363 ~ −188 bp (P3) act as a negative control. This experiment was repeated independently three times with similar results. ns indicates not significant (*P* > 0.05). *P < 0.05 Indicates significant difference from WT analyzed by two‐sided Student's *t*‐test (*n* = 3). **(D)** Electrophoretic mobility shift assay (EMSA) of SlHY5 binding to the P2/mP2 fragment. SlHY5 binds to the P2 fragment (biotin probe) of *proSlDML2*, while the mutant of P2 (mP2, mutant probe) does not present binding. “+” indicates presence; and “−“ indicates absence. **(E)** Dual‐LUC reporter assay validated the activation of the *SlDML2* promotor by SlHY5. The LUC/REN (Renilla) ratio represents the average ratio of the bioluminescence of firefly LUC to that of Renilla LUC. ns indicates not significant (*P* > 0.05), **P < 0.01 indicates the significant difference (Student's *t*‐test, *n* = 8). **(F)** Yeast one‐hybrid assay showing SlHY5 binding to the *SlDML2* promotor (*proSlDML2*), while not binding to the mutant of P2 (*mproSlDML2*). *mproSlDML2* is a 2,000 bp promoter sequence with a mutation in the P2 domain (G‐Box).

We then conducted DNA‐affinity purification sequencing (DAP‐Seq) for SlHY5 and found it specifically binds to the G‐Box motif in the *proSlDML2* (Figure 6A; Table S9). Additional assays, including chromatin immunoprecipitation‐quantitative polymerase chain reaction (ChIP‐qPCR), transient dual‐luciferase reporter assay (Dual‐LUC), electrophoretic mobility shift assay (EMSA), and yeast one‐hybrid (Y1H), further confirmed that SlHY5 binds to the G‐Box like motif within the *SlDML2* promoter region. And the induction of SlHY5 to *proSlDML2* was abolished when the G‐Box was mutated. All these data indicate SlHY5 activates the expression of *SlDML2* by directly binding to the G‐Box of its *proSlDML2* (Figure 6B–F).

To further examine the impact of SlHY5 on SlDML2 and global DNA methylation, we generated *slhy5* knockout mutants using clustered regularly interspaced short palindromic repeats (CRISPR)/CRISPR‐associated protein 9 (Cas9), obtaining two lines with 1 and 43 bp deletions, respectively (Figure S18A). Compared to WT, both *slhy5* knockout (KO) lines displayed delayed ripening under standard growth conditions (Figure S18B). *SlDML2* induction during fruit development was delayed in these mutants (Figure S18C), resulting in a substantial delay in the reduction of global CG and CHG methylation levels in gene promoter regions (Figure S18D–I). Similarly, the demethylation of promoters for key RLS‐ and BLS‐induced genes was also delayed (Figure S19). Collectively, these results indicate that SlHY5 directly activates *SlDML2* by binding to the G‐Box like in its promoter, thereby accelerating the reduction of DNA methylation in promoter regions of key ripening and metabolic genes.

**Figure 7:**
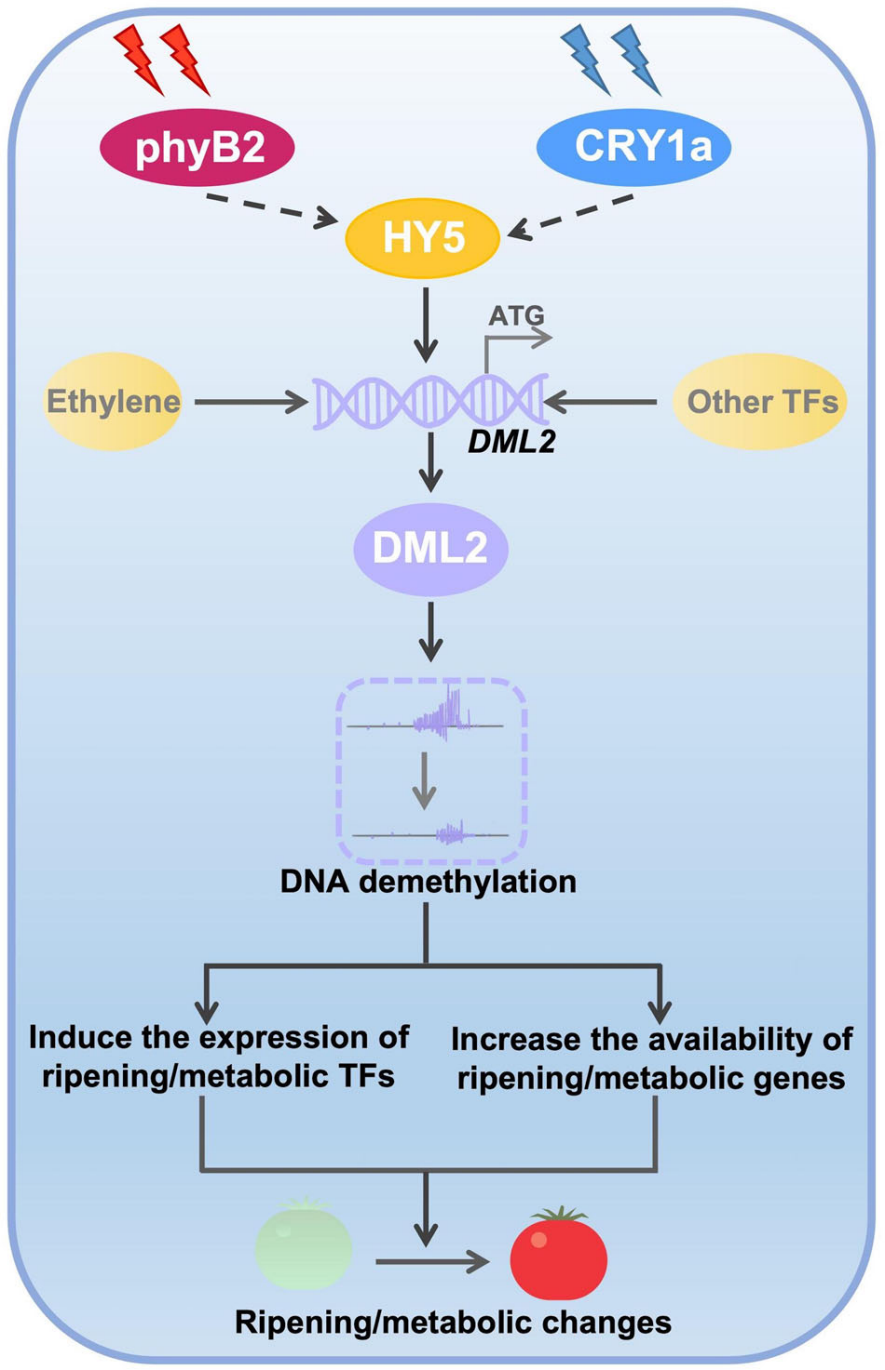
The working model of red/blue light‐mediated tomato metabolic and ripening changes Under red or blue light, the light‐activated SlphyB2 or SlCRY1a positively regulates SlHY5. SlHY5 then activates the expression of *SlDML2*, and consequently promoting DNA demethylation. The whole genome DNA demethylation enhances the expression of key ripening and metabolic transcription factors (TFs) (e.g., *RIN*, *NOR*, *CNR*, *HY5*) and metabolic/ripening genes to notably change the metabolism and ripening of tomato fruit.

In summary, we propose a model for RLS‐ and BLS‐induced metabolic and ripening changes in tomato: upon receiving external red or blue light signals, the red‐light receptor SlphyB2 or blue light receptor SlCRY1a activates transcription of the light signaling component *SlHY5*. SlHY5 binds to the G‐Box like in the *SlDML2* promoter, increasing its transcription and leading to global DNA demethylation in tomato fruit. Consequently, the induction of key TFs and other metabolic/ripening genes is advanced due to reduced promoter DNA methylation levels, thereby accelerating metabolic changes and fruit ripening under RLS or BLS (Figure 7).

## DISCUSSION

As sessile organisms, plants generate a variety of specialized metabolites in response to external stimuli and changing environmental conditions (Zhang et al., 2022c). Beyond their essential roles in plant growth and stress response, these natural compounds influence taste, color, flavor, and the nutritional profile of plant‐based foods (Li et al., 2020c). Understanding how environmental factors impact plant metabolism is therefore crucial for designing health‐promoting foods and optimizing production systems (Scheres and van der Putten, 2017; Qiu et al., 2021). Among these environmental factors, light acts not only as a primary energy source but also as an essential regulatory signal that affects growth, development, and stress responses (Zhang et al., 2022c, 2023). Compared to advances in plant photomorphogenesis research, studies on the regulation of plant metabolism by light are still relatively limited. Light quality, defined by wavelength, is a critical aspect of the light signal that influences plant growth and development, making it a valuable tool in agricultural practice (Lin et al., 1995; Vitale et al., 2022; Zhang et al., 2022c). By sensing and adapting to specific light qualities, plants can optimize growth, development, and yield, as well as improve product quality. Supplemental red and blue light induce metabolic changes in fruit primarily through the action of specific photoreceptors, a well‐documented mechanism (Rosado et al., 2019; Li et al., 2021b; Zhang et al., 2022c; Wang et al., 2025). Functioning as master switches, these receptors orchestrate complex signaling cascades that culminate in the rewiring of gene expression and metabolic pathways (Rosado et al., 2019; Wang et al., 2021). The responses of various metabolites to light spectrum exhibit notable specificity (Gil et al., 2020; Li et al., 2021b, 2021c; He et al., 2022). In this study, blue light promotes the synthesis of flavonoids more effectively than red light (Figure S1B), despite their comparable photosynthetic efficiencies. This distinct response is attributed primarily to the signaling roles of CRYs and PHYs, rather than being an indirect consequence of enhanced photosynthesis. Photoreceptors effectively activate metabolic pathways even at light levels below the photosynthetic saturation point (Jiao et al., 2007; Galvão and Fankhauser, 2015). While the fundamental role of photosynthesis as the primary energy source must be acknowledged, it operates in concert with, rather than in opposition to, light receptor signaling pathways. The ripening process was significantly delayed in photoreceptor RNAi plants exposed to RLS/BLS (Figure 2A), accompanied by a failure to upregulate key ripening‐related genes (Figure 2B). This impairment offers compelling evidence that photoreceptors are central mediators of light‐induced ripening. While previous studies have examined the effects of red and blue light on photosynthesis in leaves and fruit ripening in several species, most of this research has focused on the interaction between light signals and specific pathways or genes (Li et al., 2021c; Liu et al., 2022). However, a comprehensive analysis of how light quality influences global metabolism and the underlying mechanisms remains underexplored, limiting the development of innovative breeding materials.

As one of the world's most popular fruits, tomato serves as an ideal model for studying plant metabolic regulation (Li et al., 2018). During tomato fruit development, large‐scale metabolic changes are intricately regulated by the ripening process. Two main factors have been identified in controlling tomato fruit ripening and metabolism: Key master regulators and epigenetic modifications. Studies have shown that whole‐genome DNA demethylation is crucial during tomato development to activate the expression of essential genes for ripening, metabolism, and pathogen resistance (Zhong et al., 2013; Ruie et al., 2015; Lang et al., 2017; Li et al., 2020d; Ding et al., 2021; Jiang et al., 2021). Furthermore, DNA methylation levels in tomato plants can dynamically adjust in response to environmental factors, including light, temperature, and humidity (Zhong et al., 2013; Ruie et al., 2015; Zhang et al., 2018). Biotic stresses, such as pathogen infections or insect attacks, can also trigger genome‐wide DNA methylation changes, influencing the expression of genes related to disease and insect resistance, which in turn affects the plant's defense and survival mechanisms (Zhang et al., 2022a). A comprehensive understanding of DNA demethylation mechanisms is therefore essential for improving tomato yield and quality, as well as enhancing the adaptability and resilience of tomato plants.

In our study, we observed that under all three light conditions, the activation of ripening‐associated TFs and metabolic/ripening genes was closely linked to metabolic and ripening changes (Figures 3, S6A–C). As previously reported, the supplementation of blue light enhances flavonoid accumulation in tomato fruits, thereby promoting changes in fruit color (He et al., 2022; Liu et al., 2022). Blue light supplementation has been shown to increase flavonoid levels in tomato fruits, leading to altered fruit colors (Figures 1B, S1B), which is consistent with the BLS‐induced orange color of the fruits accompanied by higher flavonoids in our study. Self‐organizing map and co‐expression analyses revealed that the expression of *SlDML2*, a key DNA demethylase gene, is highly correlated with major TFs and metabolic/ripening genes across different light conditions (Figure 3). We further confirmed that the transcriptional induction of critical genes is driven by DNA demethylation of their promoters during fruit development and ripening (Figures 4C, D, S7, 8). It is consistently observed that promoter regions are among the most substantially affected areas, which suggests targeted epigenetic regulation rather than a random, genome‐wide shift (Figure S20). Differentially methylated region (DMR) enrichment analysis conducted between different samples allows for a more accurate assessment of the biological significance of these alterations in methylation levels (Figure S20). Previous studies in plants have demonstrated that even relatively modest methylation changes at promoter regions can exert a substantial influence on gene expression (Lang et al., 2017; Huang et al., 2019). This demethylation process was substantially accelerated under RLS and BLS (Figures 4B, S7), with SlPHYB2 in RLS and SlCRY1a in BLS influencing this effect (Figures 2, S4E–I). We double checked the expression of key photosynthesis genes under different light conditions and found no difference (Figure S21). This indicates the different color of ripening fruit under RLS and BLS was unlikely due to the change of photosynthesis. Instead, the differences of flavonoids and carotenoids contents are the determining factor for the color of mature fruit at ripe stage (Figures 1, S1). In addition, although we saw changes in the size of fruit under different conditions (Figure S1A), the expression of key fruit‐size genes was quite similar among different conditions (Figure S22). Taking all these together, light signals play a regulatory role in regulating *SlDML2* and its downstream targets involved in ripening and metabolism. Notably, as a multifunctional gene, *SlDML2* is considered crucial in balancing fruit ripening and disease resistance (Lang et al., 2017; Zhou et al., 2023).

Co‐expression analysis and Y1H screening suggest that SlHY5 may interact with *SlDML2*, thereby connecting light signals with epigenetic regulation (Figures S15A, B, 5D). SlHY5 binds to the G‐Box cis‐element in the *proSlDML2* promoter and activates its transcription (Figure 6). In the *SlHY5‐*RNAi line, the induction of *SlDML2* by RLS or BLS is absent (Figure 5D). Conversely, the DNA methylation level of the *proSlHY5* promoter decreased under RLS or BLS in the *sldml2‐*Cas9 mutant fruits (Figure S17). Therefore, the expression of *SlHY5* in the mutants was no longer induced by RLS and BLS (Figure S15C). Our re‐analysis of DNA methylation levels at the *HY5* locus in WT and *sldml2* plants under normal light conditions (Figure S15D), using data from Lang et al. (2017), reveals hypermethylation at the *proSlHY5* promoter region in the *sldml2* mutant. This hypermethylation correlates with reduced *HY5* expression (Niu et al., 2025), demonstrating that the expression of *HY5* is regulated by SlDML2. These results suggest that although SlHY5 acts upstream of SlDML2, its expression is also regulated by SlDML2, indicating mutual regulatory interactions between light signaling and DNA methylation in tomato.

Previous studies have reported that HY5 can cooperate with other components of the light signaling pathway to regulate carotenoid accumulation in tomato and other horticultural crops (Wang et al., 2018, 2021; Lu and Zhu, 2022). HY5 acts as a promotive factor, while PIFs, which respond to both light and temperature, function as inhibitors, making them crucial integrators of various environmental factors (Lee et al., 2007; Sreeramaiah and Vinod, 2017; Wang et al., 2018). Studies have found that genes involved in carotenoid and anthocyanidin biosynthesis, as well as ethylene signal transduction, are direct targets of SlHY5. Additionally, SlHY5 influences the translation efficiency of numerous ripening‐related genes (Wang et al., 2018; Fang et al., 2019). It regulates fruit ripening not only by transcriptionally regulating specific molecular pathways but also by exerting post‐transcriptional control through effects on protein translation mechanisms (Wang et al., 2021). Recent research has underscored the pivotal role of HY5 in fruit ripening processes. Specifically, HY5 directly modulates the transcriptional regulation of genes involved in carotenoid biosynthesis and sugar metabolism. Comparative analyses revealed that red and blue light spectra exerted a pronounced effect on HY5 protein accumulation (Wang et al., 2025). In this study, we further confirmed the broad regulatory role of SlHY5 in tomato ripening and metabolism by directly regulating the expression of *SlDML2* to influence genome‐wide DNA methylation levels. Unlike CG and CHG methylation, CHH methylation was substantially increased during ripening, indicating that CHH methylation may play distinct roles from CG and CHG types in tomato ripening (Figures 4, S10–12). Additionally, some studies (Gent et al., 2013; Lang et al., 2017; Niu et al., 2025) have shown that CHH methylation at the promoter region may be associated with gene activation. Notably, ripening was substantially delayed in the *slhy5* mutant compared to the WT. Accordingly, the accumulation of CHH methylation in *slhy5* was inhibited (Figure S17).

Our study suggests that the SlHY5–SlDML2 module may play a central role in mediating light‐quality‐regulated ripening and metabolic changes in tomato fruit. Consistent with this, silencing or knockout of *SlHY5* has been shown to reduce anthocyanin and carotenoid content (Wang et al., 2021). Beyond its role in fruit metabolism, HY5 is crucial for light‐regulated development and stress responses in plants. The hypocotyls of *slhy5* mutants exhibit hypersensitivity to multiple light treatments, including red, far‐red, blue, and UV light (Zhang et al., 2022a). Furthermore, HY5 binds to the promoters of core circadian clock elements, such as *TOC1* (*Timing of Cab Expression 1*), *ELF4* (*Early Flowering 4*), *CCA1* (*Circadian Clock‐Associated 1*), and *LHY* (*Late Elongated Hypocotyl*), thus regulating their expression and driving the circadian clock (Lee et al., 2007; Delker et al., 2014). HY5 also protects against damage from intense light exposure by binding to the promoter of *ELIP2* (*Early Light‐Induced Protein 2*) and inducing its transcription, thereby enhancing photoprotection in plants. Additionally, HY5 is influenced by other environmental factors, such as temperature (Delker et al., 2014; Sreeramaiah and Vinod, 2017). Studies have shown that HY5 functions as a negative regulator in temperature‐mediated morphogenesis, with elevated temperatures suppressing its transcription and protein activity (Lee et al., 2021; Bian et al., 2022). *HY5* levels are regulated by low temperatures both transcriptionally and post‐translationally through a C‐repeat binding factor (CBF)‐ and abscisic acid (ABA)‐independent pathway, where HY5 supports cold‐induced gene expression by binding to the Z‐box and other cis‐acting elements, ensuring effective cold acclimation in Arabidopsis (Sreeramaiah and Vinod, 2017). Research in apples suggests that MdBBX20 synergistically promotes anthocyanin synthesis in apple skin by interacting with MdHY5 (Fang et al., 2019). A transcriptional cascade involving HY5‐MYB15‐CBFs plays an essential role in the low‐temperature response, with HY5 and MYB15 working together to enhance CBF‐dependent cold tolerance in tomatoes (Zhang et al., 2020). The tomato *SlHY5* gene has also been shown to integrate temperature, light, and hormone signals, balancing plant growth and enhancing low‐temperature resilience (Chi et al., 2025; Fu et al., 2024; Jia et al., 2024; Lin et al., 2025b; Sreeramaiah and Vinod, 2017; Wang et al., 2018). As light and temperature are both crucial factors in regulating crop growth and metabolism, the SlHY5–SlDML2 module may serve as a core component for environment‐driven metabolic changes in tomato. A previous study identified SlJMJ7 as a direct repressor of *SlDML2*, which in turn suppresses the transcription of ripening‐associated genes through DNA methylation (Ding et al., 2021). SlDML2‐mediated demethylation is implicated in the regulation of expression profiles of specific transcription factors and structural genes associated with fruit ripening (Zhang et al., 2022b). Concurrently, key TFs and structural genes themselves are subject to demethylation, which in turn modulates SlDML2 expression, forming a reciprocal regulatory loop (Zhang et al., 2022b; Bai et al., 2025; Niu et al., 2025). Notably, the application of red or blue light serves to potentiate this regulatory interplay. It may be a common mechanism by which environmental signals regulate plant metabolomes through changes in DNA methylation. In particular, the regulation of DNA methylation by light and temperature has been widely observed across species. For example, in Arabidopsis, the UVB photoreceptor UVR8 interacts with DRM2 (Domains Rearranged Methyltransferase 2), a *de novo* DNA methyltransferase, thereby inhibiting DRM2‐mediated DNA methylation and enabling transcriptional de‐repression (Jiang et al., 2021). Similarly, UVB exposure induces DNA methylation changes in maize and grapevine (Rius et al., 2016; Marfil et al., 2019; Jiang et al., 2021). In tomatoes, chilling stress alters genome methylation, leading to flavor loss in the fruit, while heat stress in soybeans results in hypomethylation in root and hair cells (Hossain et al., 2017). Beyond light and temperature, numerous other internal and external cues can also induce shifts in plant DNA methylation. For example, Pi starvation leads to widespread DNA methylation changes in rice and tomato (Tian et al., 2021), and biotic stresses, such as bacterial pathogens, trigger genome‐wide hypermethylation in Arabidopsis (Dowen et al., 2012). This suggests that plants may respond to environmental changes by adjusting genome‐wide methylation levels to regulate metabolism, a potentially universal mechanism worthy of further study across diverse plants and conditions.

In summary, this study systematically unveils the role of SlDML2‐mediated DNA demethylation in light‐mediated metabolic regulation in plants. Additionally, this research approach lays a foundation for investigating how other environmental factors influence crop growth and quality. These findings hold valuable implications for future breeding and production practices.

## MATERIALS AND METHODS

### Plant materials and growth conditions

Wild‐type tomato (*Solanum lycopersicum* cv. MicroTom) seeds were sourced from PanAmerican^TM^ Seed Company. Seedlings were grown in a climate‐controlled chamber set at 24°C ± 1°C, with a daily light/dark cycle of 16/8 h, relative humidity of 65%, and a maximum light intensity (PPFD) of ∼250 ± 20 μmol/m^2^/s from LED lights at the top of the seedlings, following established protocols (Xie et al., 2019; Paponov et al., 2020; He et al., 2022; Yuan et al., 2022). Plants were considered to have reached the flowering stage at 49 DPG when over 50% of seedlings had flowered. Flowers at anthesis (0 DPA) were tagged and recorded.

### Light treatment and sample collection

At the flowering stage, plants were divided into three groups subjected to different light conditions (three spectra). The PPFD across all conditions was maintained at 250 ± 20 μmol/m^2^/s at the top of the plants. One group was kept under the original control light, peaking at 588 nm, and served as the control. The other two groups were exposed to an additional 30% red light (peak at 633 nm, white:yellow = 7:3) and 30% blue light (peak at 456 nm, white:blue = 7:3), respectively. These treatments were designated as 30% RLS and 30% BLS.

Tomato pericarp samples were collected at the following fruit stages: 10, 20, 30, 35, 40, 44, 47, 50, and 55 DPA. To control for circadian gene effects, all samples were harvested at 2 p.m. (8 h after lights were turned on) each day. Pericarp samples from eight to 10 fruits from at least three plants were pooled as one biological replicate, with three biological replicates were collected for each time point. Samples were immediately flash‐frozen in liquid nitrogen and stored at −80°C.

### Measurement of carotenoids

Carotenoids were extracted and measured following a previously described method with minor modifications (Shraim et al., 2021). Briefly, 100 mg of dried pericarp powder was extracted using 1 mL of extraction buffer (n‐hexane:acetone:methanol = 2:1:1, by volume). Carotenoid metabolites were analyzed on a SCIEX Triple Quad^TM^ 5500 LC‐MS/MS platform, managed with Analyst 1.6.3 software (AB SCIEX), using Thermo Accucore C30 100 × 2.1 (mm) 2.6 μm as the LC analytical. Mass spectrometry was performed with an atmospheric pressure chemical ionization (APCI) source in positive ion mode. The mobile phase consisted of methanol (solvent A) and isopropanol (solvent B). The column was maintained at 35°C, with a 2‐μL injection volume. A pool of all extracts was used as quality control samples. For positive mode, the solvent flow was 0.6 mL/min, the gradient elution conditions (solvent B) were from 0% to 10% of solvent B in 1.1 min, and from 10% to 24% in 4 min, and from 24% to 30% in 6 min, and from 30% to 50% in 7 min, then conditions were held for 2 min, and the contribution of solvent B was decreased to 0% and maintained for another 2 min. The curtain gas was 40 psi, and the temperature was 500°C, the collision energy was 30 eV. Individual carotenoids were identified by co‐migration with carotenoid standards and quantified using a standard curve. Standards for *α*‐carotene (40395), *β*‐carotene (C4582), and lycopene (SMB00706) were purchased from Sigma (https://www.sigmaaldrich.cn/CN/zh, Darmstadt, Germany). Three biological replicates (see sample collection section) were analyzed for each sample.

### Measurement of total flavonoids

The extraction and measurement of total flavonoids were modified from a previous study (Shraim et al., 2021). In brief, 1 mL of 80% methanol was added to a 2‐mL Eppendorf tube containing 20 mg of freeze‐dried tomato powder. It was vortexed, then the metabolites were extracted with ultrasound for 10 min, and centrifuged at 4°C for 10 min. The supernatant was diluted 10 times. Then, 1 mg of rutin standard sample (B20771; Source Leaf Biology, Shanghai, China) was dissolved with 1 mL of 80% methanol. It was diluted with 80% methanol solution to a total of eight concentration gradients of 2,000 , 1,500, 1,000, 750, and 500, 250, 125, and 0 μg/mL to prepare the standard curve. Then, 20 μL of different dilution gradients of rutin standard or tomato pericarp extracts were injected into a 96‐well enzyme standard plate, and 30 μL of 5% sodium nitrite solution was added and left to react at room temperature for 6 min. After the reaction was completed, 10% aluminum chloride solution was added and shaken well, reacting again at room temperature for 5 min. Finally, 100 μL sodium hydroxide solution was added and mixed well, reacting at room temperature for 15 min. Absorbance was measured at 510 nm using an enzyme‐linked immunosorbent assay (Synergy H1; BioTek, Vermont, USA), and the total flavonoid content was calculated based on the regression equation from the standard curve. Three biological replicates (see sample collection section) were analyzed for each sample.

### Gas chromatography‐MS analysis

Total metabolites were extracted using a modified version of a previously reported method (Min et al., 2021). In short, 0.03 g of freeze‐dried plant material was placed in a 2‐mL spiral centrifuge tube, to which 300 μL of ultrapure water was added. The sample was vortexed, sonicated for 10 min. Then, 900 μL of methanol chloroform mixture (methanol:chloroform = 3:1 by volume) was added, and sonicated for 10 min (water:methanol chloroform mixture = 1:3 by volume), at 4°C, 12,000 rpm, for 10 min. Then, 400 μL of the supernatant was added to a 1.5‐mL high recovery injection bottle. Then 10 μL of dichlorophenylalanine aqueous solution (1 mg/mL) was added and vacuum‐dried at room temperature. Then, 400 μL of the supernatant was again added to a 1.5‐mL high recovery injection bottle for vacuum drying. The sample was dried with nitrogen gas, and 80 μL of methoxypyridine solution (currently used, 15 mg/mL, damas beta, Shanghai, China, cat. 683688) was added, and the cap was sealed. This was shaken for 30 s at 30°C, and mixed 220 rpm overnight for reaction, followed by 1 h of shaking at 70°C in 80 μL of bis‐methyl‐trimethylsilyl trifluoroacetamide (BSFTA) solution (containing 1% TMCS, cat CFCQ‐270121; Shanghai Ampu Experimental Technology Co. Ltd, Shanghai, China), then the cap was sealed. GC‐MS analysis was conducted on a time‐of‐flight (TOF)/MS system coupled with an Agilent 7890 Chromatograph (Agilent, CA, USA). Helium served as the carrier gas at a flow rate of 1 mL/min, with a spitless injection. Eluted compounds were ionized by electron ionization (EI) with an electron energy of 70 eV and an ion source temperature of 220°C. Full MS scans were conducted in the mass range of 33–600 *m*/*z*. Three biological replicates (see sample collection section) were analyzed for each sample.

### Liquid chromatography‐MS analysis

For metabolite extraction, 1 mL of methanol/water (80/20 v/v; HPLC‐grade; Honeywell, Seelze, Germany) was used as the extraction solvent on 20 mg of freeze‐dried powder. After ultrasound sonication for 10 min, the solution was centrifuged, and the supernatant was collected for LC‐MS analysis in both positive and negative ion modes. Each sample was analyzed in triplicate (see sample collection section). Liquid chromatography‐MS analysis was conducted on an AB SCIEX qTOF X500R system using a Hypersil Gold 1.9 μm C18 column (100 × 2.1 mm; Thermo Fisher Scientific, Massachusetts, USA) (Fu et al., 2021). The mobile phase consisted of 0.1% formic acid in water (Solvent A) and acetonitrile with 0.1% formic acid (Solvent B). The column was maintained at 40°C, with a 3‐μL injection volume. A pool of all extracts was used as quality control samples. For positive mode, the solvent flow was 0.28 mL/min, the gradient elution conditions (Solvent B) were from 2% to 25% of Solvent B in 9 min, and from 25% to 99% in 15 min, then conditions were held for 7 min, and the contribution of Solvent B was decreased to 2% and maintained for another 2.9 min. The curtain gas was 35 psi, and the temperature was 500°C, the collision energy was 35 eV and the collision spread was 15 eV. For negative mode, the solvent flow was 0.4 mL/min, the gradient elution conditions (Solvent B) were from 0% to 5% of solvent B in 3.1 min, and from 5% to 20% in 6 min, then increased to 50% in 11 min and to 100% in 15 min; after holding for 2 min, the contribution of Solvent B was decreased to 0%. The curtain gas was 35 psi, and the temperature was 500°C, the collision energy was −40 eV and the collision spread was 20 eV. Metabolite quantification was performed using SCIEX OS software (version 1.7). Data were median‐normalized, log‐transformed, mean‐centered, and divided by the square root of the standard deviation for each variable.

### RNA extraction and quantitative reverse transcription‐polymerase chain reaction

Total RNA was extracted from tomato pericarp using the Total RNA Extraction Kit (BIOFIT, Chengdu, China) following the manufacturer's instructions. Complementary DNA (cDNA) was synthesized from messenger RNA (mRNA) using a PrimeScriptTMRT reagent kit with a gDNA eraser (TaKaRa, Kyoto, Japan). qRT‐PCR was performed using iTaq Universal SYBR Green Supermix (Bio‐Rad, CA, USA). *SlUBI* served as the internal control, and relative gene expression levels were calculated using the Δ*C*_t_ method. Each sample was analyzed with three biological and three technical replicates. Primers used for qRT‐PCR are listed in Table S8.

### RNA sequencing

RNA sequencing was conducted by Novogene (Novogene Co. Ltd, Beijing, China) using a TargetAmp two‐round aRNA amplification kit (You et al., 2024). Sequencing for all tomato samples was performed on an Illumina HiSeq. 2500 system (Illumina, San Diego, CA, USA), as previously described (You et al., 2024). Raw reads were trimmed and filtered to remove adapters and low‐quality bases using SOAPnuke (Chen et al., 2018). This resulted in a total of 1,591,848,951 raw reads and 1,538,149,208 clean reads. These clean reads were then used for assembly and gene abundance calculations. The high‐quality reads were aligned to the tomato iTAG4.0 genome (https://solgenomics.net/organism/Solanum_lycopersicum/genome) using HISAT 2 with default parameters. The mapped reads for each sample were assembled through a reference‐based approach using StringTie, with a default alignment of 95%. StringTie was also used to generate TPM values based on gene length and read counts mapped to each gene. Genes with an average TPM ≥ 1 were considered expressed. Hierarchical clustering of all genes was performed using one minus Pearson correlation as the distance metric.

### Co‐expression/co‐regulation cluster identification and analysis

Co‐expression and co‐regulation analyses were performed on samples collected at nine different time points under three light conditions using the *k*‐means and ggplot2 functions in RStudio (He, 2025). *K*‐means clustering was applied to metabolites under white light conditions. Based on the clustering results, genes in the transcriptome data with Pearson correlation coefficients > 0.8 to any metabolite were identified (Li et al., 2020c; Xiang et al., 2025). The expression trends of the corresponding metabolites and genes under red and blue light conditions were then plotted using ggplot2 in RStudio. Euclidean distance‐based hierarchical cluster analysis (HCA) was applied to explore the variance distribution and classification patterns among samples. Discriminant analysis of the tomato samples was carried out with principal component analysis (PCA) and partial least square‐differential analysis (PLS‐DA).

### Self‐organizing map method

For the SOM analysis, transcriptomic data was filtered to remove contigs with TPM values of zero for more than half of the treatments or with zero expression variance across samples. The filtered data was then normalized to a mean expression level of zero with unit variance across conditions. Self‐organizing map analysis and visualization were conducted in R (version 4.2.3; R Core Team 2023) using the Kohonen package (version 2.0.19) (Wehrens and Buydens, 2007; Lin et al., 2025a). The map size was selected to assign ∼200 contigs per node, with a map shape that maintained the same ratio between edge lengths as between the two largest eigenvalues of the data. A toroidal map was used to ensure uniform neighborhood connectivity across all nodes, minimizing boundary effects in calculating neighborhood distances. Two quality metrics, within‐node distance and internodal distance, were evaluated to assess the SOM's fit to the data. Nodes were considered high quality if both metrics fell within the lowest quartile (25%) of all nodes.

### Whole genome methylation sequencing and analysis

Genomic DNA from tomato fruit was extracted using a DNeasy Plant Maxi Kit (Qiagen, Hilden, Germaany) per the manufacturer's protocol and then prepared for library construction. Lambda DNA served as an unmethylated control for calculating bisulfite conversion efficiency. Libraries were sequenced on the Illumina Hi‐Seq. 2500 platform (BGI, Shenzhen, China) (Li et al., 2020a), with two replicates per sample. After sequencing, low‐quality reads were filtered out using SOAPnuke v1.5.6 (identifying reads as low quality if more than 40% of their bases had a quality score below 10). Cleaned reads were then mapped to the tomato reference genome (ITAG4.0) using BSMAP (v2.90) allowing up to two mismatches and one best alignment (Xi and Li, 2009). Differentially methylated regions were identified by methdiff function in BSMAP, with a significance threshold of *P* < 0.01 and minimum methylation differences of 0.2 for CG, 0.1 for CHG, and 0.05 for CHH contexts. Genomic distributions of DMRs were annotated by ChIPseeker (Yu et al., 2015) control data sets were obtained by randomly selected, equal length regions in ITAG4.0 genome with the bedtools shuffle function (v2.27.1) (Quinlan and Hall, 2010).

### Vector construction and generation of transgenic lines

To generate RNAi plants, the coding sequence (CDS) of target genes was amplified and cloned into pBWA(V)HS‐RNAi vectors using a homologous recombination system (C117‐01; Vazyme, Nanjing, China) (Zhang et al., 2023). For gene editing, the pHSbdcas9i vector was employed (Zhang et al., 2023), and guide RNAs were designed using CHOPCHOP (http://chopchop.cbu.uib.no/), Primers used are listed in Table S8. Plasmids with correct insertions were introduced into *Agrobacterium tumefaciens* strain EHA105, and tomato transformation was carried out following previously established protocols (Ying et al., 2020).

### Yeast one‐hybrid screening assay

The tomato cDNA library for this study was obtained from Yuanbao Biotech (Nanjing, China). To screen the yeast library, synthetic dropout (SD)/−His−Leu−Trp media containing varying concentrations (0, 20, 40, 60, 80, and 100 mmol/L) of 3‐AT (3‐amino‐1, 2, 4‐triazole) was used with Y187 yeast strain. A 0 mmol/L 3‐AT culture served as the control, and 80 mmol/L 3‐AT was selected for subsequent screening. Two replicates (each plate of yeast considered one replicate) with over 600 positive clones each were collected and pooled for isolation of pHIS2. Truncated DNA from pHIS2 containing the insertion sequence was amplified by PCR, and high‐throughput sequencing was conducted on an Illumina Hi‐Seq. 2500 instrument. Raw data were converted to fasta format using fq2fa, then aligned to the tomato iTAG4.0 genome with parameters set to −outfmt 6 and an e‐value of 1 e^−3^.

### DNA‐affinity purification sequencing and data analysis

DNA‐affinity purification sequencing was conducted following established protocols at Bluescape Hebei Biotech (Baoding, China) with two independent biological replicates (Sun et al., 2023; Wang et al., 2023; Zhao et al., 2024). Fresh MicroTom fruits were used for genomic DNA extraction and library preparation, using the MICH TLX DNA‐Seq Kit (Cat# NGS0602; Bluescape Hebei Biotech Co., Ltd, Baoding, China). The coding sequence of SlHY5 was cloned into a pFN19K HaloTag T7 SP6 Flexi expression vector, and the Halo‐SlHY5 fusion protein was expressed using the TNT SP6 Coupled Wheat Germ Extract System (Promega, Wisconsin, USA), following the manufacturer's instructions, in a 50‐μL reaction incubated at 37°C for 2 h. The expressed protein was captured on Magne Halo Tag Beads (Promega, Wisconsin, USA), and SlHY5‐bound beads were subsequently incubated with adapter‐ligated gDNA libraries. Eluted DNA fragments were sequenced on an Illumina NovaSeq platform, and negative control mock DAP‐Seq libraries were prepared without protein. Peaks were merged from the two biological replicates using MACS2 callpeak and Homer software, with a significance threshold of *Q* < 0.05. Motif discovery was performed using MEME‐ChIP software (Machanick and Bailey, 2011; Ying et al., 2020). Bound peaks were visualized in Integrative Genomics Viewer (IGV) and annotated using ChIPseeker software (Yu et al., 2015; Wang et al., 2022a).

### Chromatin immunoprecipitation qPCR assay

The open reading frame (ORF) sequence of *SlHY5* was amplified using homologous recombination and cloned into the pCAMBIA‐1306 vector, containing a 3×FLAG tag, to generate the 35S::FLAG‐SlHY5 construct. Chromatin immunoprecipitation qPCR assays were performed on the transgenic line 35S::FLAG‐*SlHY5* following a modified version of an established protocol (Zhang et al., 2023). Samples were treated with a 1% formaldehyde crosslinking buffer and subjected to three rounds of vacuum infiltration. Crosslinking was terminated by adding glycine, and chromatin was fragmented using ultrasound treatment, followed by centrifugation for purification. A portion of the fragmented chromatin was reserved as the input group. Chromatin was isolated using Honda buffer (0.44 mol/L sucrose, 1.25% Ficoll, 2.5% dextran T40, 20 mmol/L HEPES pH 7.4, 10 mmol/L MgCl_2_, 0.5% Triton X‐100, 1 mmol/L dithiothreitol, and a protease inhibitor cocktail). Nuclei were lysed in a buffer containing Tris‐HCl (pH 8), ethylenediaminetetraacetic acid, sodium dodecyl sulfate, phenylmethylsulfonyl fluoride, and protease inhibitors. Chromatin immunoprecipitation was conducted using a monoclonal anti‐FLAG antibody (Cat No. F1804; Sigma‐Aldrich, Darmstadt, Germany). Immunoprecipitation and DNA isolation/purification were performed using the EpiTect ChIP OneDay Kit (Qiagen, Hilden, Germany) per the manufacturer's instructions. Primers used in the qPCR assay are listed in Table S8, with each set repeated at least three times.

### Transient dual‐luciferase reporter assay

Full‐length coding sequences of *SlHY5* were cloned into the pEAQ vector to create effectors, and the promoter of *SlDML2* was cloned into the pGREEN II 0800‐LUC double vector. Fluorescence intensities of LUC and Renilla (REN) were measured using the Dual‐Luciferase Reporter Assay System, following the manufacturer's instructions (Promega, Madison, Wisconsin, USA). Each sample was analyzed with three biological replicates.

### Yeast one‐hybrid assay

The Y1H assay was conducted as previously described (Yan et al., 2016). Briefly, the pB42AD‐*SlHY5* plasmid was co‐transformed into the EGY48a yeast strain along with pLacZi‐*proSlDML2* using the LiAc‐mediated transformation method. Transformants were grown on synthetic minimal double dropout medium lacking Trp and Ura (SD/−Trp/−Ura) at 30°C for 3 d. Yeast cells from the SD/−Trp/−Ura plates were suspended in sterile water, then spotted onto SD/−Trp/−Ura medium with X‐Gal at 30°C for 24–36 h. Each yeast colony was treated as a biological replicate, with six replicates analyzed per transformant.

### Electrophoretic mobility shift assay

Eco*R1* and Xho*I* restriction sites were added to both ends of the ORF of the *SlHY5* gene (primers are listed in the Table S8), and the gene was constructed into the pCold™‐DNA prokaryotic expression vector (#3360_3364; Takara, Kyoto, Japan) by homologous recombination. The vector was transformed into BL21 competent cells to induce protein expression, and the protein was purified using magnetic beads (#SM008005; ACE, Changzhou, China). Biotin‐labeled probe primers for both the mutant and non‐mutant sequences were synthesized (#GS008; Beyotime, Shanghai, China). The purified protein was mixed with probes containing the CArG‐box element and the mutant element at room temperature for incubation. The EMSA experiment was conducted using a binding kit (#20148X, Thermo Fisher Scientific) and a luminescent imaging kit (#89880, Thermo Fisher Scientific, Massachusetts, USA). The migration bands were finally observed under a charge‐coupled device camera (Tanon 5200). The probe primers are listed in the Table S8.

### Quantification and statistical analysis

Quantification and statistical parameters are detailed in the figure legends. Statistical analyses were conducted using a two‐sided Student's *t*‐test for two‐group comparisons or one‐way analysis of variance (ANOVA) followed by Duncan's test for multiple group comparisons. The *P*‐value < 0.05 was considered statistically significant. All graphs were produced using GraphPad Prism v9.3.3 for macOS or Microsoft Excel for macOS.

## CONFLICTS OF INTEREST

The authors declare no competing interests.

## AUTHOR CONTRIBUTIONS

Y.Z. and Z.Zhang. conceptualized the project; J.Z., Z.Zhang., Y.C., Y.W., and Q.H. conducted most of the experiments; J.Z., Z.Zhang., G.L., and X.Z. participated in the sample collection and light configuration; J.Z., Z.Zhang., Y.W., W.L., Y.L., and Z.Zhong conducted bioinformatic analysis; K.Z. generated the *slhy5* knockout mutants; Y.X. and E.T. performed SOM analyses; D.S. and M.L. provided technical support for ripening assays and seeds of tomato ripening mutants; Z.Zhang., Y.Z., J.Z., Z.Zhong, and M.L. wrote the manuscript with input from all authors. All authors reviewed and edited the manuscript.

## Supporting information

Additional Supporting Information may be found online in the supporting information tab for this article: http://onlinelibrary.wiley.com/doi/10.1111/jipb.70066/suppinfo

**Figure S1.** Red or blue light supplements accelerated the accumulation of carotenoids and total flavonoids in tomato fruit
**Figure S2.** Conjoint analysis of metabolome and transcriptome data in the TomLED
**Figure S3.** KEGG and GO enrichment of genes in Cluster I
**Figure S4.** Tissue‐specific expression pattern of key lighter receptor genes
**Figure S5.** Relative expression levels of *SlPHYB1*, *SlPHYB2* in *SlPHYB2*‐RNAi T0 plants and *SlCRY1a*, *SlCRY1b* in *SlCRY1a*‐RNAi T0 plants
**Figure S6.** Major ripening‐related TFs are highly co‐expressed with ripening‐associated genes
**Figure S7.** Whole genome DNA methylation is critical for light‐regulated fruit metabolic and ripening changes
**Figure S8.** mCHH methylation levels of *SlRIN* promoter in tomato at 40 DPA under three light conditions
**Figure S9.** Relative gene expression of key metabolic and ripening genes at 40 DPA
**Figure S10.** The promoter CG methylation level of key metabolic and ripening genes at 40 DPA
**Figure S11.** The promoter CHG methylation level of key metabolic and ripening genes at 40 DPA
**Figure S12.** The gene expression and promoter CHH methylation level of key metabolic and ripening genes at 40 DPA
**Figure S13.** The expression pattern of *SlDML2*
**Figure S14.** Gene editing of *SlDML2*
**Figure S15.** The interaction between SlHY5 and *SlDML2*
**Figure S16.** The transgenic lines of *SlHY5*
**Figure S17.** DNA methylation levels of *SlHY5* promoters during fruit ripening process
**Figure S18.** SlDML2‐mediated DNA demethylation was inhibited in *slhy5* plants
**Figure S19.** The DNA demethylation process of the promoter of key metabolic and ripening genes was significantly delayed in the *slhy5*‐Cas9 lines
**Figure S20.** Enrichment of DMRs across genomic regions
**Figure S21.** The expression level of genes related to photosynthesis under three light conditions in the TomLED
**Figure S22.** The expression level of genes related to weight and fruit diameter under three light conditions in the TomLED

**Table S1.** Summary of metabolome profiling for tomato fruits

**Table S2.** Quality of transcriptome data

**Table S3.** Summary of transcriptome profiling for tomato fruits

**Table S4.** Summary of co‐expression clusters

**Table S5.** Summary of SOM

**Table S6.** Summary of metabolites highly co‐expressed with *RIN* under three light conditions

**Table S7.** Summary of candidate genes identified in Y1H screen assay

**Table S8.** Summary of polymerase chain reaction with qRT‐PCR and PCR primers used in this study

**Table S9.** The peak matched to the motif in DAP‐Seq

**Table S10.** Ripening‐associated genes with methylation differences in SlHY5 DAP‐Seq data

## Data Availability

The data supporting the findings of this study are available within the paper and its supplementary information files. The raw data obtained from RNA‐seq and WGBS for tomato samples with different light treatments may be found in the National Genomics Data Center (https://ngdc.cncb.ac.cn/?lang=zh) with the accession numbers of PRJCA018419, PRJCA018495, respectively. The raw data of WGBS of WT and *slhy5* tomato fruit may be found at PRJCA030931.
