# Supplementary material for: Light regulates tomato fruit metabolome via SlDML2‐mediated global DNA demethylation
Source: J Integr Plant Biol. 2025 Oct 23;68(2):383–405. doi: 10.1111/jipb.70066 (PMC12863027; doi:10.1111/jipb.70066)
Supplement: Supplementary file 1 — Figure S1. Red or blue light supplements accelerated the accumulation of carotenoids and total flavonoids in tomato fruit Figure S2. Conjoint analysis of metabolome and transcriptome data in the TomLED Figure S3. KEGG and GO enrichment of genes in Cluster I Figure S4. Tissue‐specific expression pattern of key lighter receptor genes Figure S5. Relative expression levels of SlPHYB1, SlPHYB2 in SlPHYB2‐RNAi T0 plants and SlCRY1a, SlCRY1b in SlCRY1a‐RNAi T0 plants Figure S6. Major ripening‐related TFs are highly co‐expressed with ripening‐associated genes Figure S7. Whole genome DNA methylation is critical for light‐regulated fruit metabolic and ripening changes Figure S8. mCHH methylation levels of SlRIN promoter in tomato at 40 DPA under three light conditions Figure S9. Relative gene expression of key metabolic and ripening genes at 40 DPA Figure S10. The promoter CG methylation level of key metabolic and ripening genes at 40 DPA Figure S11. The promoter CHG methylation level of key metabolic and ripening genes at 40 DPA Figure S12. The gene expression and promoter CHH methylation level of key metabolic and ripening genes at 40 DPA Figure S13. The expression pattern of SlDML2 Figure S14. Gene editing of SlDML2 Figure S15. The interaction between SlHY5 and SlDML2 Figure S16. The transgenic lines of SlHY5 Figure S17. DNA methylation levels of SlHY5 promoters during fruit ripening process Figure S18. SlDML2‐mediated DNA demethylation was inhibited in slhy5 plants Figure S19. The DNA demethylation process of the promoter of key metabolic and ripening genes was significantly delayed in the slhy5‐Cas9 lines Figure S20. Enrichment of DMRs across genomic regions Figure S21. The expression level of genes related to photosynthesis under three light conditions in the TomLED Figure S22. The expression level of genes related to weight and fruit diameter under three light conditions in the TomLED [file JIPB-68-383-s009.doc]

**Light regulates tomato fruit metabolome via SlDML2-mediated global DNA demethylation**

**Supplemental Data**

Zhang *et al*

**
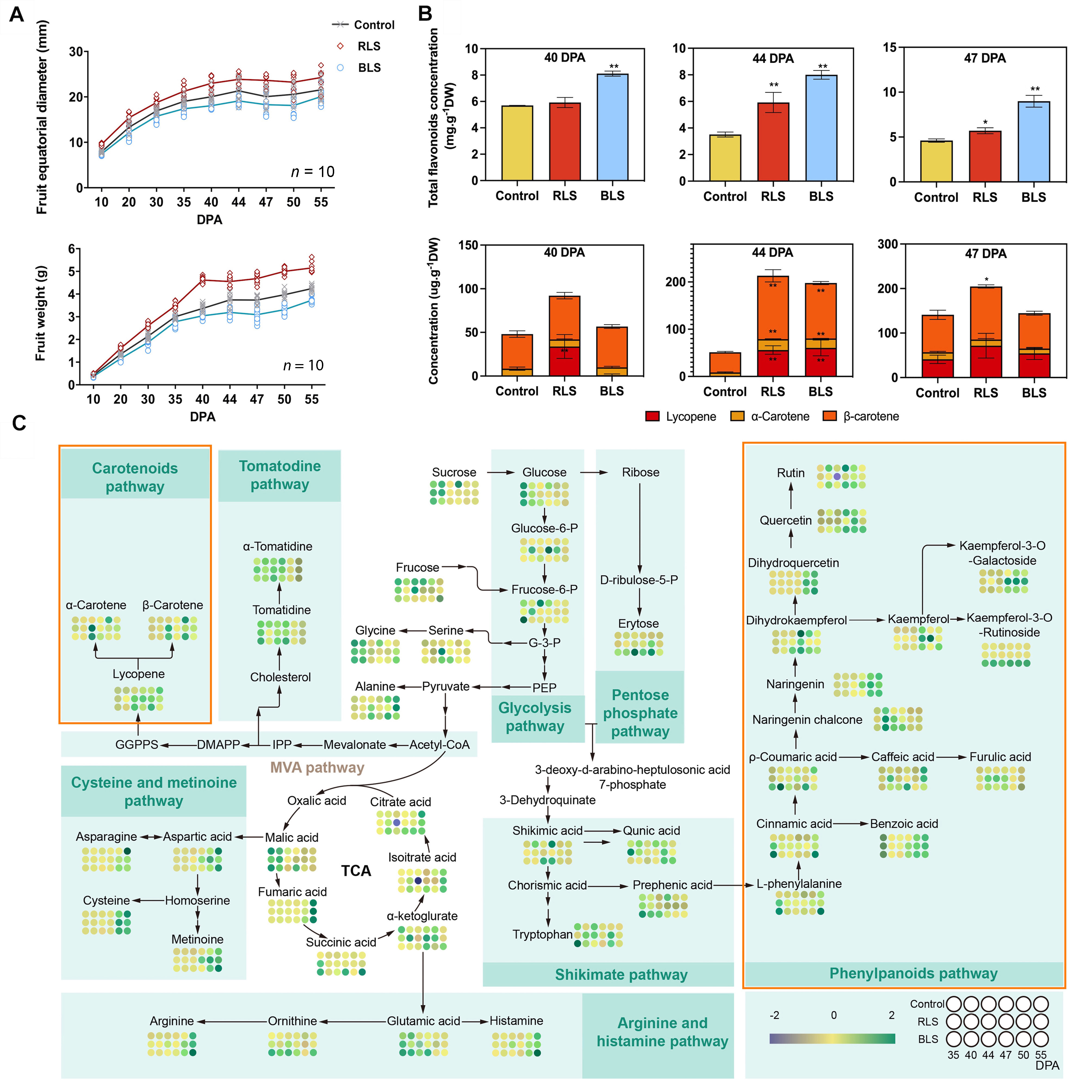
**

**Figure S1. Red or blue light supplements accelerated the accumulation of carotenoids and total flavonoids in tomato fruit**

**(A)** Fruit weight and diameter of tomato fruit under three light conditions. Error bars represent the standard deviation of ten biological replicates (*n* = 10). **(B)** The carotenoid and total flavonoid content contents of tomato fruit at 40, 44, and 47 DPA. ***P* < 0.01 and **P* < 0.05 indicate significant differences between control and RLS/BLS in the same metabolite of tomato fruit (Student’s *t*-test, *n* = 3). **(C)** A metabolic pathway scheme summarizing global metabolic changes occurring during the transition from green expanding to ripening processes (from 35 to 55 DPA) in tomato fruit under three light conditions. The biosynthesis of carotenoids and phenylpropanoids was significantly induced. Z-scores of data sets were standardized to 2 to −2. The color in green represents 2, while gray represents −2.





**Figure S2. Conjoint analysis of metabolome and transcriptome data in the TomLED**

**(A**, **C)** Principal component analysis of metabolome **(A)** and transcriptome **(B)** data of the nine sampling time points under three light conditions. The numbers next to the circles in the picture represent different stages of fruit development, while the circle color represents different light treatments. Yellow, red and blue colors were used to indicate control, RLS and BLS, respectively. **(B**, **D)** Series test of clusters of metabolites **(B)** and genes **(D)** in tomato fruit by *k*-means. A rigorous multiple test correlation (*r* ≥ 0.8) was used to select the genes that were significantly correlated with at least one metabolite, and the identified genes together with all metabolites were divided into ten clusters, respectively. Control light, RLS, or BLS was represented by yellow, red and blue colors, respectively. The axis represents various development stages of tomato fruits. The numbers shown in each box represent the metabolites and gene numbers across all the nine time points in each cluster. Data for Cluster I are shown in Figure 1C.


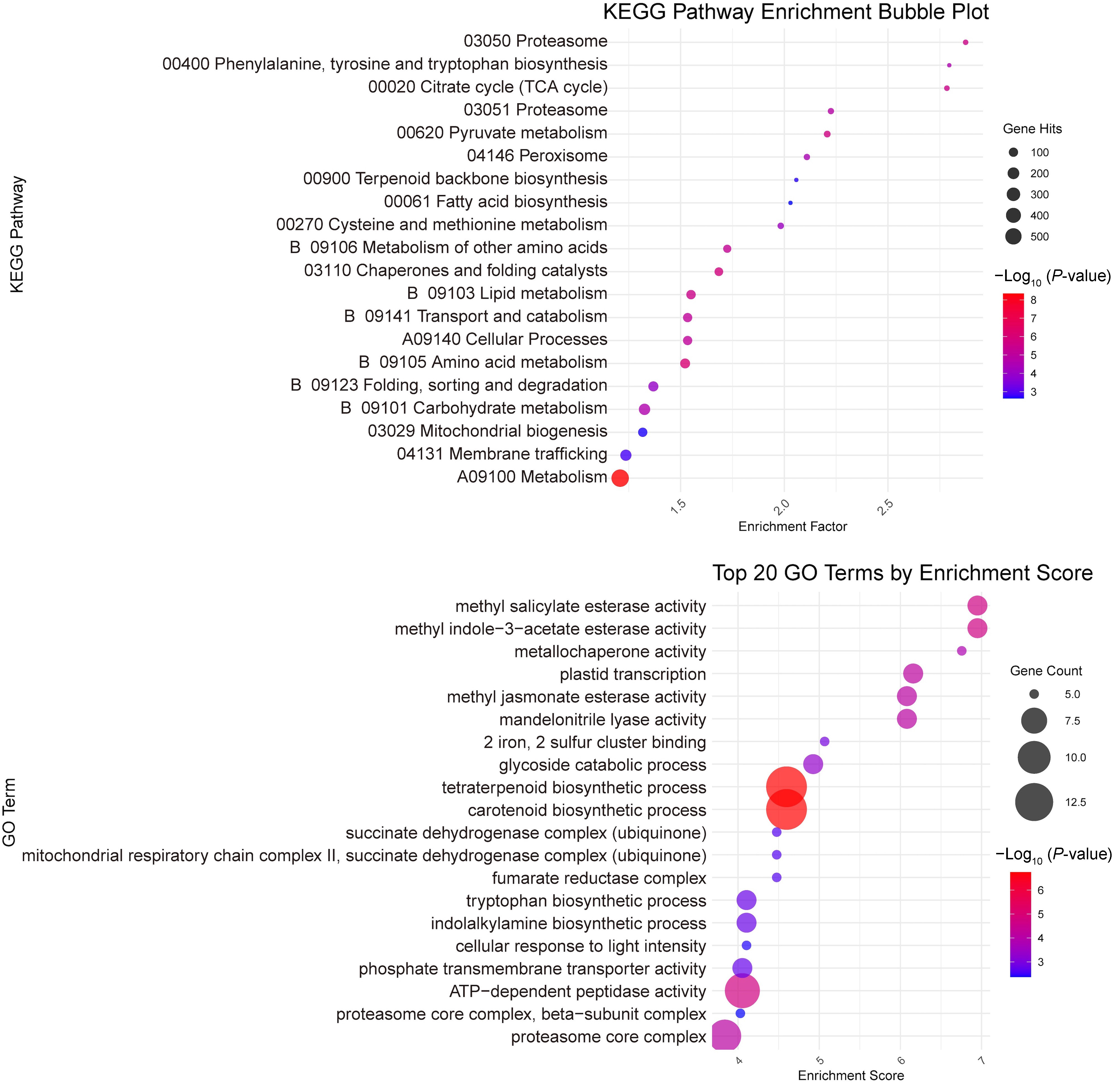


**Figure S3. KEGG and GO enrichment of genes in Cluster I**

X-axis represented enrichment ratio, and Y-axis represented top 20 KEGG or GO pathways. Gene hits: Bubble size represented number of genes annotated to a KEGG or GO Pathway. The color of the dots indicated the −Log_10_ (*P*-value).


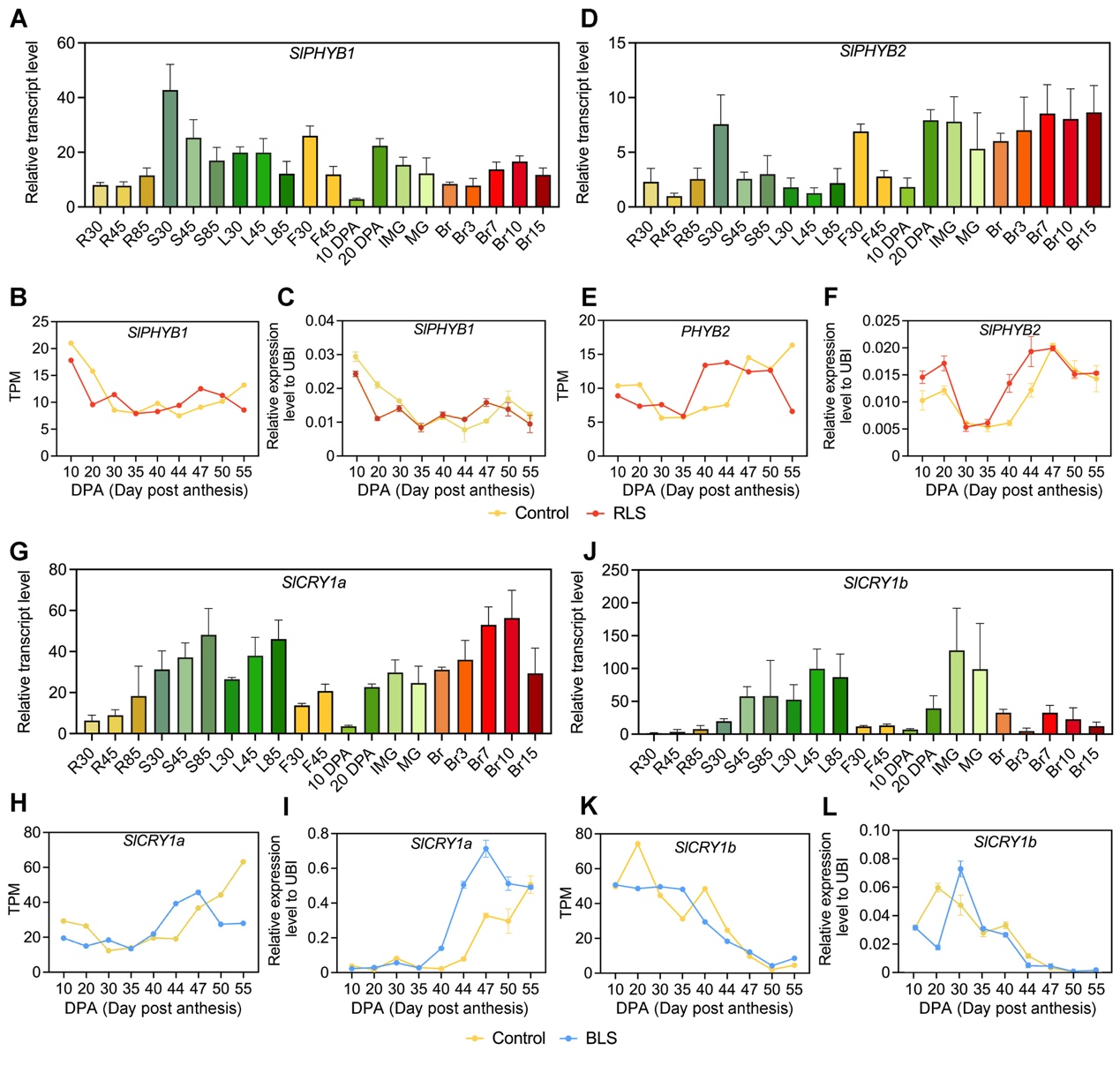


**Figure S4. Tissue-specific expression pattern of key lighter receptor genes**

**(A**–**C)** Tissue-specific expression patterns of *SlPHYB1* in tomato at different developmental stages from MMN database **(A)**, TomLED **(B)** and qRT-PCR **(C)**. R, S, L and F represent root, stem, leaf and flower, respectively. IMG MG and Br represent Immature green stage, mature green stage and breaker, respectively. Br3 (Breaker + 3), Br7 (Breaker + 7), Br10 (Breaker + 10), Br15 (Breaker + 15). **(D**–**F)** Tissue-specific expression patterns of *SlPHYB2* in tomato at different developmental stages from MMN database **(D)**, TomLED **(E)** and qRT-PCR **(F)**. **(G**–**I)** Tissue-specific expression patterns of *SlCRY1a* in tomato at different developmental stages from MMN database **(G)**, TomLED **(H)** and qRT-PCR **(I)**. **(J**–**L)** Tissue-specific expression patterns of *SlPHYB2* in tomato at different developmental stages from MMN database **(J)**, TomLED **(K)** and qRT-PCR **(L)**. Error bars represent the standard deviation of three biological replicates.

**
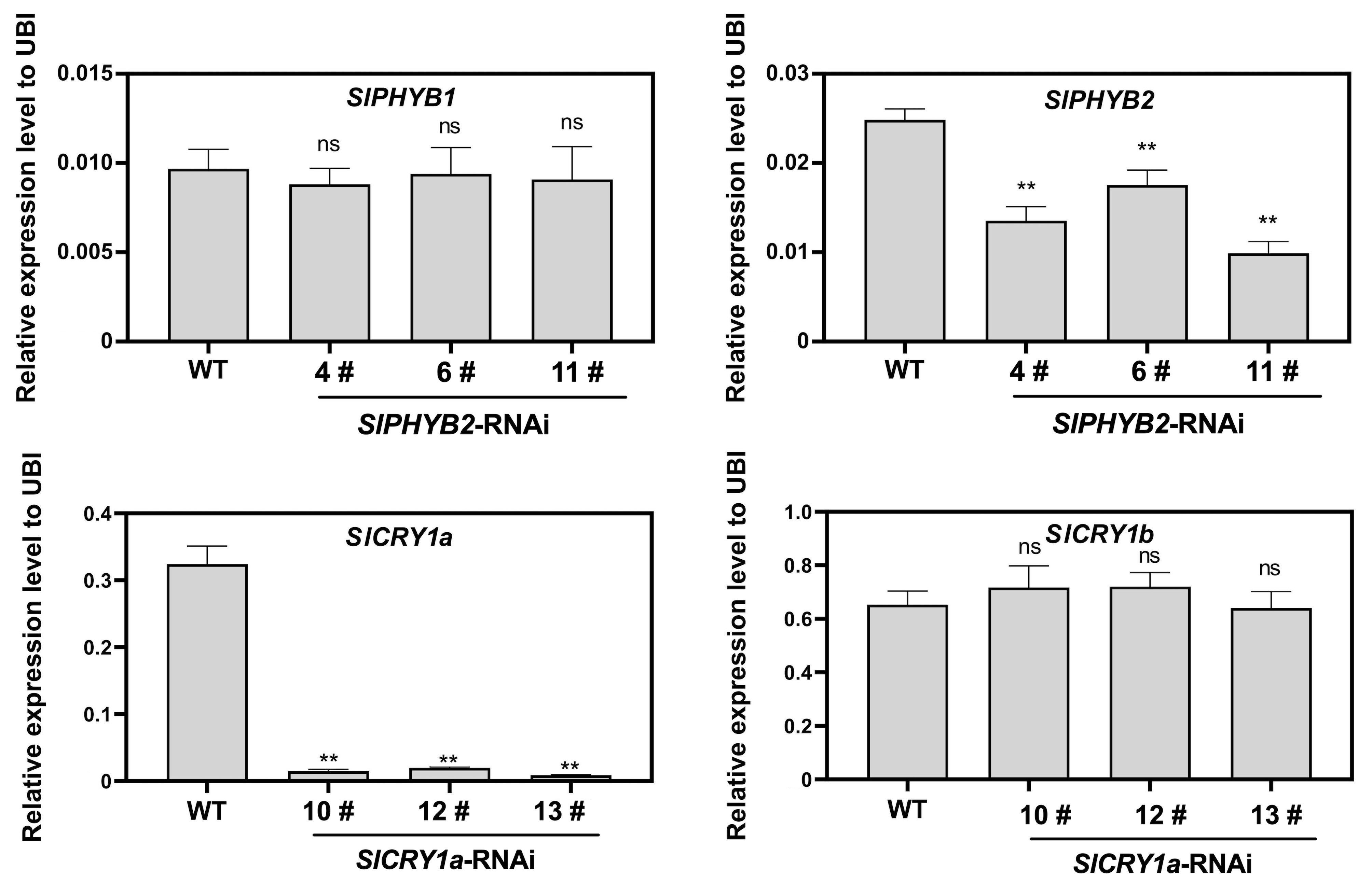
 Figure S5. Relative expression level of *SlPHYB1*, *SlPHYB2* in *SlPHYB2*-RNAi T0 plants and *SlCRY1a*, *SlCRY1b* in *SlCRY1a*-RNAi T0 plants**

*SlUBI* was used as internal control. ***P* < 0.01 indicates significant differences between control and RLS/BLS (Student’s *t*-test, *n* = 3).


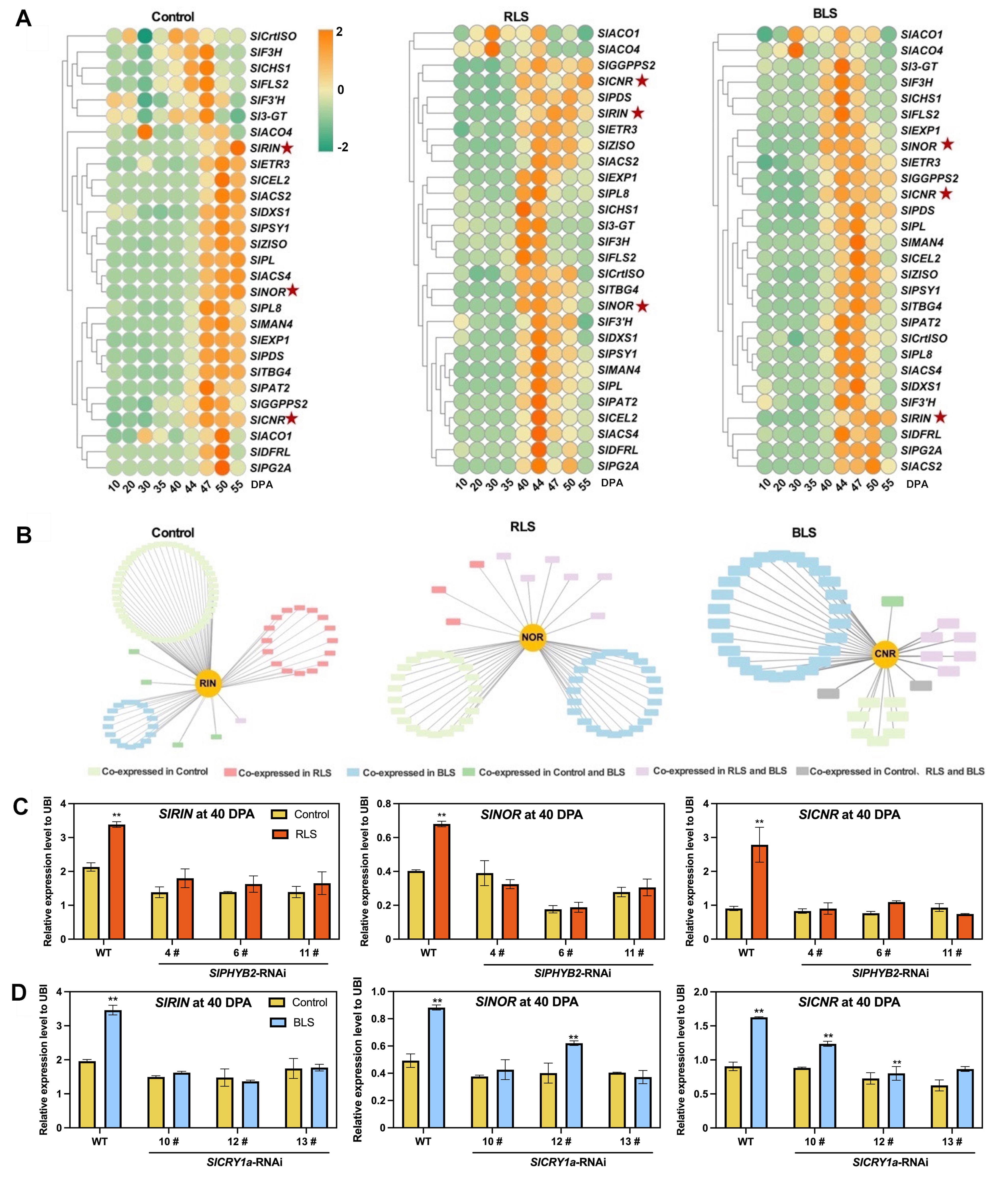


**Figure S6. Major ripening-related TFs are highly co-expressed with ripening-associated genes**

**(A)** Heatmap showing numbers of ripening-related genes co-expressed with *SlRIN*, *SlNOR* and *SlCNR* under three light conditions. Z-scores of data sets were standardized to −2 to 2. The color in orange represents 2, while green represents −2. **(B)** Co-expression network of metabolites highly associated with *SlRIN*, *SlNOR* and *SlCNR*. **(C)** Relative expression level of *SlRIN*, *SlNOR* and *SlCNR* in *SlPHYB2*-RNAi fruits at 40 DPA. (**D**) Relative expression level of *SlRIN*, *SlNOR* and *SlCNR* in *SlCRY1a*-RNAi fruits at 40 DPA. *SlUBI* was used as internal control. ***P* < 0.01 indicates significant differences between control and RLS/BLS in the same metabolite of tomato fruit (Student’s *t*-test, *n* = 3).


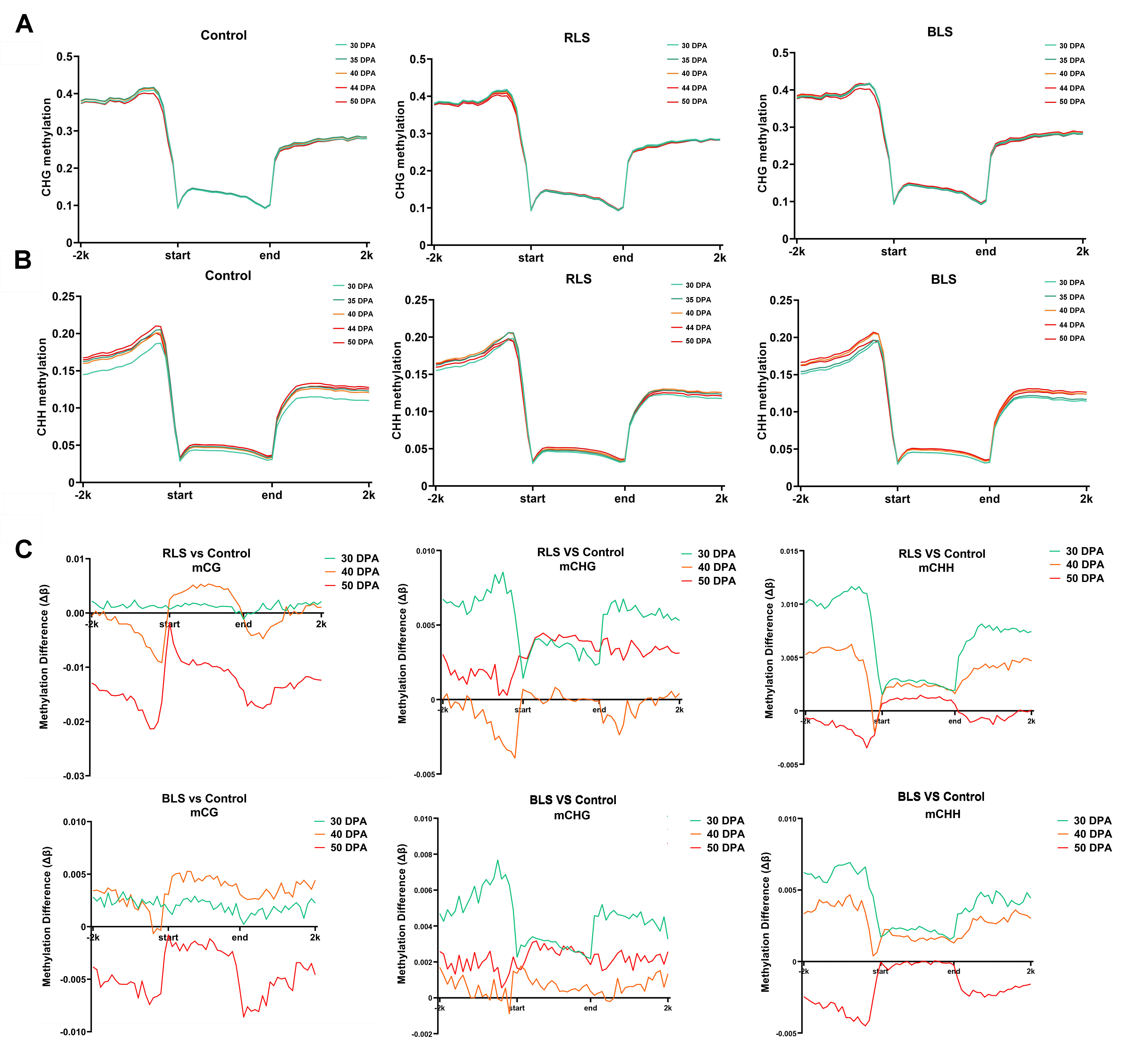


**Figure S7. Whole genome DNA methylation is critical for light-regulated fruit metabolic and ripening changes**

**(A)** Genome wide of mCHG methylation over gene body and flanking 2,000 bp of tomato fruits under three light conditions during fruit development and ripening. **(B)** Genome wide of mCHG methylation over gene body and flanking 2,000 bp of tomato fruits under three light conditions during fruit development and ripening. **(C)** mCG，mCHG and mCHH methylation difference of tomatoes under RLS/BLS with control light conditions. Differential DNA methylation: [Group A] vs [Group B] (Δβ = β {Group A} − β {Group B}).


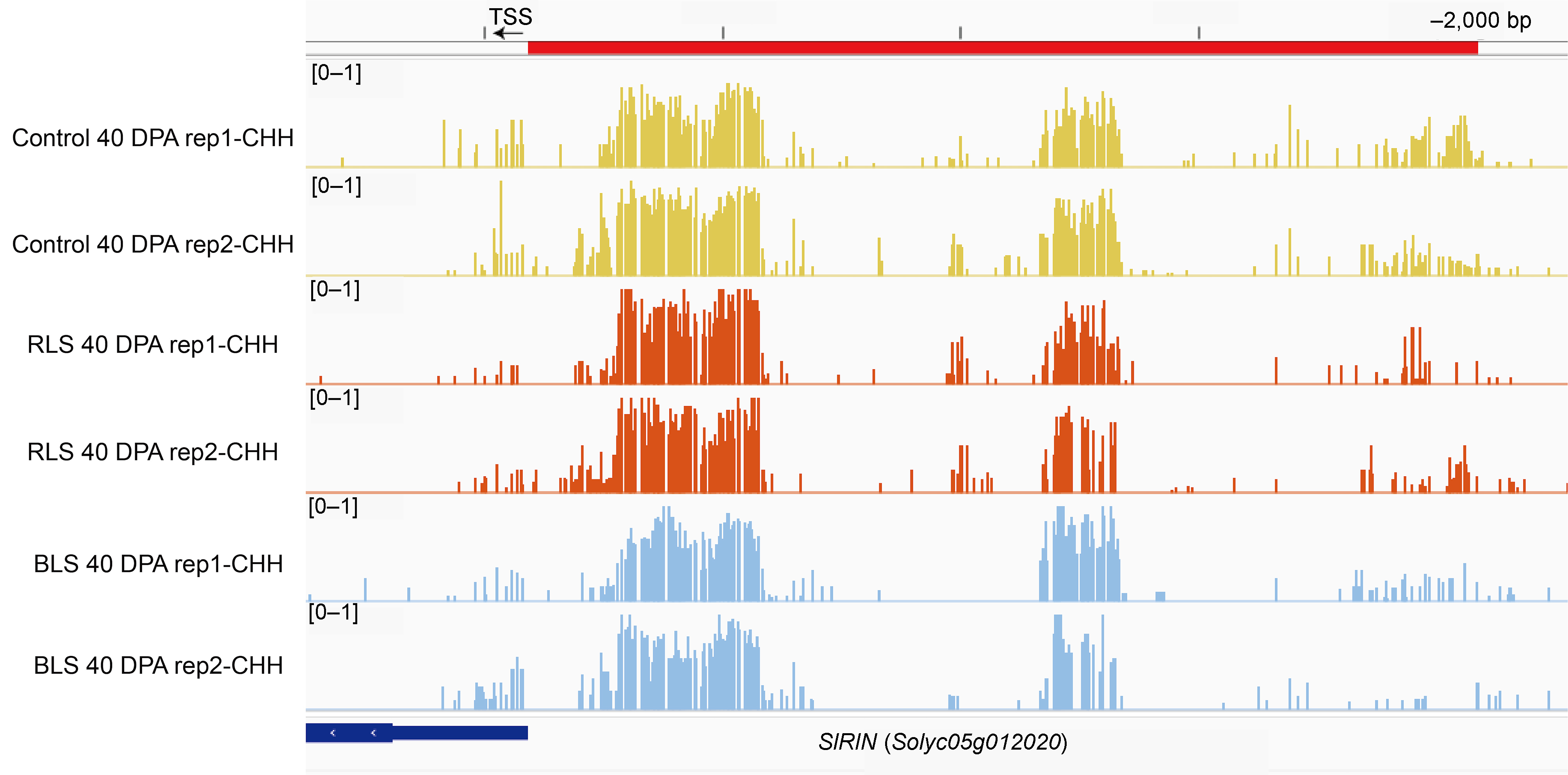


**Figure S8. mCHH methylation levels of *SlRIN* promoter in tomato at 40 DPA under three light conditions**

DNA methylation levels of 2,000 bp upstream promoter regions of *SlRIN*. The screenshots of Integrative Genomics Viewer (IGV) display whole-genome CHH methylation levels, where each vertical bar represents an mCHH and the height of the bar indicates methylation level.

**
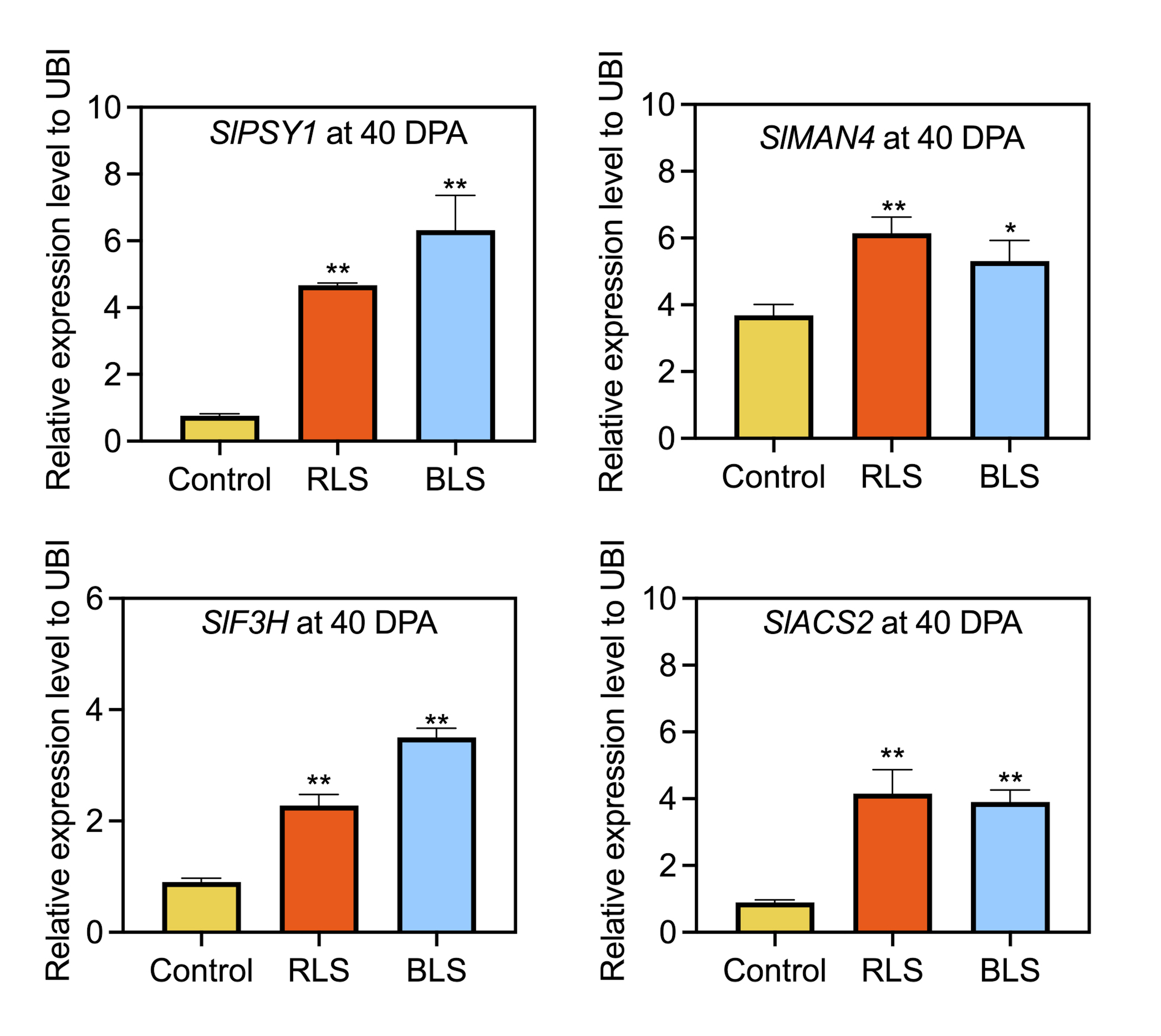
**

**Figure S9. Relative gene expression of key metabolic and ripening genes at 40 DPA**

*SlUBI* was used as the internal control, ***P <* 0.01 indicates significant differences compared to the control (Student’s *t*-test, *n* = 3).


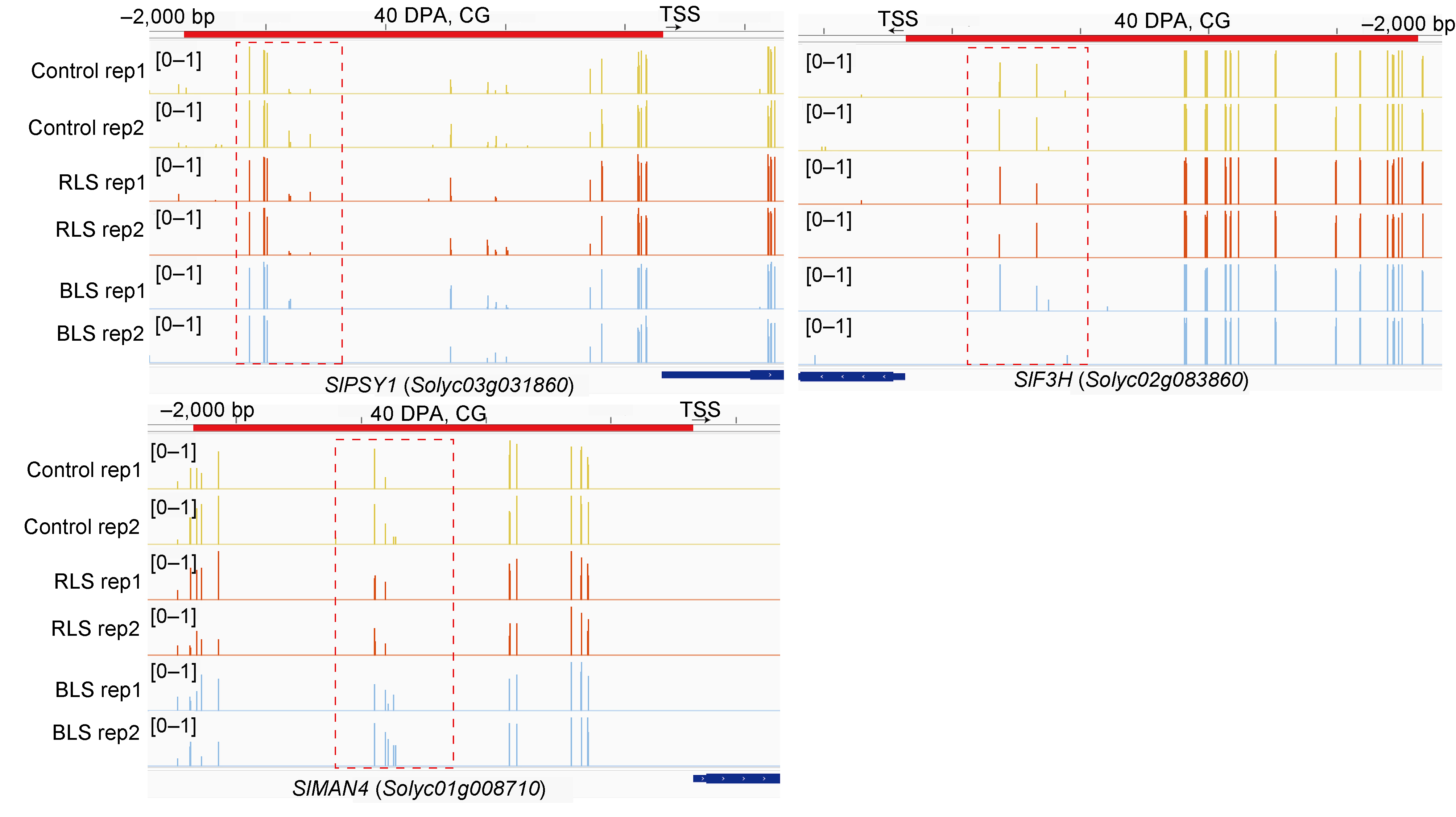


**Figure S10. The promoter CG methylation level of key metabolic and ripening genes at 40 DPA**

DNA methylation levels of 2,000 bp upstream promoter regions of these genes. The screenshots of Integrative Genomics Viewer (IGV) display whole-genome bisulfite sequencing data, where each vertical bar represents an mCG and the height of the bar indicates methylation level.

**
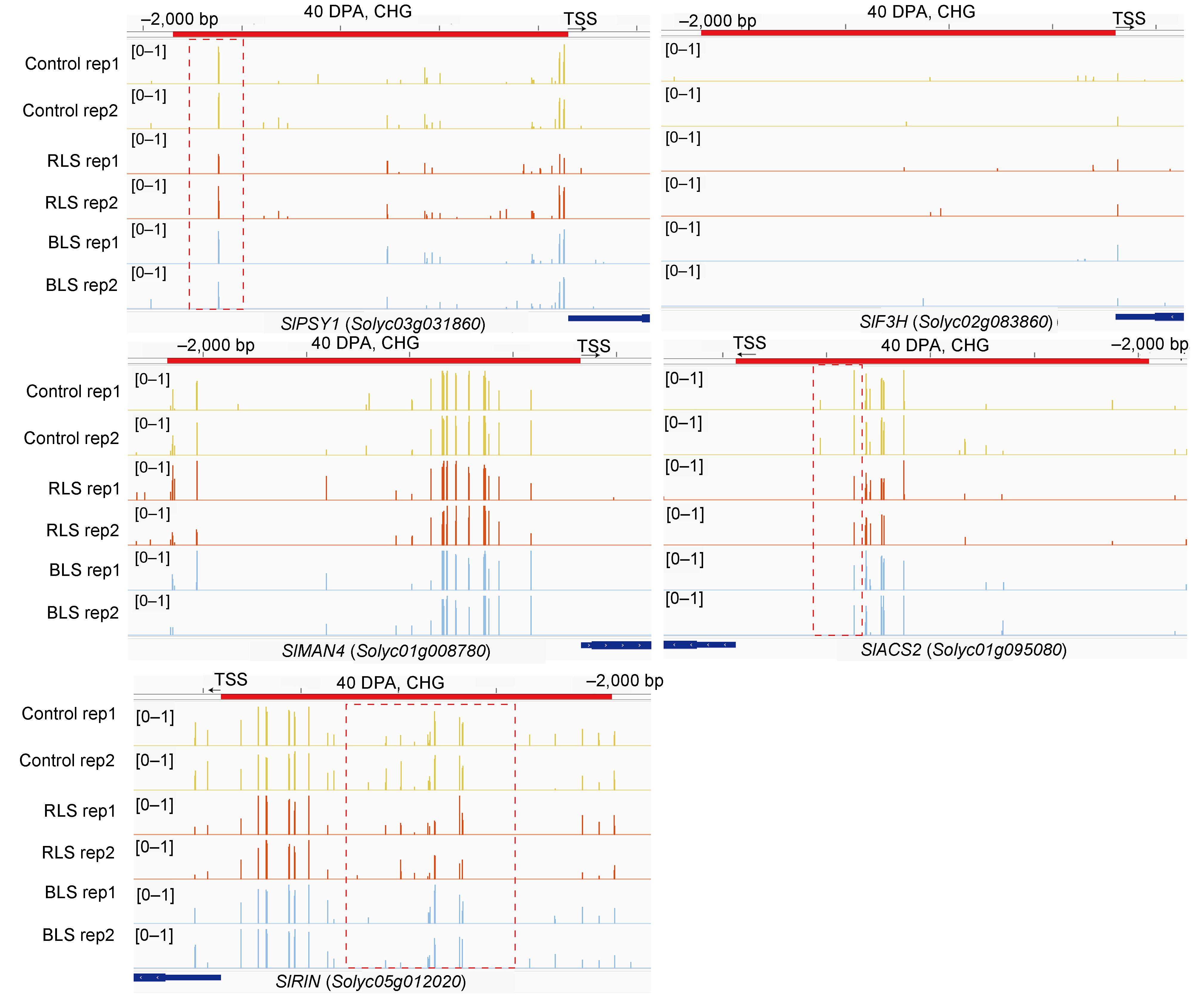
**

**Figure S11. The promoter CHG methylation level of key metabolic and ripening genes at 40 DPA**

DNA methylation levels of 2,000 bp upstream promoter regions of these genes. The screenshots of Integrative Genomics Viewer (IGV) display whole-genome bisulfite sequencing data, where each vertical bar represents an mCHG and the height of the bar indicates methylation level.

**
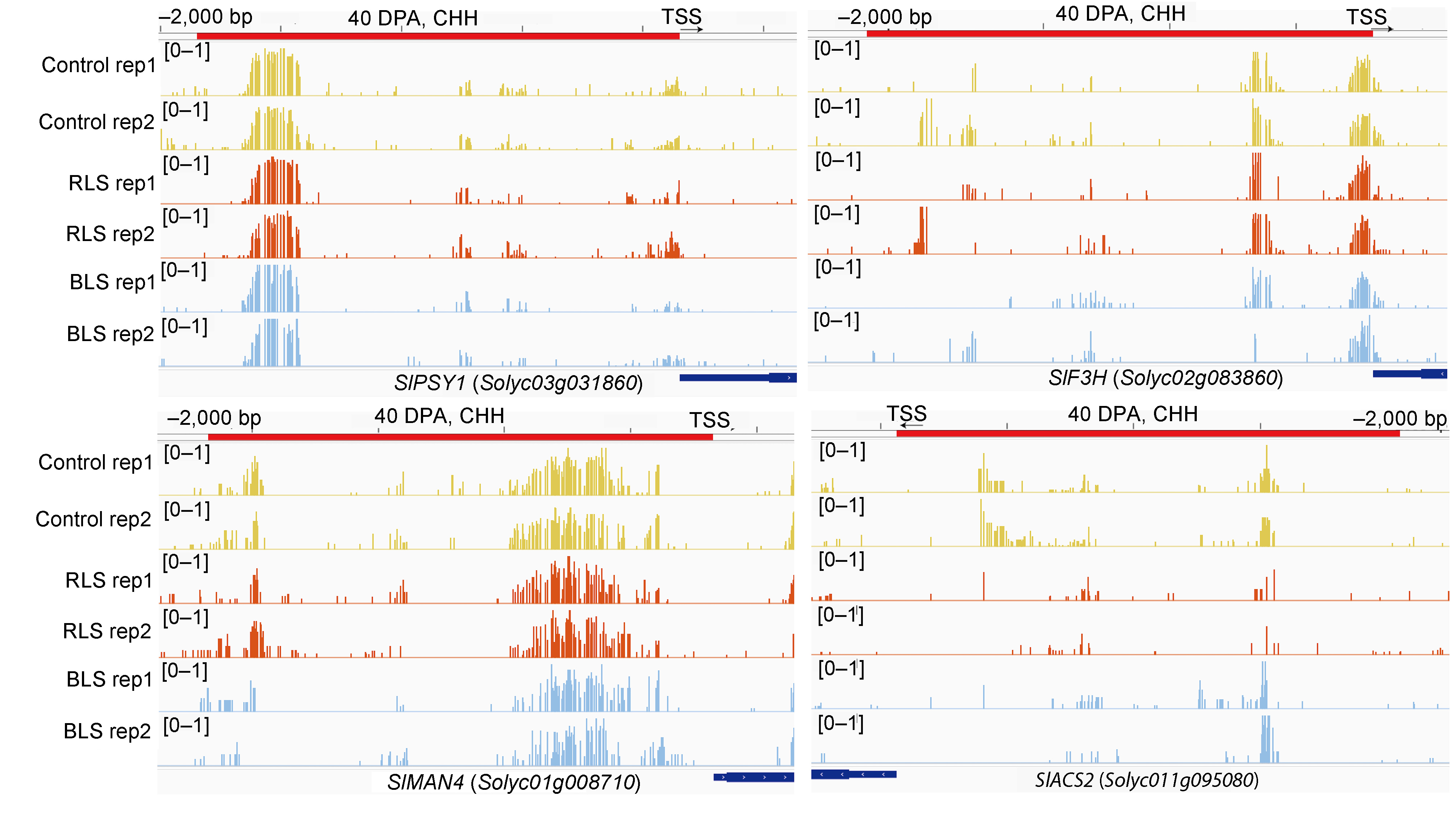
**

**Figure S12. The gene expression and promoter CHH methylation level of key metabolic and ripening genes at 40 DPA**

DNA methylation levels of 2,000 bp upstream promoter regions of these genes. The screenshots of Integrative Genomics Viewer (IGV) display whole-genome bisulfite sequencing data, where each vertical bar represents an mCHH and the height of the bar indicates methylation level.


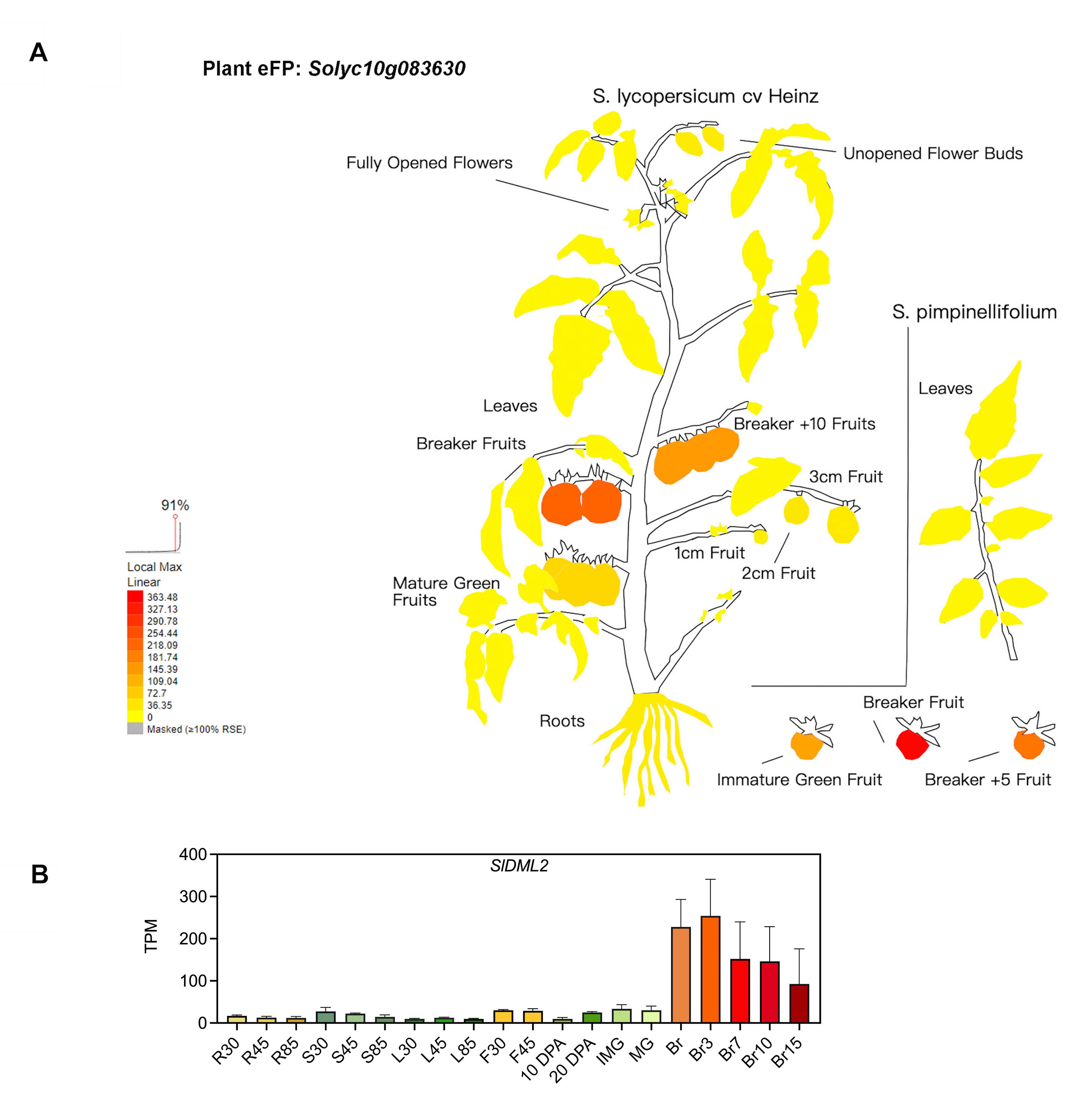


**Figure S13. The expression pattern of *SlDML2***

**(A)** Relative expression level of *SlDML2* in tomato plants using eplant website (http://bar.utoronto.ca/eplant_tomato/). **(B)** Relative expression of *SlDML2* in tomato at different developmental stages from MMN database. Error bars represent the standard deviation (*n* = 3). R, S, L and F represent root, stem, leaf and flower, respectively. IMG, MG and Br represent Immature green stage, mature green stage and breaker, respectively. Br3 (Breaker + 3), Br7 (Breaker + 7), Br10 (Breaker + 10), Br15 (Breaker + 15).

**
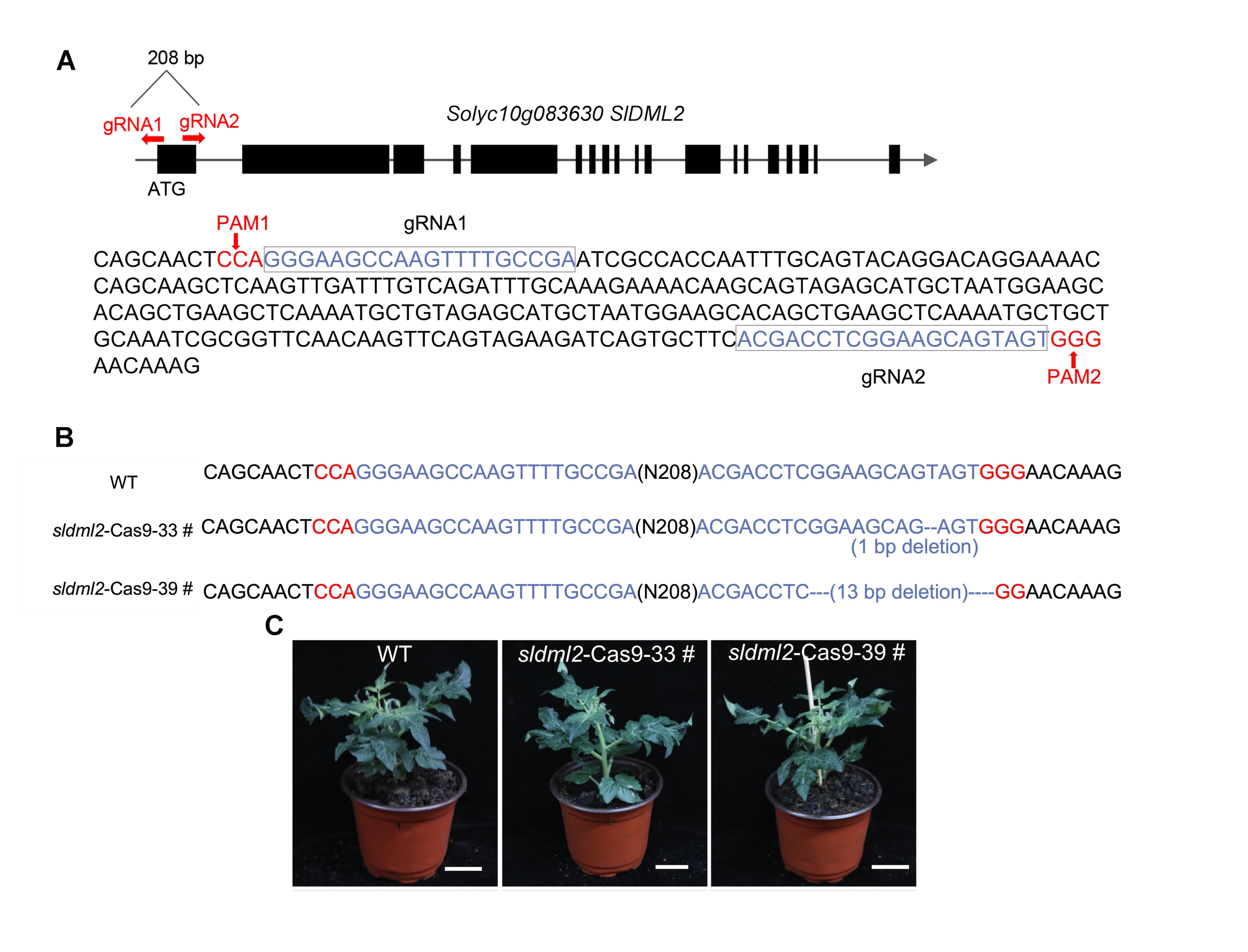
**

**Figure S14.** **Gene editing of *SlDML2***

**(A)** Gene editing target site selection. **(B)** Base editing analysis of *sldml2*-Cas9 plants. **(C)** Phenotypes of *sldml2*-cas9 T1 plants. Bars = 5 cm.


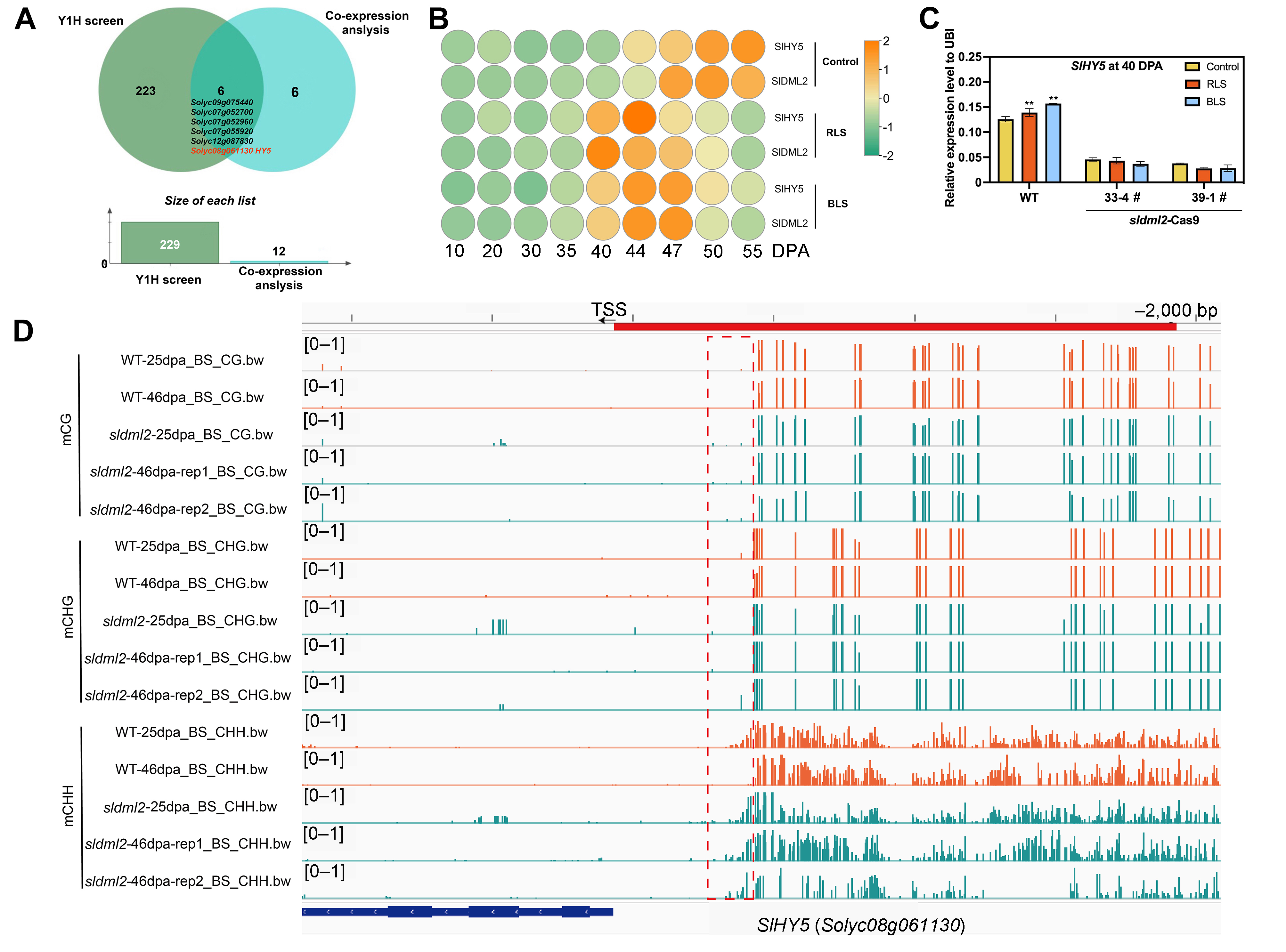


**Figure S15. The interaction between SlHY5 and *SlDML2***

**(A)** Venn diagram showing 6 genes (including *SlHY5*) can be screened by both Y1H screening of *proSlDML2* and co-expression analysis with *SlDML2*. **(B)** Heatmap of expression profiles of *SlHY5* and *SlDML2* under three light conditions. Z-scores of datasets were standardized to 2 to −2. The color orange represents 2, while green represents −2. **(C)** The red/blue light-induced expression of *SlHY5* is missing in the *sldml2*-Cas9 fruit at 40 DPA. *SlUBI* was used as the internal control, ***P <* 0.01 indicates significant differences compared to the control (Student’s *t*-test, *n* = 3). **(D)** DNA methylation level of *SlHY5* promoter in *sldml2* mutants and WT plants. The data was obtained from the previously published data by Lang et al, 2017.

**
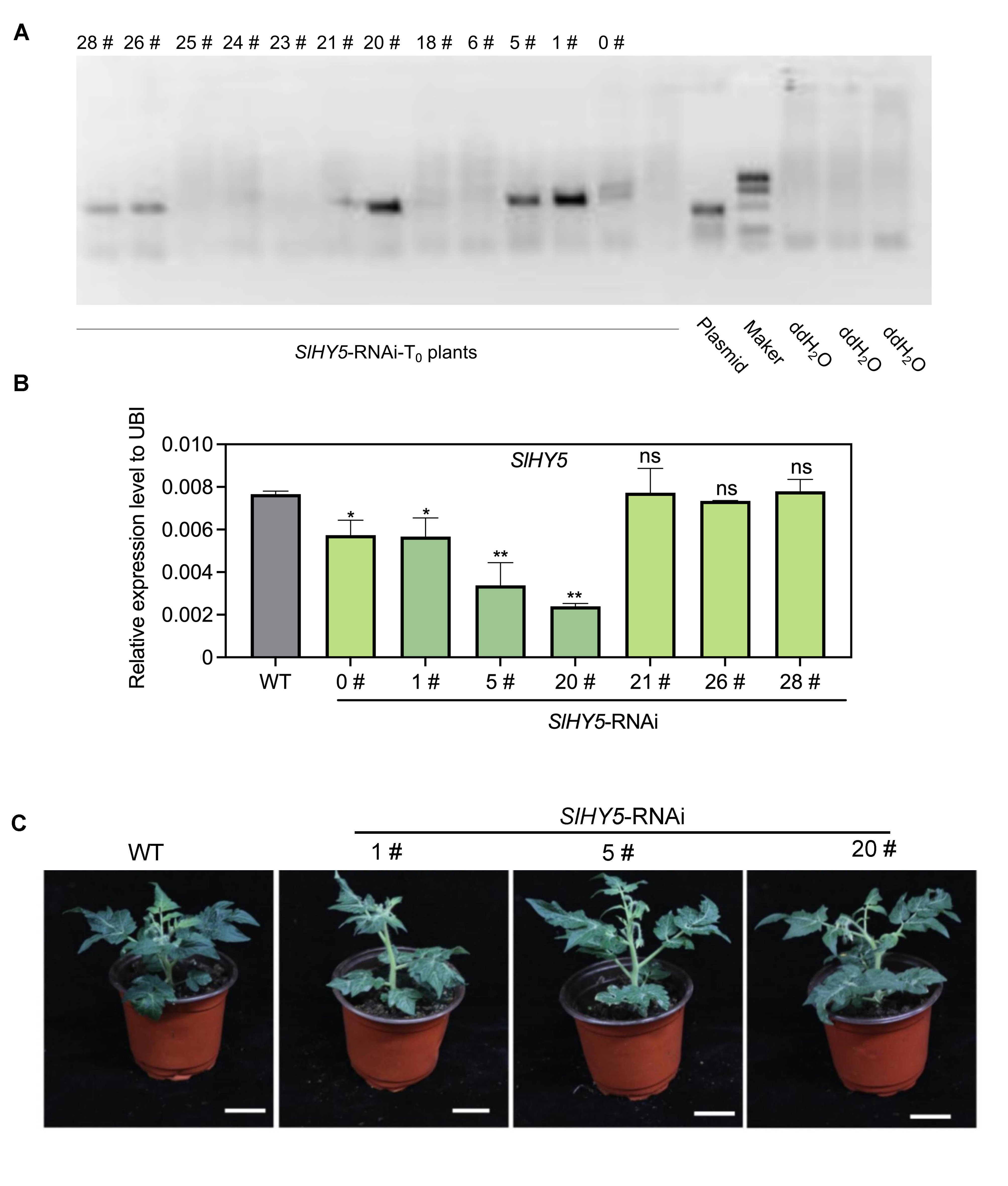
**

**Figure S16. The transgenic lines of *SlHY5***

**(A)** Identification of positive *SlHY*5-RNAi plants. **(B)** Relative gene expression level of *SlHY5* in the leaves of T0 RNAi seedlings *SlUBI* was used as the internal control. ***P* < 0.01 indicates significant differences between WT and RNAi plants (Student’s *t*-test, *n* = 3). **(C)** Phenotypes of *SlHY5*-RNAi plants. Bar = 5 cm.

**
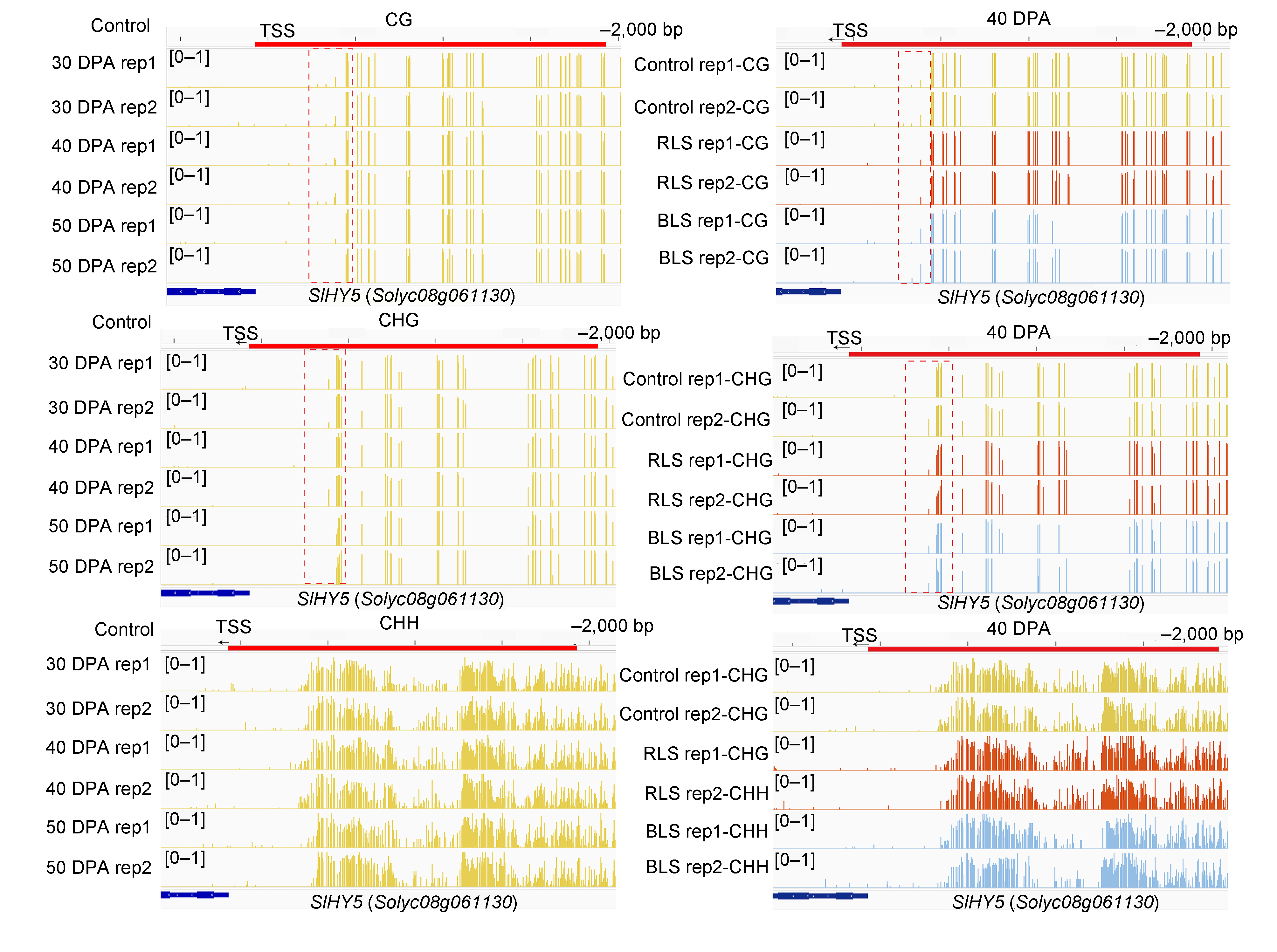
**

**Figure S17. DNA methylation levels of *SlHY5* promoters during fruit ripening process**

**(A)** mCG, mCHG and mCHH levels of *SlHY5* during fruit development and ripening under normal light conditions. **(B)** mCG, mCHG and mCHH levels of *SlHY5* in tomatoes at 40 DPA under three light conditions. The screenshots of Integrative Genomics Viewer (IGV) display whole-genome bisulfite sequencing data, where each vertical bar represents an mCG/mCHG/mCHH and the height of the bar indicates methylation level.

**
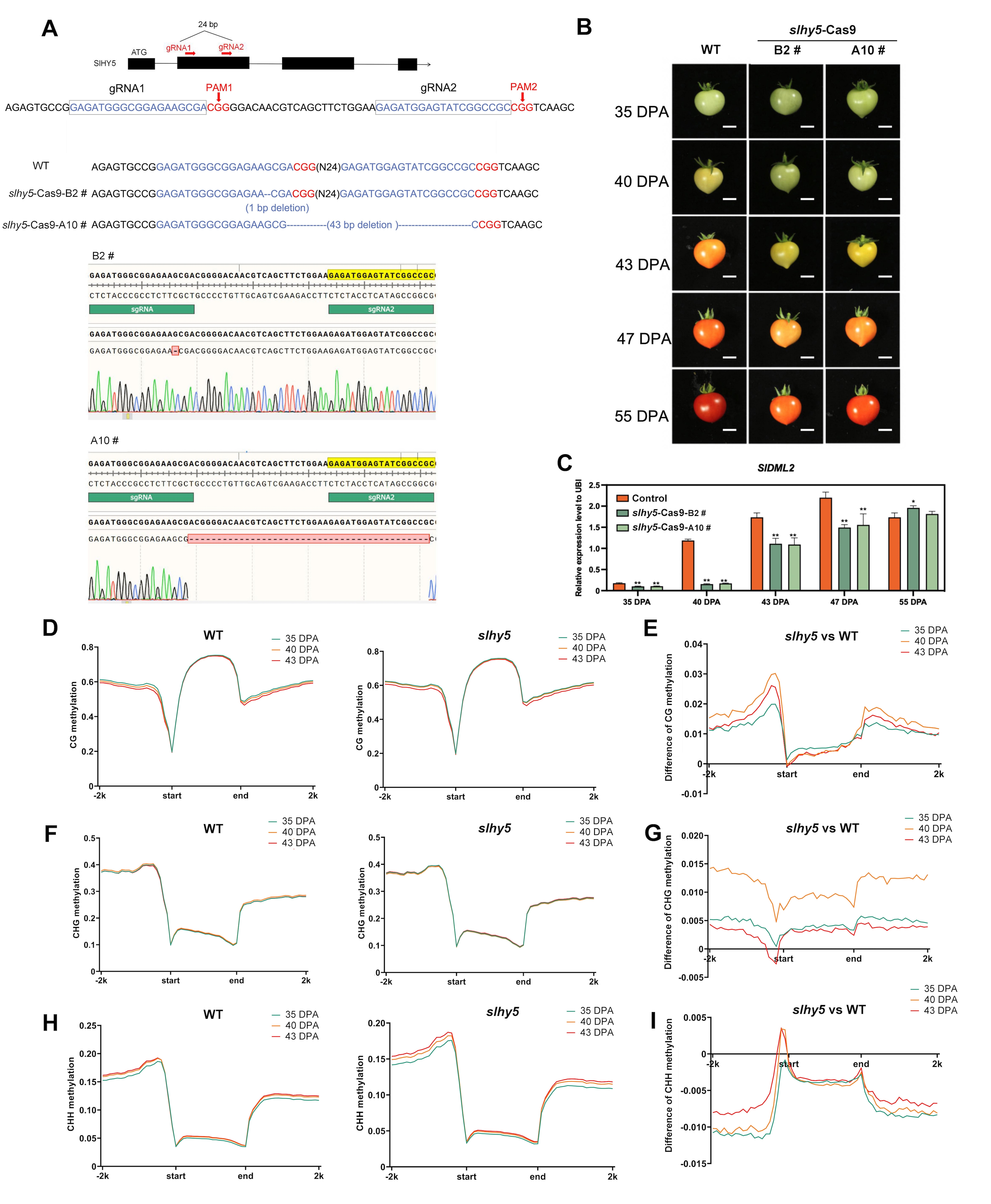
**

**Figure S18. SlDML2-mediated DNA demethylation was inhibited in *slhy5* plants**

**(A)** CRISPR/Cas9-mediated gene editing target site selection and base editing analysis of *slhy5-*Cas9 plants. Exons of the target genes are targeted by CRISPR/Cas9 using two single-guide RNAs (sgRNA, Target1 and target2 shown with red arrows). sgRNA targets and protospacer-adjacent motif (PAM) are indicated in blue and red, respectively. **(B)** Pictures of WT and *slhy5-*Cas9 fruits at 35 DPA (Day post anthesis), 40, 43, 47 and 55 DPA. Bar = 1 cm. **(C)** The relative gene expression levels of SlDML2 in *slhy5-*Cas9 fruits during fruit development and ripening. *SlUBI* was used as the internal control, ***P <* 0.01 indicates significant differences compared to the control (Student’s *t*-test, *n* = 3). **(D)** Genome wide of mCG methylations in WT and *slhy5*-Cas9 fruits at different developmental stages. **(E)** The difference of mCG methylation levels between *slhy5-*Cas9 and WT fruits during fruit development and ripening. **(F)** Genome wide of mCHG methylations in WT and *slhy5-*Cas9 fruits at different developmental stages. **(G)** The difference of mCHG methylation levels between *slhy5-*Cas9 and WT fruits during fruit development and ripening. **(H)** Genome wide of mCHH methylations in WT and *slhy5-*Cas9 fruits at different developmental stages. **(I)** The difference of mCHH methylation levels between *slhy5-*Cas9 and WT fruits during fruit development and ripening.

**
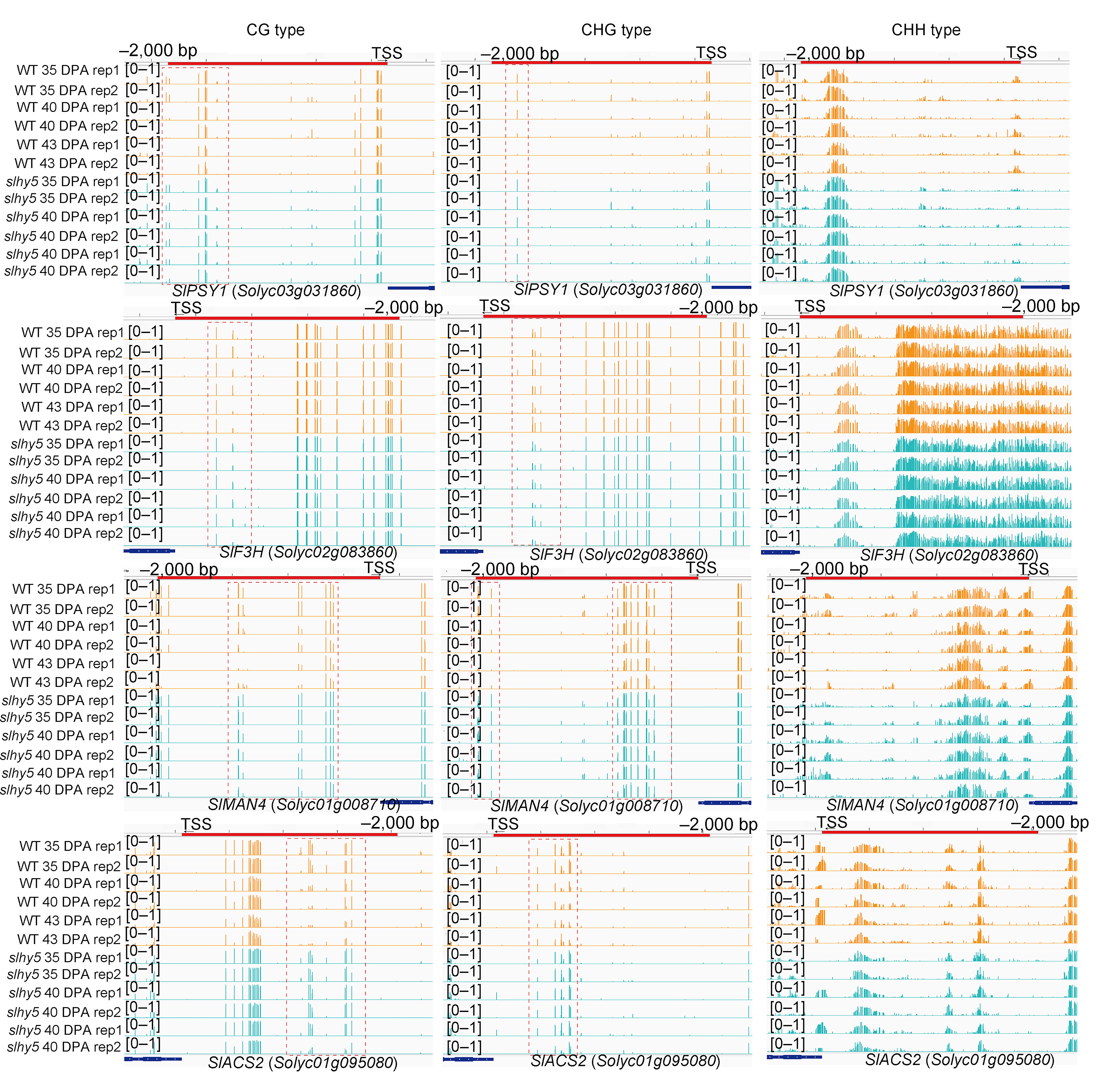
**

**Figure S19. The DNA demethylation process of the promoter of key metabolic and ripening genes was significantly delayed in the *slhy5*-Cas9 lines**

DNA methylation levels of 2,000 bp upstream promoter regions of these genes were shown with screenshots of Integrative Genomics Viewer (IGV) display of whole-genome bisulfite sequencing data, where each vertical bar represents an mCG/mCHG/mCHH and the height of the bar indicates methylation level.

**
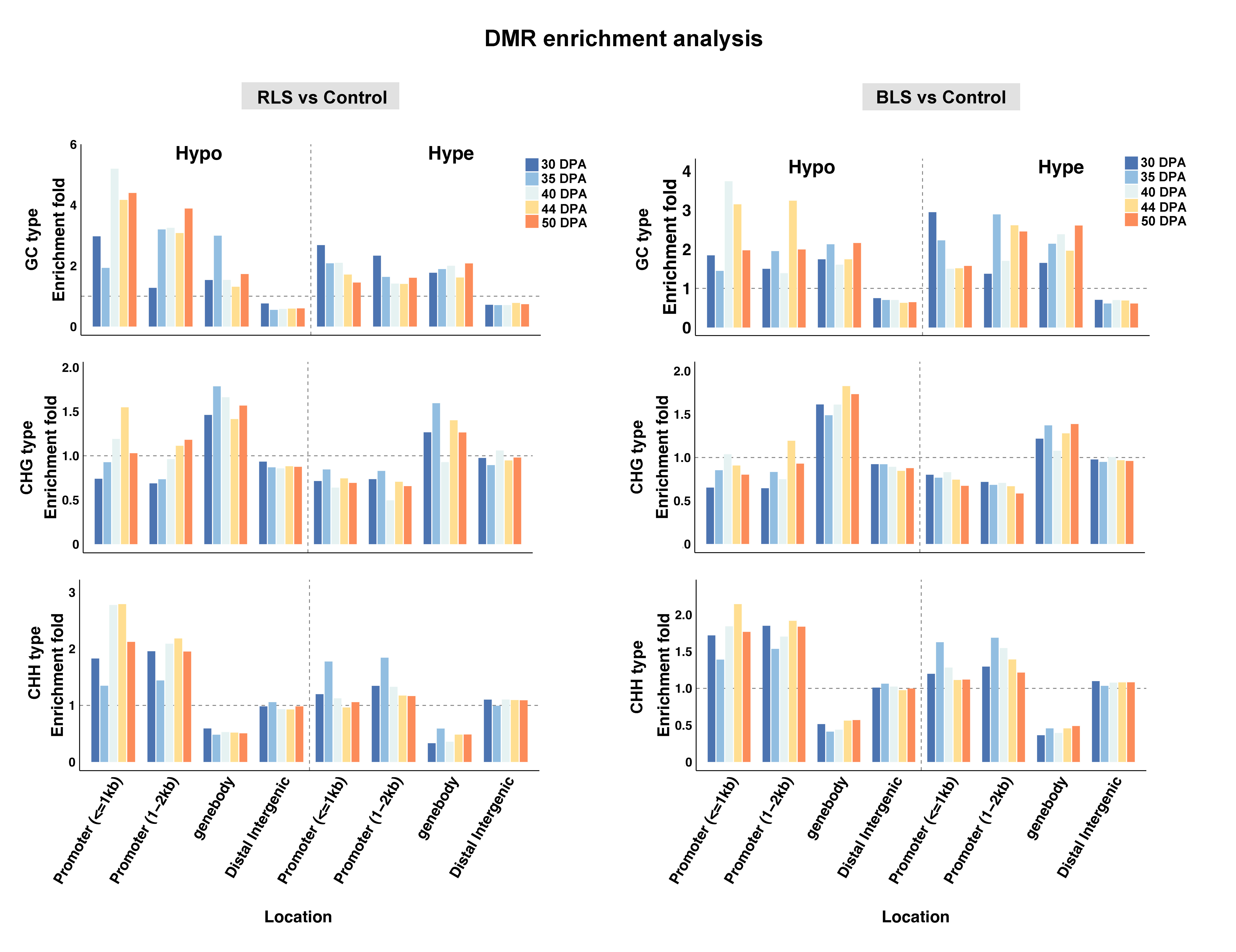
**

**Figure S20. Enrichment of DMRs across genomic regions**

The distribution of differentially methylated regions (DMRs), including hypomethylated (hypo) and hypermethylated (hyper) regions, was analyzed across genomic features (promoters, gene bodies, and distal intergenic regions) under different light treatments. Enrichment fold was calculated by comparing the observed distribution with that of randomly shuffled regions. The x-axis indicates genomic features, and the y-axis indicates the enrichment fold. *n* indicates the number of DMRs identified in each context. The horizontal grey dashed line indicates a fold enrichment of 1 (no enrichment).

**
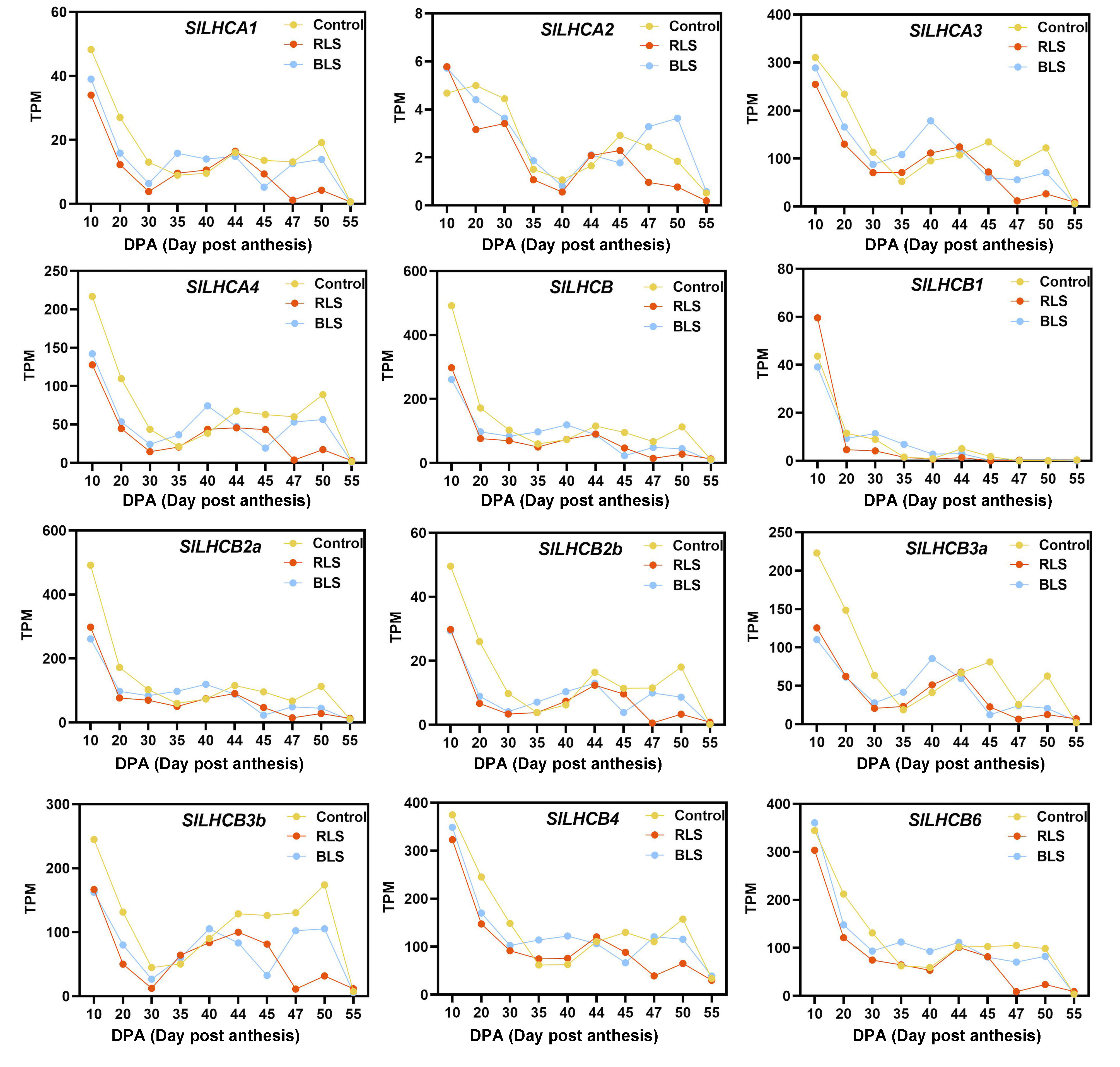
**

**Figure S21. The expression level of genes related to photosynthesis under three light conditions in the TomLED**


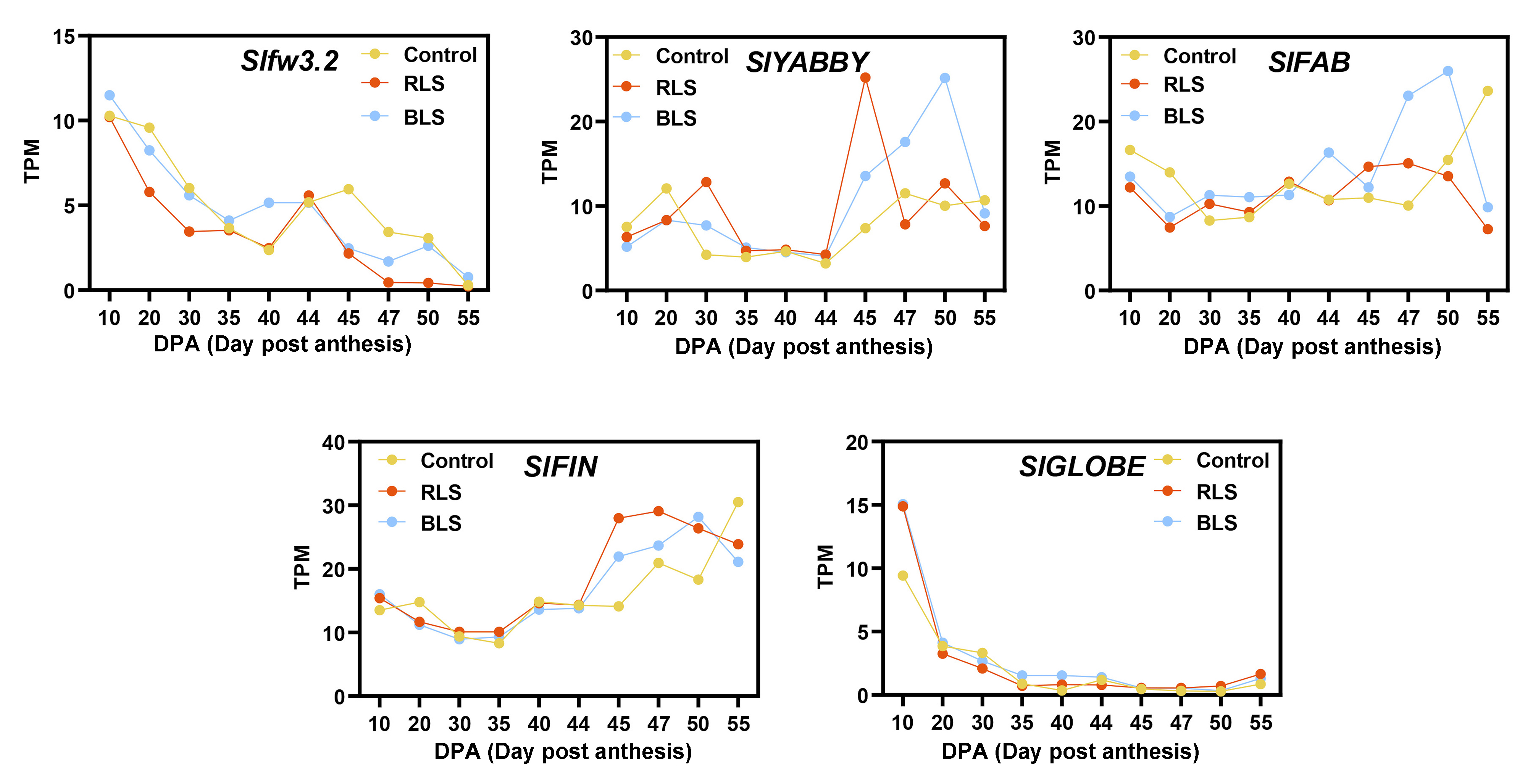


**Figure S22. The expression level of genes related to weight and fruit diameter under three light conditions in the TomLED**
