# Supplementary figures and images for: Light regulates tomato fruit metabolome via SlDML2‐mediated global DNA demethylation
Source: J Integr Plant Biol. 2025 Oct 23;68(2):383–405. doi: 10.1111/jipb.70066 (PMC12863027; doi:10.1111/jipb.70066)

## Slide 1
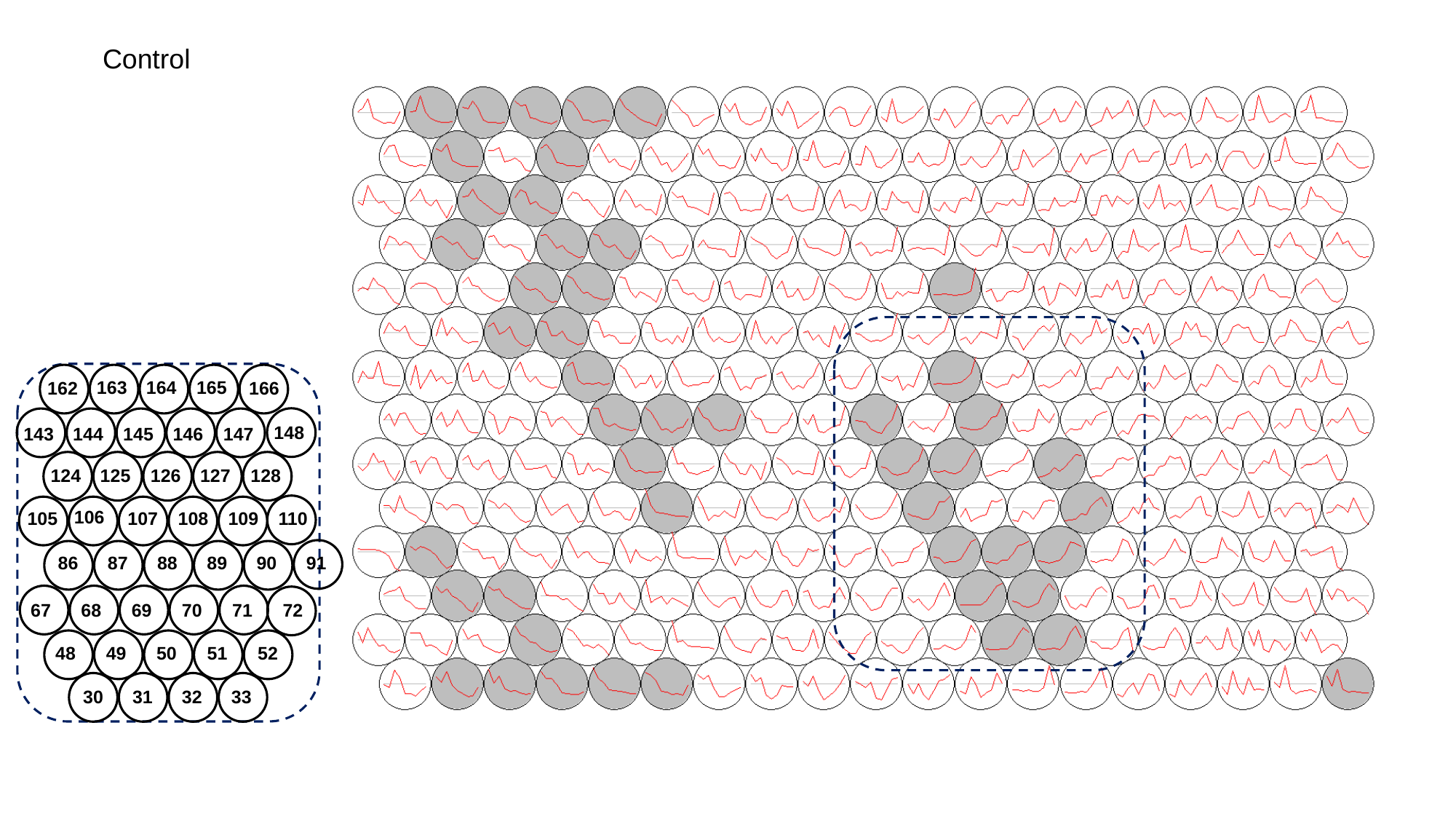

Control
163
164
165
162
166
148
143
144
145
146
147
124
125
126
127
128
106
105
107
108
109
110
86
87
88
89
90
91
67
68
69
70
71
72
48
49
50
51
52
30
31
32
33

## Slide 2
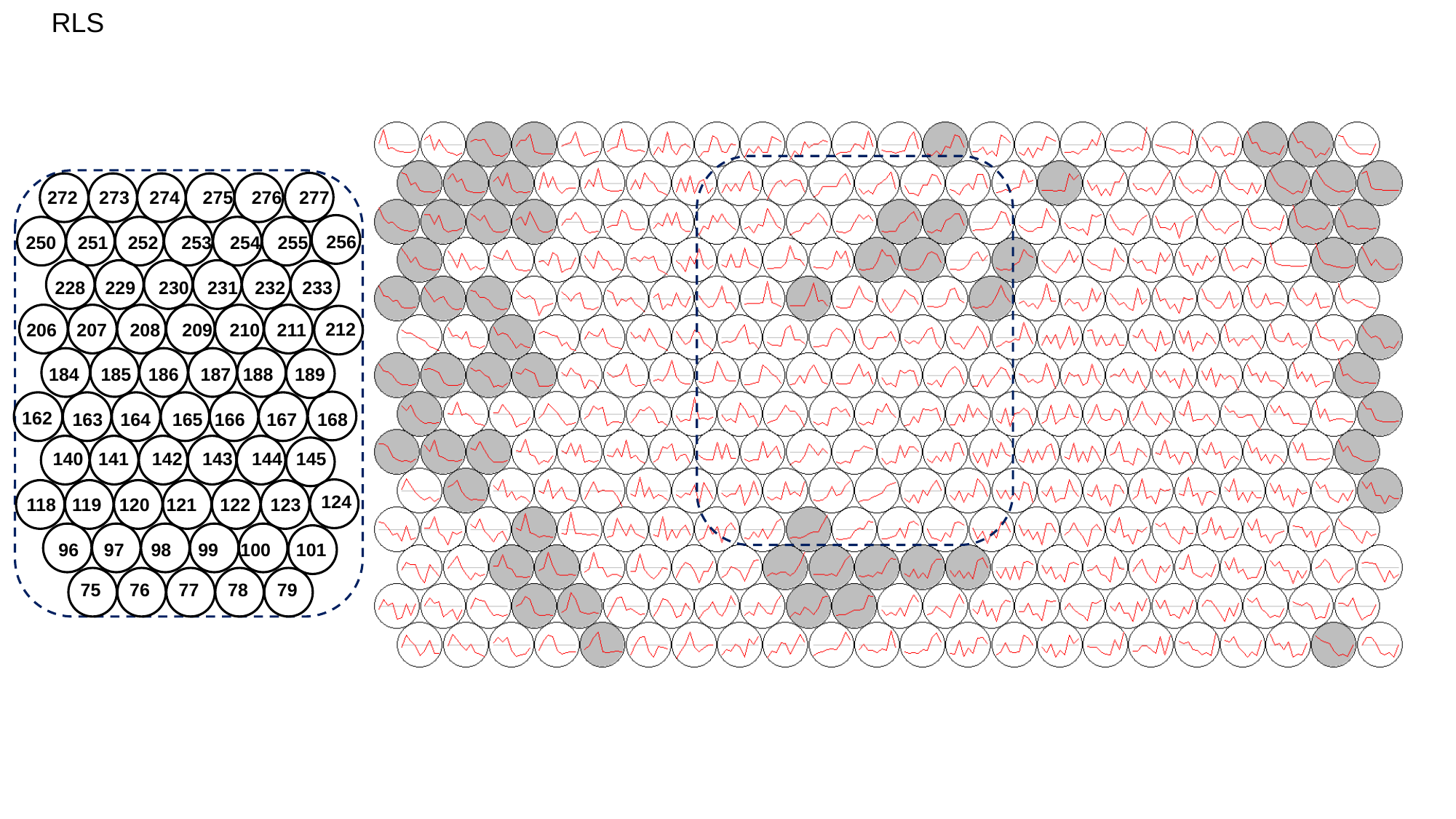

RLS
272
273
274
275
276
277
250
251
252
253
254
255
228
229
230
231
232
233
206
207
208
209
210
211
184
185
186
187
188
189
163
164
165
166
167
168
140
141
142
143
144
145
118
119
120
121
122
123
96
97
98
99
100
101
75
76
77
78
79
256
212
162
124

## Slide 3
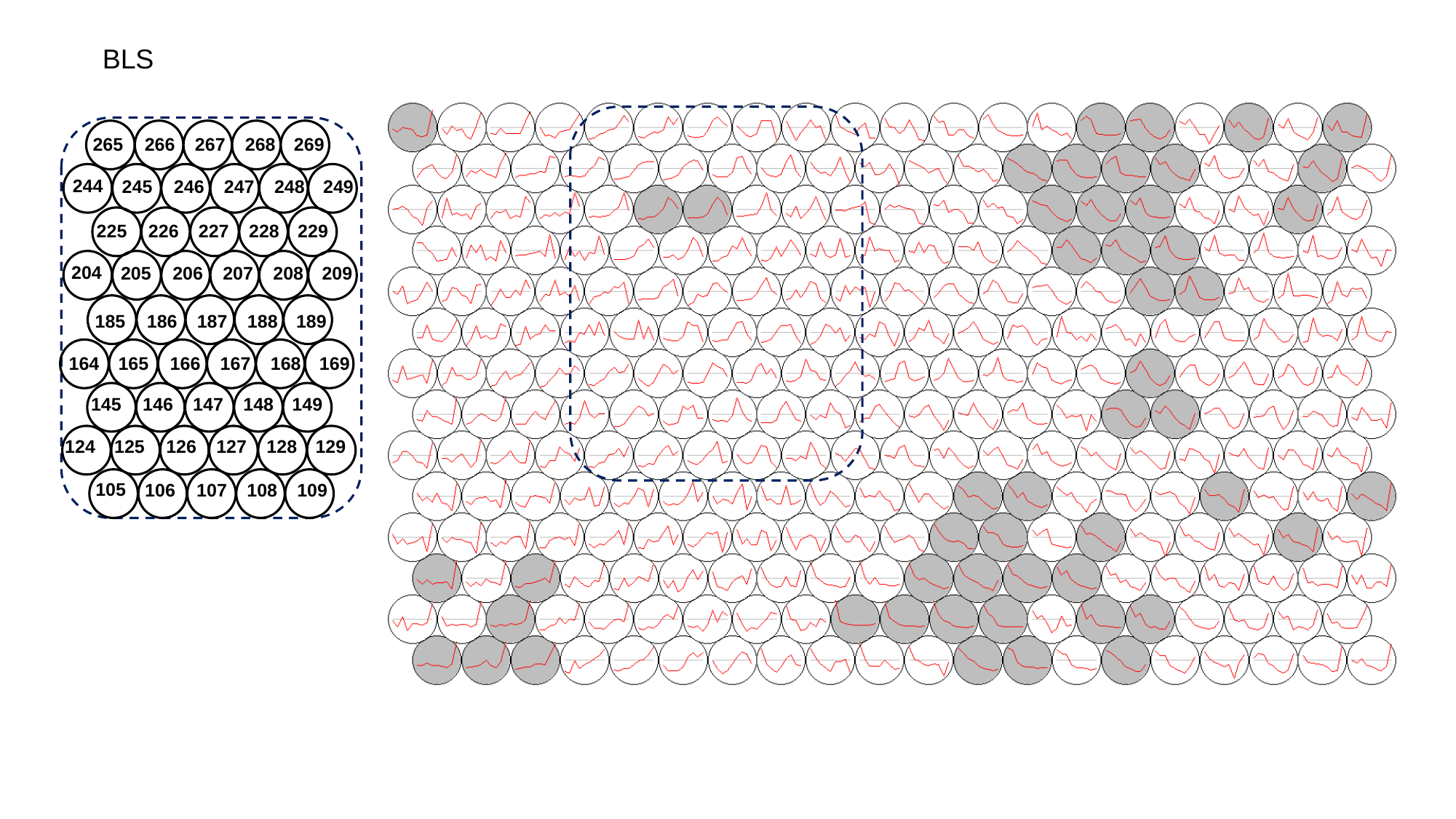

BLS
265
266
267
268
269
244
245
246
247
248
249
225
226
227
228
229
204
205
206
207
208
209
185
186
187
188
189
164
165
166
167
168
169
145
146
147
148
149
124
125
126
127
128
129
105
106
107
108
109

Supplement: Supplementary file 6 — Table S5. Summary of SOM [file JIPB-68-383-s011.zip › Supplemental Data 5 Summary of SOM/SOM.pptx]

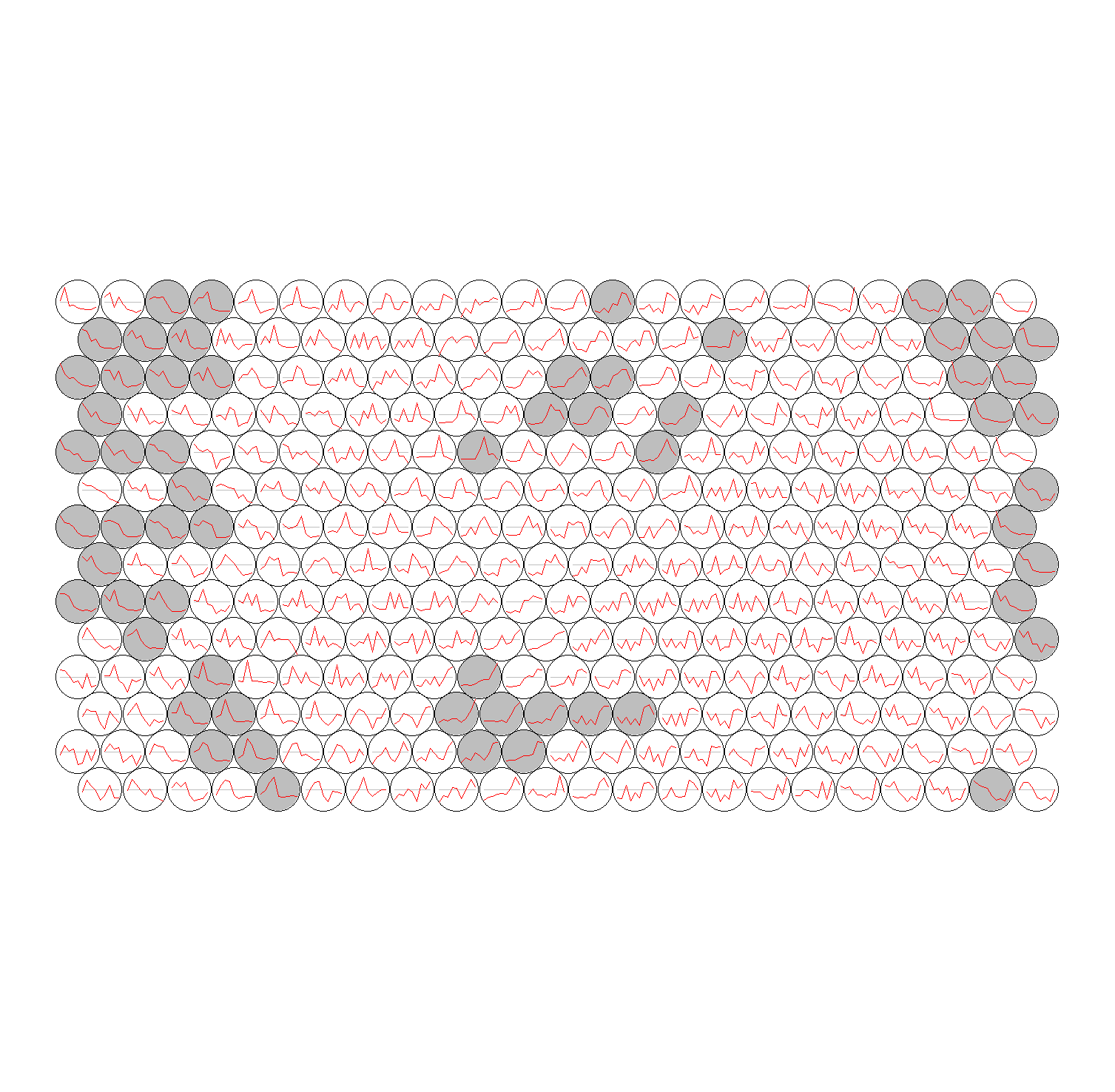

Supplement: Supplementary file 6 — Table S5. Summary of SOM [file JIPB-68-383-s011.zip › Supplemental Data 5 Summary of SOM/RLS/20230604_RLS_1.jpg]

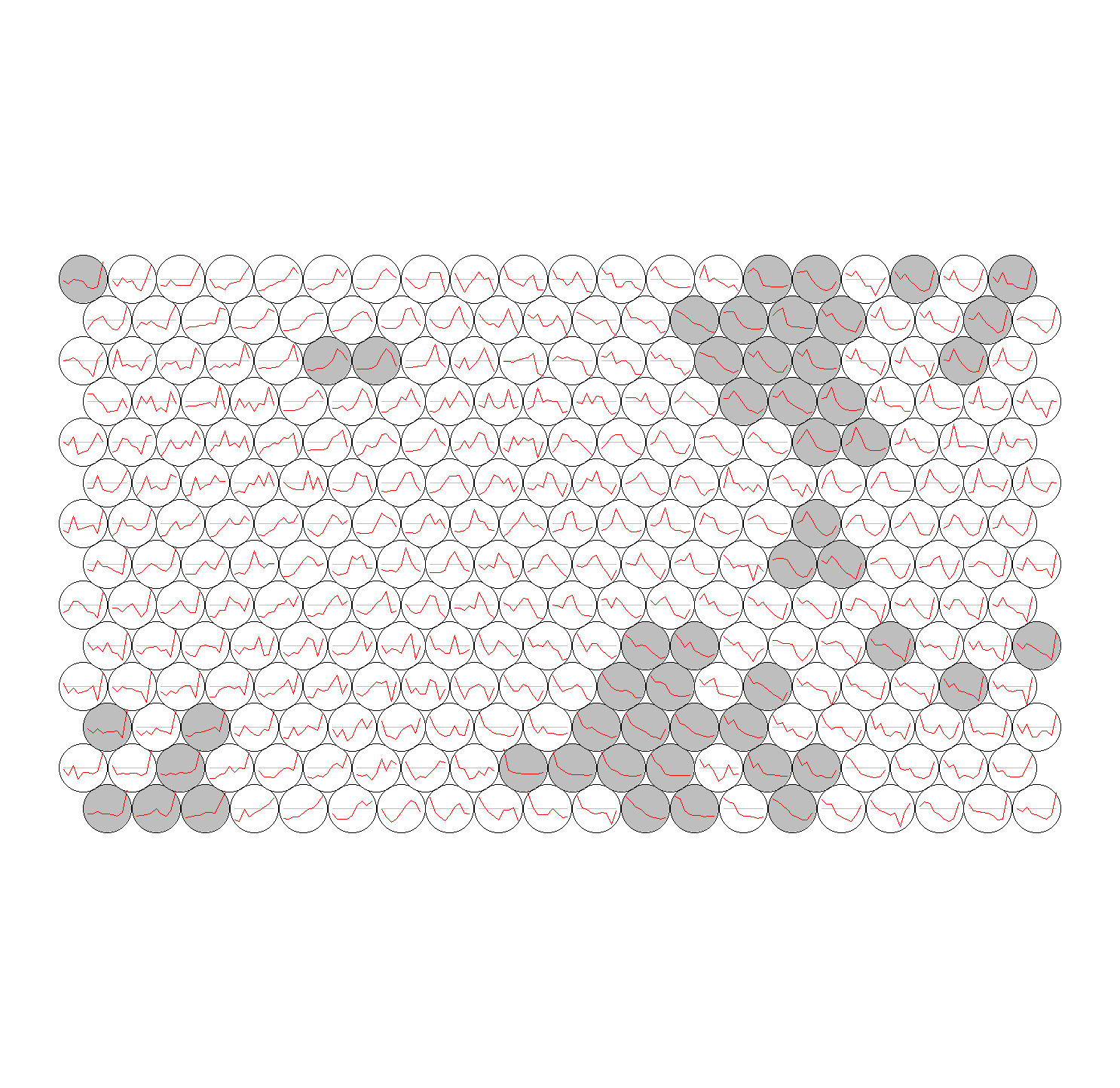

Supplement: Supplementary file 6 — Table S5. Summary of SOM [file JIPB-68-383-s011.zip › Supplemental Data 5 Summary of SOM/BLS/20230604_BLS_1.jpg]

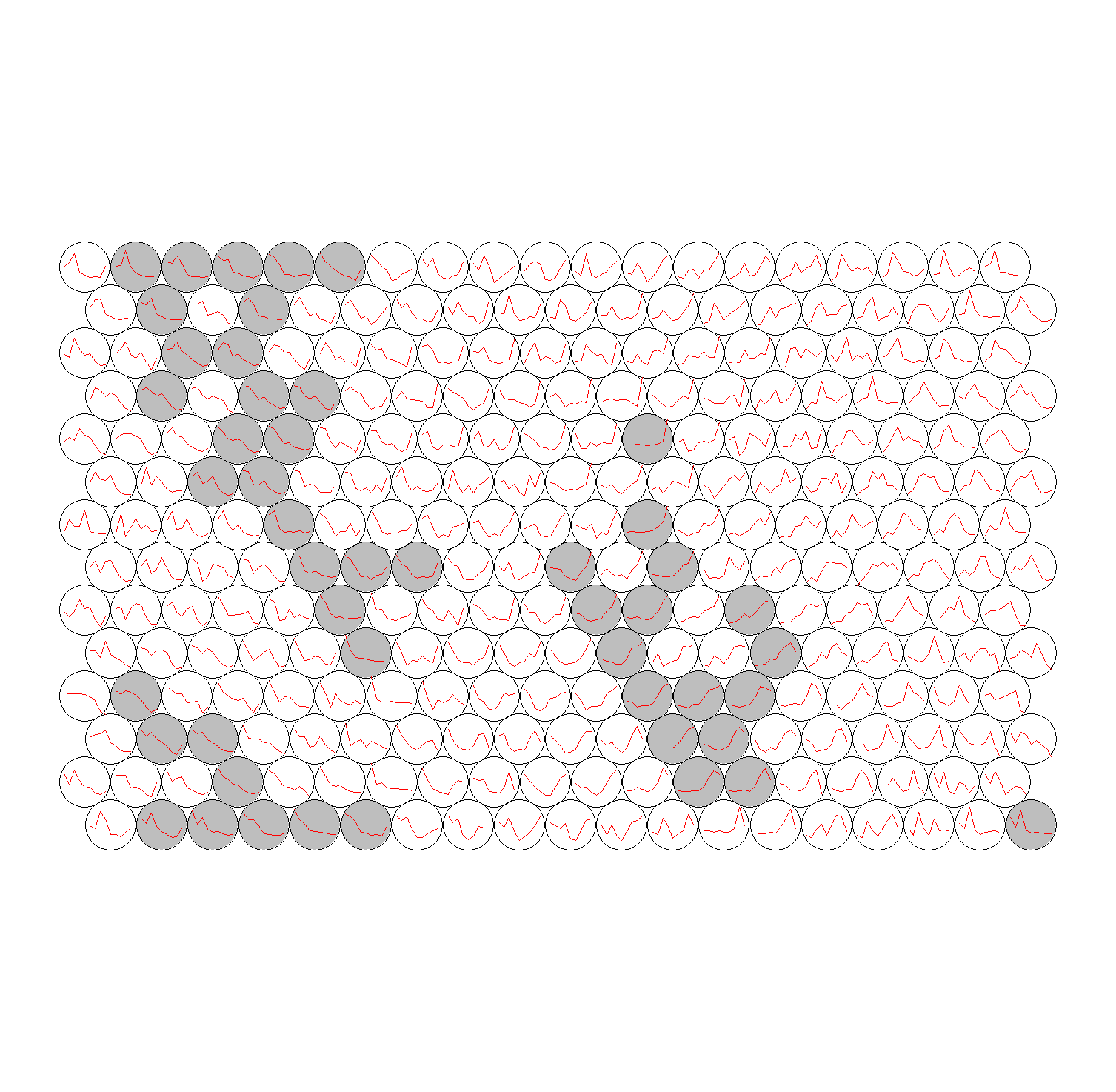

Supplement: Supplementary file 6 — Table S5. Summary of SOM [file JIPB-68-383-s011.zip › Supplemental Data 5 Summary of SOM/Control/20230604_Control_1.jpg]
